# Effect of an Interdisciplinary Weight Loss and Lifestyle Intervention on Obstructive Sleep Apnea Severity: The INTERAPNEA Randomized Clinical Trial

**DOI:** 10.1001/jamanetworkopen.2022.8212

**Published:** 2022-04-22

**Authors:** Almudena Carneiro-Barrera, Francisco J. Amaro-Gahete, Alejandro Guillén-Riquelme, Lucas Jurado-Fasoli, Germán Sáez-Roca, Carlos Martín-Carrasco, Gualberto Buela-Casal, Jonatan R. Ruiz

**Affiliations:** 1 Sleep and Health Promotion Laboratory, Mind, Brain and Behavior Research Centre, University of Granada, Granada, Spain; 2 Clinical Psychophysiology and Health Promotion Research Group, Ciencias y Técnicas de la Salud 261, University of Granada, Granada, Spain; 3 Department of Personality, Evaluation and Psychological Treatment, Faculty of Psychology, University of Granada, Granada, Spain; 4 Promoting Fitness and Health Through Physical Activity Research Group, Sport and Health University Research Institute, Department of Physical Education and Sports, Faculty of Sport Sciences, University of Granada, Granada, Spain; 5 EFFECTS-262 Research Group, Department of Medical Physiology, School of Medicine, University of Granada, Granada, Spain; 6 Unidad de Trastornos Respiratorios del Sueño, Servicio de Neumología, Hospital Universitario Virgen de las Nieves, Granada, Spain

## Abstract

**Importance:**

Obesity is the leading cause of obstructive sleep apnea (OSA); however, the effects of weight loss and lifestyle interventions on OSA and comorbidities remain uncertain.

**Objective:**

To evaluate the effect of an interdisciplinary weight loss and lifestyle intervention on OSA and comorbidities among adults with moderate to severe OSA and overweight or obesity.

**Design, Setting, and Participants:**

The Interdisciplinary Weight Loss and Lifestyle Intervention for OSA (INTERAPNEA) study was a parallel-group open-label randomized clinical trial conducted at a hospital-based referral center in Granada, Spain, from April 1, 2019, to October 23, 2020. The study enrolled 89 Spanish men aged 18 to 65 years with moderate to severe OSA and a body mass index (calculated as weight in kilograms divided by height in meters squared) of 25 or greater who were receiving continuous positive airway pressure (CPAP) therapy. The sole inclusion of men was based on the higher incidence and prevalence of OSA in this population, the differences in OSA phenotypes between men and women, and the known effectiveness of weight loss interventions among men vs women.

**Interventions:**

Participants were randomized to receive usual care (CPAP therapy) or an 8-week weight loss and lifestyle intervention involving nutritional behavior change, aerobic exercise, sleep hygiene, and alcohol and tobacco cessation combined with usual care.

**Main Outcomes and Measures:**

The primary end point was the change in the apnea-hypopnea index (AHI) from baseline to the intervention end point (8 weeks) and 6 months after intervention. Secondary end points comprised changes in other OSA sleep-related outcomes, body weight and composition, cardiometabolic risk, and health-related quality of life.

**Results:**

Among 89 men (mean [SD] age, 54.1 [8.0] years; all of Spanish ethnicity; mean [SD] AHI, 41.3 [22.2] events/h), 49 were randomized to the control group and 40 were randomized to the intervention group. The intervention group had a greater decrease in AHI (51% reduction; change, –21.2 events/h; 95% CI, –25.4 to –16.9 events/h) than the control group (change, 2.5 events/h; 95% CI, –2.0 to 6.9 events/h) at the intervention end point, with a mean between-group difference of –23.6 events/h (95% CI, –28.7 to –18.5 events/h). At 6 months after intervention, the reduction in AHI was 57% in the intervention group, with a mean between-group difference of –23.8 events/h (95% CI, –28.3 to –19.3 events/h). In the intervention group, 18 of 40 participants (45.0%) no longer required CPAP therapy at the intervention end point, and 6 of 40 participants (15.0%) attained complete OSA remission. At 6 months after intervention, 21 of 34 participants (61.8%) no longer required CPAP therapy, and complete remission of OSA was attained by 10 of 34 participants (29.4%). In the intervention vs control group, greater improvements in body weight (change, –7.1 kg [95% CI, −8.6 to −5.5 kg] vs −0.3 kg [95% CI, −1.9 to 1.4 kg]) and composition (eg, change in fat mass, −2.9 kg [95% CI, −4.5 to −1.3 kg] vs 1.4 kg [95% CI, −0.3 to 3.1 kg]), cardiometabolic risk (eg, change in blood pressure, −6.5 mm Hg [95% CI, −10.3 to −2.6 mm Hg] vs 2.2 mm Hg [95% CI, −2.1 to 6.6 mm Hg]), and health-related quality of life (eg, change in Sleep Apnea Quality of Life Index, 0.8 points [95% CI, 0.5-1.1 points] vs 0.1 points [95% CI, −0.3 to 0.4 points]) were also found at the intervention end point.

**Conclusions and Relevance:**

In this study, an interdisciplinary weight loss and lifestyle intervention involving Spanish men with moderate to severe OSA and had overweight or obesity and were receiving CPAP therapy resulted in clinically meaningful and sustainable improvements in OSA severity and comorbidities as well as health-related quality of life. This approach may therefore be considered as a central strategy to address the substantial impact of this increasingly common sleep-disordered breathing condition.

**Trial Registration:**

ClinicalTrials.gov Identifier: NCT03851653

## Key Points

**Question:**

Is an interdisciplinary weight loss and lifestyle intervention combined with usual care (continuous positive airway pressure [CPAP] therapy) effective for the treatment of moderate to severe obstructive sleep apnea (OSA) in men with overweight or obesity?

**Findings:**

In this randomized clinical trial involving 89 Spanish men with moderate to severe OSA who had overweight or obesity and were receiving CPAP therapy, an 8-week interdisciplinary weight loss and lifestyle intervention significantly improved OSA severity and other outcomes compared with usual care alone. At 8 weeks, 45% of participants in the intervention group no longer required CPAP therapy; at 6 months, 62% of participants in the intervention group no longer required CPAP therapy.

**Meaning:**

This study’s findings suggest that this weight loss and lifestyle intervention might be considered as a central strategy to address OSA and comorbidities.

## Teaser

This randomized clinical trial assesses the effect of an interdisciplinary weight loss and lifestyle intervention on obstructive sleep apnea severity and comorbidities among Spanish men with obstructive sleep apnea and overweight or obesity who were receiving continuous positive airway pressure therapy.

## Introduction

Obstructive sleep apnea (OSA), characterized by recurrent sleep state–dependent upper airway collapse, is a globally recognized major public health problem affecting up to 936 million adults in the general population, with obesity as the leading cause.^1,2^ Obstructive sleep apnea has emerged as a primary target of medical research and practice owing to its increasing prevalence and association with increasing rates of obesity^3^ and its wide spectrum of clinical and socioeconomic consequences.^4,5,6^ The intermittent pharyngeal obstructions occurring during sleep result in long-term exposure to hypoxia, hypercapnia, increased sympathetic activity, oxidative stress, and systemic inflammation.^7^ Given these pathophysiological responses, OSA has been independently associated with substantial increases in the likelihood of hypertension, dyslipidemia, diabetes, life-threatening cardiovascular diseases, and all-cause death.^8,9,10,11,12^

The first-line treatment for OSA is the use of a continuous positive airway pressure (CPAP) device, which maintains upper airway patency through positive pressure applied with a nasal or oronasal interface.^13^ Although CPAP therapy is effective in reducing upper airway occlusions when used as prescribed, adherence rates are suboptimal, and long-term benefits remain uncertain.^13,14,15^ Although some studies have found that CPAP therapy has beneficial effects on blood pressure and insulin resistance,^16,17^ other large observational and experimental studies have reported no significant reductions in metabolic risk or cardiovascular events after long-term CPAP therapy,^15,18,19,20^ which suggests a complex and reciprocal interaction between OSA and obesity.^21^

Weight loss attained through alternative or combined behavioral interventions appears to substantially improve OSA and coexisting conditions among adults.^22,23,24,25,26,27,28,29^ However, previous clinical trials of alternative and behavioral interventions, although enlightening, have had limitations inherent to study design or methods, including but not limited to stringent eligibility criteria, limited reported outcomes, and/or nonrandomized allocation, which restricted the generalizability of results.^24,30^ Furthermore, weight loss has only been addressed through restricted diets or exercise, without using either a combination of both components or behavioral approaches to promote maintenance of benefits.^24^ Notably, no study has, to our knowledge, focused on alcohol avoidance and smoking cessation,^24^ which are well-established behavioral risk factors associated with the occurrence and worsening of OSA.^31,32^ The Interdisciplinary Weight Loss and Lifestyle Intervention for OSA (INTERAPNEA) randomized clinical trial sought to determine the effects of a novel interdisciplinary weight loss and lifestyle intervention on OSA severity and comorbidities among adults with moderate to severe OSA who had overweight or obesity and were receiving CPAP therapy.^33^

## Methods

### Study Design and Oversight

The INTERAPNEA study was an investigator-initiated parallel-group open-label randomized clinical trial designed to evaluate the effects of an 8-week interdisciplinary weight loss and lifestyle intervention combined with usual care (ie, CPAP therapy) vs usual care alone on OSA severity (measured by the apnea-hypopnea index [AHI]) and OSA-related comorbidities among adults with moderate to severe OSA. The clinical trial rationale, design, and methods have been published previously,^33^ and the full protocol and statistical analysis plan are available in Supplement 1. The study was registered and approved by all regulatory authorities and ethics committees of each collaborating center in Spain (University of Granada, Virgen de las Nieves University Hospital, and Junta de Andalucía), with all participants providing written informed consent. This study followed the Consolidated Standards of Reporting Trials (CONSORT) reporting guideline for randomized clinical trials.

### Study Population

Eligible participants were men aged 18 to 65 years with moderate to severe OSA (AHI ≥15 events/h of sleep) who were receiving CPAP therapy and had a body mass index (BMI; calculated as weight in kilograms divided by height in meters squared) of 25 or greater. The sole inclusion of men was based on the higher incidence and prevalence of OSA in this population,^2^ the well-established differences in OSA phenotypes between men and women,^34^ and the effectiveness of weight loss interventions among men vs women.^24,35,36,37^ Exclusion criteria were current participation in a weight loss program, presence of any psychological or psychiatric disorder, and coexistence of any other primary sleep disorder. The eligibility criteria were based on a thorough consideration of the potential generalizability of results; thus, no criteria regarding potential responsiveness, comorbidities, adherence rates, or use of nonhypnotic medications were established. The study sample therefore reflected the heterogeneity of men with OSA. Details about eligibility criteria and assessments used to ensure inclusion feasibility are provided in Supplement 1 and eMethods 1 in Supplement 2.

### Study Recruitment, Enrollment, and Randomization

Recruitment, enrollment, and randomization of participants were performed among 3 consecutive sets of 30 to 35 participants from April 1, 2019, to January 24, 2020, with a study completion date of October 23, 2020. Participants were recruited from the outpatient sleep-disordered breathing unit of the collaborating hospital (Virgen de las Nieves University Hospital, Granada, Spain). Before enrollment, potential participants received clinical and physical examinations and completed baseline measurements to ensure inclusion feasibility. Enrolled participants were successively randomized to receive either usual care (control group) or a weight loss and lifestyle intervention combined with usual care (intervention group). Randomization was performed via a computer-generated simple (unrestricted) randomization process.^38^

Given the nature of the intervention, participants and clinicians were aware of clinical trial group assignments after randomization. However, the research personnel responsible for data collection and analysis were blinded to group assignments at the follow-up visits. In addition, rigorous standardization procedures for data collection and intervention were followed to ensure internal and external validity of the clinical trial.^39^

### Study Assessments and End Points

Assessments at baseline, the intervention end point (8 weeks), and 6 months after intervention included a full-night polysomnography conducted in a laboratory, a set of questionnaires, a full-body dual-energy x-ray absorptiometry scan, and a fasting blood test. The primary end point of the INTERAPNEA clinical trial was the change in AHI at the intervention end point and 6 months after intervention, which was objectively measured during a level 1 sleep study (ie, polysomnography). The AHI measures the number of apnea and hypopnea events per hour of sleep, with 0 to 4 events indicating normal (no OSA), 5 to 14 events indicating mild OSA, 15 to 30 events indicating moderate OSA, and more than 30 events indicating severe OSA; a change of at least 15 events is considered clinically meaningful and would move a participant 2 levels in severity status (eg, from severe to mild OSA, indicating a benefit for health).

Secondary end points comprised changes in other sleep-related variables (including oxyhemoglobin desaturation index, oxygen saturation, sleep efficiency and maintenance, sleep architecture, and subjective sleep quality [measured by the Pittsburgh Sleep Quality Index^40^; score range, 0-21 points, with higher scores indicating worse sleep quality] and sleepiness [measured by the Epworth Sleepiness Scale^41^; score range, 0-24 points, with higher scores indicating more daytime sleepiness]), body weight and composition (including BMI; neck, chest, and waist circumference; visceral adipose tissue, and lean mass), and cardiometabolic risk measurements (including blood pressure, glucose and lipid metabolism, and liver function). Health-related quality of life (measured by the Sleep Apnea Quality of Life Index^42^ [score range, 1-7 points, with higher scores indicating better health-related quality of life] and the physical and mental components of the Medical Outcomes Study 36-Item Short-Form Health Survey^43,44^ [score range, 0 to 100 points, with higher scores indicating better health-related quality of life with respect to either the physical or mental component]) and lifestyle habits (including physical activity, dietary behavior [measured by the Food Behavior Checklist^45^; score range, 23-85 points, with higher scores indicating healthier dietary patterns], alcohol consumption, and smoking) were also included as additional end points. Primary and secondary sleep-related end points as well as self-reported end points related to general physical and psychological health were measured at each study assessment after 1 week without CPAP use.

All adverse events, regardless of severity or relationship to the study intervention or participation, were systematically recorded at each of the study assessments. Serious adverse events were determined based on the guidelines adopted by the International Conference on Harmonization of Good Clinical Practice.^46^ Full descriptions of study assessments and end points are provided in eMethods 2 in Supplement 2.

### Study Intervention and Control Condition

The interdisciplinary weight loss and lifestyle intervention was precisely designed and implemented based on previous research^24^ and existing evidence-based clinical practice guidelines for the management of obesity^47,48^ and OSA.^4,5,22,23^ Feasible implementation in clinical practice was also considered. As a result, the intervention was conducted for 8 weeks and comprised 5 components or modules: nutritional behavior change, moderate aerobic exercise, smoking cessation, alcohol intake avoidance, and sleep hygiene. Each component included group-based weekly sessions of 60 to 90 minutes that were led and supervised by trained professionals (A.C.-B., F.J.A.-G., and L.J.-F.) in each field. Participants in the intervention group also continued to receive usual care with CPAP therapy. A detailed intervention description, including assessment of intervention adherence and integrity, has previously been published^33^ and is also provided in eMethods 3 and eMethods 4 in Supplement 2.

In addition to usual care with CPAP therapy, participants randomized to the usual care group received general advice on weight loss and lifestyle changes from a sleep-disordered breathing specialist in a single 30-minute session. The opportunity to receive the INTERAPNEA clinical trial intervention was offered to all participants at the end of the study.

### Statistical Analysis

The sample size and power of the INTERAPNEA clinical trial were estimated based on previous studies synthesized in a recent systematic review and meta-analysis.^24^ Assuming an SD of 11.98 (the AHI pooled SD found in previous research^24^) for our primary end point, we estimated that enrollment of 35 participants per arm would provide statistical power of 90% at α = .05 to detect a minimum effect size of –8.36 (the pooled mean difference of previous clinical trials included in the meta-analysis^24^) for the AHI. However, considering a maximum study withdrawal rate of 17.25% (the mean withdrawal rate of previous studies in the meta-analysis^24^), we decided to recruit 42 participants for each study group. Owing to practical and feasibility reasons, the clinical trial was conducted among 3 consecutive sets of 30 to 35 participants.^33^

Intervention effects on primary and secondary end points were assessed using linear mixed-effects models, with individual measures of growth being modeled as the function of randomization group, assessment time (baseline, 8 weeks, and 6 months after intervention), and the interaction between group and time.^49^ Estimations were performed using the restricted maximum likelihood method, including an unstructured covariance matrix to adjust for within-participant clustering resulting from the repeated-measures design.

The model assumed that missing values were missing at random; therefore, all values presented in the tables were model-based estimates. Nevertheless, attrition propensity was calculated using a logistic model estimating attrition with baseline values of the set of participants, randomization group, OSA severity, age, and BMI. Among these variables, only the set of participants significantly estimated attrition, which was attributable to the emergence of the COVID-19 pandemic at the clinical trial end point (the end point assessment of the third set of participants). Thus, the assumption that missing values in the models were missing at random was further supported; the missing at random assumption has been advocated in recent recommendations for handling missing data in randomized clinical trials affected by a pandemic, which occurred during our study.^50^ Missing data in primary and secondary sleep-related end points and secondary body composition end points were the result of participants withdrawing from the study before completion. The number of missing values in secondary cardiometabolic risk end points, including glucose and lipid metabolism and liver function end points are provided in eTable 1 in Supplement 2.

In addition, the association between changes in BMI and changes in AHI over time was examined using repeated measures correlation analysis. This statistical technique is used to determine the within-individual association for paired measures assessed on 2 or more occasions among multiple individuals.^51^

All estimations and analyses were performed using an intention-to-treat approach (including all participants as they were originally randomized) and an additional per-protocol approach that was restricted to participants with CPAP use of 4 hours or more per night on 70% of nights and, among the intervention group, an 80% or greater attendance rate for intervention sessions. Hypothesis testing was 2-sided, with *P* < .05 considered statistically significant. All analyses were conducted using R software, version 4.0.3 (R Foundation for Statistical Computing); linear mixed-effects models were performed using the lme4 package for R software.^49^

Intervention effect assessments were based not only on statistical and practical significance but on a practical benefit approach emphasizing and reporting unadjusted values that are intuitive to human judgment and readily replicable considering the design and methods used in this study.^52,53^

## Results

### Study Participants

**Figure 1.**
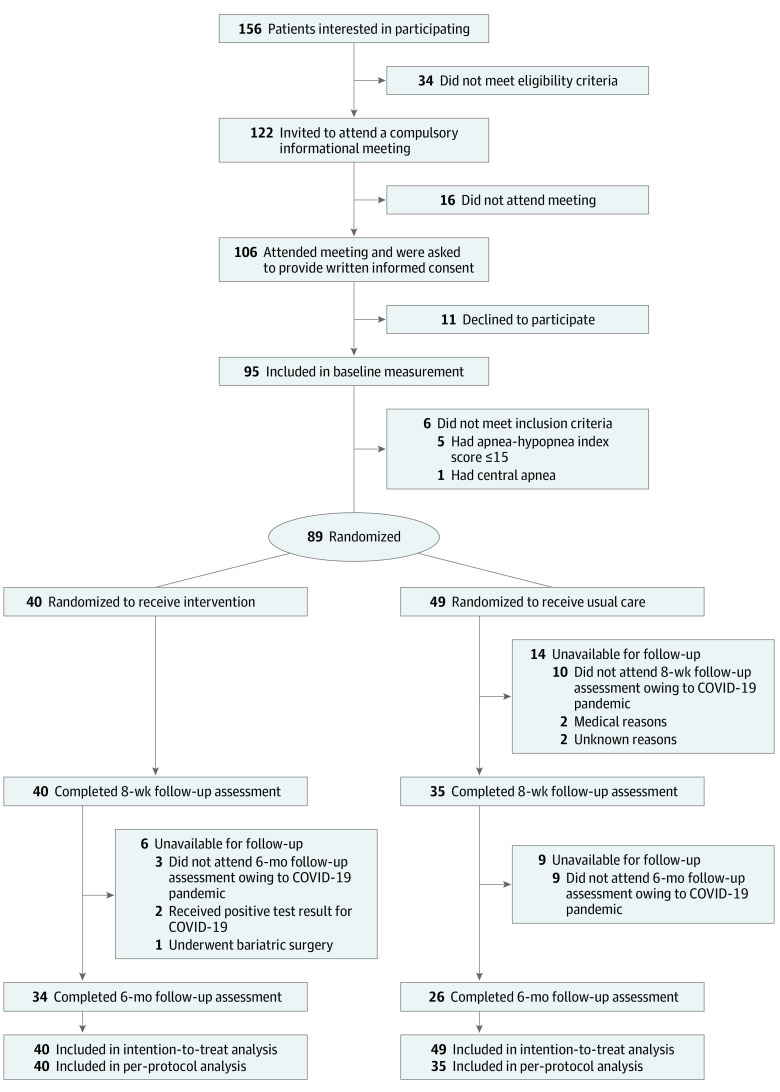
Study Flow Diagram

Among 156 men initially screened for participation, 89 were enrolled and randomized to either the control group (49 participants) or the intervention group (40 participants) (Figure 1). Overall, 14 participants (15.7%; all from the control group) were unavailable for follow-up at the intervention end point, owing mainly to the onset of the COVID-19 pandemic (10 participants). A total of 89 participants were thus included in the intention-to-treat analysis and, according to prespecified adherence criteria, 75 participants were included in the additional per-protocol analysis (information about intervention adherence is available in eMethods 4 in Supplement 2).

Among 89 men, the mean (SD) age was 54.1 (8.0) years, the mean (SD) AHI was 41.3 (22.2) events/h, and the mean (SD) BMI was 34.4 (5.4). All participants were of Spanish ethnicity. Participants had a mean (SD) walking distance of 5.6 (3.9) km/d; 24 participants (27.0%) were current smokers, and 65 participants (73.0%) reported light to moderate alcohol intake. Baseline sociodemographic and clinical characteristics were fairly well balanced between the intervention vs control group (eg, mean [SD] age, 52.6 [7.1] years vs 55.3 [8.5] years; mean [SD] AHI, 41.6 [23.5] events/h vs 41.1 [21.3] events/h; mean [SD] BMI, 35.0 [6.0] vs 33.9 [4.8]) (Table 1). Baseline characteristics were equivalent when adopting a per-protocol approach (eTable 2 in Supplement 2).

**Table 1.** Baseline Participant Characteristics

Characteristic^a^	Participants, No. (%)	
	Control group	Intervention group
Total participants, No.	49	40
Age, mean (SD), y	55.3 (8.5)	52.6 (7.1)
Educational level		
Primary education	13 (26.5)	10 (25.0)
Secondary education	10 (20.4)	6 (15.0)
Vocational education	13 (26.5)	17 (42.5)
Higher education	13 (26.5)	7 (17.5)
Marital status		
Single	7 (14.3)	2 (5.0)
Married	34 (69.4)	34 (85.0)
Divorced	8 (16.3)	4 (10.0)
Occupational status		
Employed	27 (55.1)	21 (52.5)
Self-employed	8 (16.3)	12 (30.0)
Unemployed	4 (8.2)	5 (12.5)
Retired	10 (20.4)	2 (5.0)
Medical conditions^b^		
Hypertension	33 (67.3)	27 (67.5)
Type 2 diabetes	12 (24.5)	10 (25.0)
Cardiovascular disease	9 (18.4)	7 (17.5)
Other	29 (59.2)	26 (65.0)
Medications^b^		
Antihypertensive	31 (63.3)	24 (60.0)
Statin	15 (30.6)	7 (17.5)
Oral antidiabetic	5 (10.2)	2 (5.0)
Insulin	3 (6.1)	1 (2.5)
α-Blocker	7 (14.3)	5 (12.5)
Polymedication^c^	14 (28.6)	6 (15.0)
Body height, mean (SD), cm	171 (7.9)	172 (6.3)
Body weight status		
Overweight	10 (20.4)	5 (12.5)
Obese		
Class 1	21 (42.9)	19 (47.5)
Class 2	16 (32.7)	11 (27.5)
Class 3	2 (4.1)	5 (12.5)
OSA		
Moderate	20 (40.8)	15 (37.5)
Severe	29 (59.2)	25 (62.5)
Time since OSA diagnosis, mean (SD), y	7.4 (5.7)	6.5 (6.5)
Physical activity, mean (SD), km/d	5.2 (3.9)	6.1 (3.8)
Food Behavior Checklist score, mean (SD)^d^	59.1 (9.3)	59.5 (8.5)
Alcohol consumption		
Never	11 (22.4)	13 (32.5)
Occasionally	12 (24.5)	8 (20.0)
Frequently	15 (30.6)	12 (30.0)
Daily	11 (22.4)	7 (17.5)
Tobacco consumption		
Nonsmoker	17 (34.7)	15 (37.5)
Ex-smoker	18 (36.7)	15 (37.5)
Smoker	14 (28.6)	10 (25.0)

Abbreviation: OSA, obstructive sleep apnea.

^a^ No significant between-group differences were observed in any of the baseline characteristics.

^b^ Participants could have more than 1 condition or medication.

^c^ Defined as the use of 5 or more medications.

^d^ Score range, 23-85 points, with higher scores indicating healthier dietary patterns.

### Primary and Secondary Sleep-Related End Points

Participants in the intervention group experienced a reduction in AHI from 41.6 events/h at baseline to 20.4 events/h at the intervention end point (51% reduction; change in AHI, –21.2 events/h; 95% CI, –25.4 to –16.9 events/h) and 17.8 events/h at 6 months after intervention (57% reduction; change in AHI, –23.8 events/h; 95% CI, –28.3 to –19.3 events/h). No discernible differences in AHI were observed in the control group at the intervention end point (change, 2.5 events/h; 95% CI, –2.0 to 6.9 events/h) or at 6 months after intervention (change, –0.8 events/h; 95% CI, –5.8 to 4.1 events/h). The mean difference in AHI change between groups was –23.6 events/h (95% CI, –28.7 to –18.5 events/h; *P* < .001) at the intervention end point and –23.0 events/h (95% CI, –28.4 to −17.4 events/h; *P* < .001) at 6 months after intervention (Table 2).

**Table 2.** Primary and Secondary Sleep-Related End Points

End point	Control group (n = 49)	Intervention group (n = 40)	Mean difference between groups^a^
**Primary**			
AHI, events/h (95% CI)			
Baseline	41.1 (35.3 to 46.9)	41.6 (35.1 to 48.0)	NA
Change at 8 wk	2.5 (–2.0 to 6.9)	–21.2 (–25.4 to –16.9)	–23.6 (–28.7 to –18.5)^b^
Change at 6 mo	–0.8 (–5.8 to 4.1)	–23.8 (–28.3 to –19.3)	–23.0 (–28.4 to –17.4)^b^
**Secondary**			
Oxyhemoglobin desaturation index ≥3%, events/h (95% CI)			
Baseline	45.4 (39.0 to 51.7)	45.4 (38.4 to 52.5)	NA
Change at 8 wk	3.0 (–2.7 to 8.6)	–16.0 (–21.4 to –10.7)	–19.0 (–25.4 to –12.6)^b^
Change at 6 mo	–0.8 (–7.0 to 5.5)	–23.5 (–29.2 to –17.8)	–22.7 (–29.6 to –15.7)^b^
Mean Spo_2_, % (95% CI)			
Baseline	90.3 (89.3 to 91.2)	91.3 (90.3 to 92.3)	NA
Change at 8 wk	–0.6 (–1.8 to 0.5)	1.5 (0.4 to 2.6)	2.1 (0.8 to 3.4)^c^
Change at 6 mo	–0.8 (–2.1 to 0.5)	2.6 (1.4 to 3.8)	3.4 (1.9 to 4.8)^b^
Spo_2_ nadir, % (95% CI)			
Baseline	76.8 (74.2 to 79.3)	78.1 (75.2 to 80.9)	NA
Change at 8 wk	0.3 (–1.6 to 2.2)	2.8 (1.0 to 4.6)	2.5 (0.3 to 4.7)^d^
Change at 6 mo	–1.6 (–3.7 to 0.6)	4.4 (2.5 to 6.4)	6.0 (3.6 to 8.4)^b^
Sleep time with Spo_2_ <90%, % (95% CI)			
Baseline	11.3 (8.2 to 14.3)	9.1 (5.7 to 12.5)	NA
Change at 8 wk	1.7 (–1.8 to 5.2)	–4.4 (–7.8 to –1.1)	–6.1 (–10.1 to 2.1)^e^
Change at 6 mo	1.1 (–2.7 to 5.0)	–5.5 (–9.0 to –1.9)	–6.6 (–10.9 to –2.3)^e^
Sleep efficiency, % (95% CI)			
Baseline	85.6 (83.6 to 87.7)	86.0 (83.8 to 88.3)	NA
Change at 8 wk	–1.7 (–4.9 to 1.5)	5.7 (2.5 to 8.8)	7.4 (3.7 to 11.1)^b^
Change at 6 mo	–1.6 (–5.2 to 1.9)	7.6 (4.3 to 10.9)	9.2 (5.2 to 13.2)^b^
Sleep latency, min (95% CI)			
Baseline	21.5 (17.5 to 25.6)	23.0 (18.5 to 27.5)	NA
Change at 8 wk	2.9 (–4.3 to 10.1)	–7.1 (–14.3 to 0.1)	–10.0 (–18.3 to –1.6)^f^
Change at 6 mo	4.5 (–3.3 to 12.3)	–11.2 (–18.8 to –3.7)	–15.7 (–24.6 to –6.8)^b^
Wake after sleep onset, min (95% CI)			
Baseline	54.4 (44.4 to 64.4)	47.6 (36.5 to 58.7)	NA
Change at 8 wk	11.7 (–3.8 to 27.3)	–17.2 (–32.5 to –1.9)	–28.9 (–46.8 to –11.0)^c^
Change at 6 mo	9.4 (–7.7 to 26.5)	–25.8 (–41.9 to –9.7)	–35.2 (–54.4 to –15.8)^b^
N1 plus N2 sleep, % (95% CI)			
Baseline	64.9 (62.5 to 67.3)	63.4 (60.8 to 66.0)	NA
Change at 8 wk	3.4 (–0.4 to 7.1)	–6.2 (–9.8 to –2.5)	–9.5 (–13.9 to –5.2)^b^
Change at 6 mo	4.9 (0.7 to 9.0)	–8.9 (–12.8 to –5.0)	–13.8 (–18.4 to –9.1)^b^
N3 sleep, % (95% CI)			
Baseline	20.6 (18.6 to 22.5)	20.4 (18.2 to 22.6)	NA
Change at 8 wk	–4.2 (–7.4 to –1.0)	3.7 (0.5 to 6.9)	7.9 (4.1 to 11.6)^b^
Change at 6 mo	–7.4 (–10.9 to –3.8)	4.5 (1.1 to 7.8)	11.8 (7.8 to 15.9)^b^
REM sleep, % (95% CI)			
Baseline	14.5 (13.2 to 15.8)	16.2 (14.8 to 17.7)	NA
Change at 8 wk	0.9 (–1.3 to 3.1)	2.5 (0.3 to 4.7)	1.6 (–1.0 to 4.1)
Change at 6 mo	2.7 (0.3 to 5.1)	4.5 (2.2 to 6.8)	1.8 (–0.9 to 4.5)
AHI during REM sleep, events/h (95% CI)			
Baseline	41.6 (36.0 to 47.2)	45.1 (39.0 to 51.3)	NA
Change at 8 wk	5.2 (–2.6 to 13.0)	–22.6 (–30.2 to –15.1)	–27.8 (–36.7 to –18.9)^b^
Change at 6 mo	–3.6 (–12.2 to 4.9)	–26.6 (–34.6 to –18.6)	–23.0 (–32.6 to –13.3)^b^
AHI during NREM sleep, events/h (95% CI)			
Baseline	40.6 (34.5 to 46.7)	41.0 (34.2 to 47.8)	NA
Change at 8 wk	2.2 (–2.8 to 7.1)	–21.0 (–25.7 to –16.3)	–23.2 (–28.7 to –17.6)^b^
Change at 6 mo	–1.2 (–6.6 to 4.3)	–23.5 (–28.5 to –18.6)	–22.4 (–28.5 to –16.3)^b^
Pittsburgh Sleep Quality Index, score (95% CI)^g^			
Baseline	8.8 (7.7 to 9.9)	7.2 (6.0 to 8.4)	NA
Change at 8 wk	–0.4 (–1.5 to 0.6)	–2.8 (–3.8 to –1.8)	–2.3 (–3.5 to –1.1)^b^
Change at 6 mo	0.2 (–1.0 to 1.3)	–3.6 (–4.7 to –2.5)	–3.7 (–5.0 to –2.4)^b^
Epworth Sleepiness Scale, score (95% CI)^h^			
Baseline	9.0 (7.7 to 10.3)	10.3 (8.8 to 11.7)	NA
Change at 8 wk	–0.2 (–2.0 to 1.5)	–4.6 (–6.3 to –2.9)	–4.3 (–6.3 to –2.3)^b^
Change at 6 mo	–1.0 (–2.9 to 1.0)	–6.8 (–8.6 to –5.0)	–5.8 (–8.0 to –3.7)^b^

Abbreviations: AHI, apnea-hypopnea index; N1, non–rapid eye movement stage 1; N2, non–rapid eye movement stage 2; N3, non–rapid eye movement stage 3; NA, not applicable; NREM, non–rapid eye movement; REM, rapid eye movement; Spo_2_, oxygen saturation.

^a^ Derived using the group × visit interaction term from a linear mixed-effects model that included study group, time (baseline, 8 weeks, and 6 months), and study group × time interaction term as fixed effects and participant as random effect.

^b^ *P* < .001 for time × study group interactions.

^c^ *P* = .002 for time × study group interactions.

^d^ *P* = .03 for time × study group interactions.

^e^ *P* = .003 for time × study group interactions.

^f^ *P* = .02 for time × study group interactions.

^g^ Score range, 0 to 21 points, with higher scores indicating worse sleep quality.

^h^ Score range, 0 to 24 points, with higher scores indicating more daytime sleepiness.

**Figure 2.**
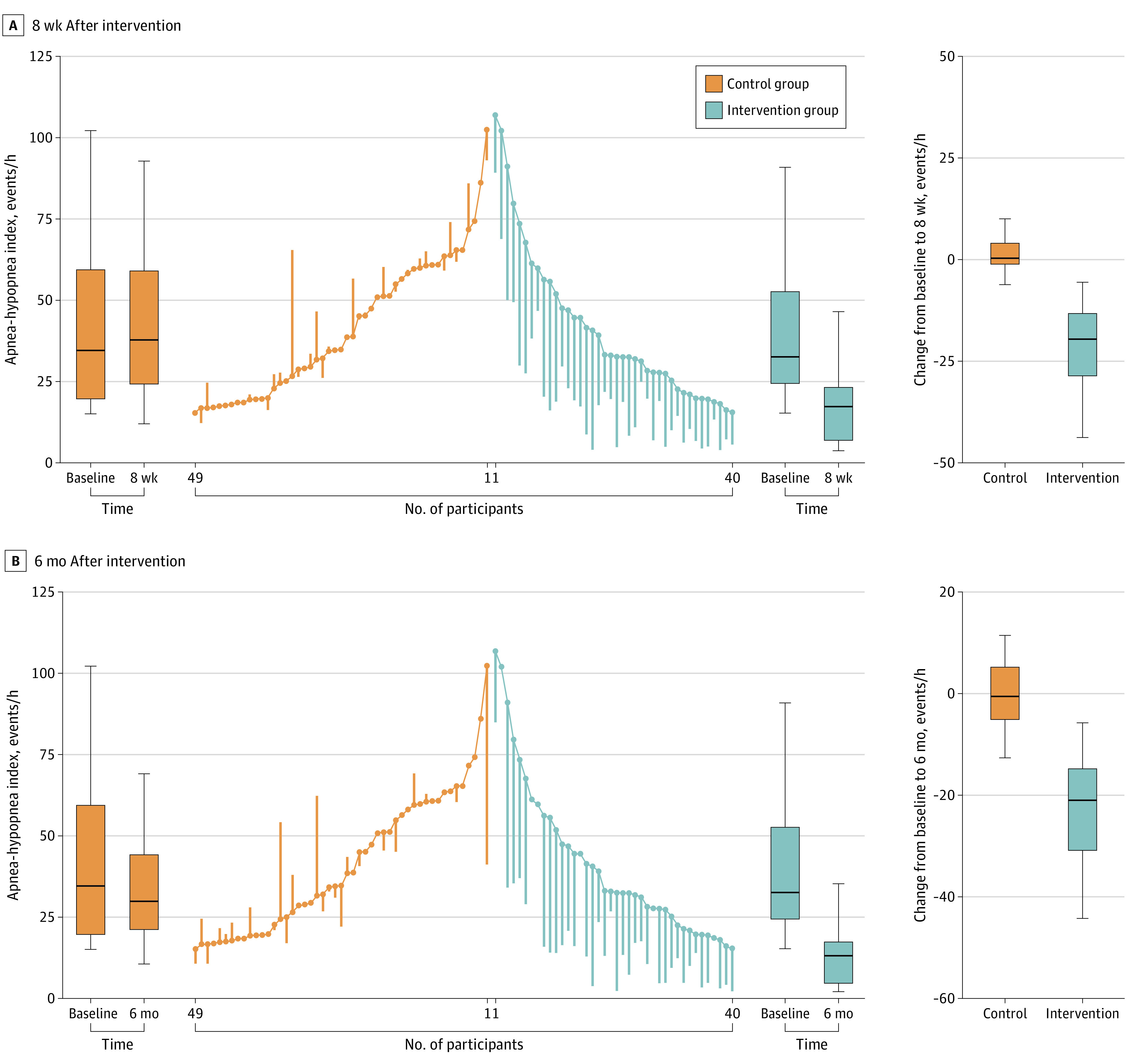
Apnea-Hypopnea Index End Point The ends of the boxes in the box plots are located at the first and third quartiles, with the black line in the middle illustrating the median. Whiskers extend to the upper and lower adjacent values, the location of the furthest point within a distance of 1.5 IQRs from the first and third quartiles. The parallel line plot contains 1 vertical line for each participant, which extends from their baseline value to their 8-week or 6-month value. Descending lines indicate an improvement in the outcome. Baseline values are placed in ascending order for the control group and descending order for the intervention group. The apnea-hypopnea index measures the number of apnea and hypopnea events per hour of sleep, with 0-4 events indicating normal (no obstructive sleep apnea [OSA]), 5-14 events indicating mild OSA, 15-30 events indicating moderate OSA, and >30 events indicating severe OSA; a change of at least 15 events is considered clinically meaningful and would move a participant 2 levels in severity status (eg, from severe to mild OSA, indicating a benefit for health).

According to AHI thresholds for OSA severity, at the intervention end point, 23 of 40 participants (57.5%) in the intervention group improved by 1 category, with 3 of 25 participants (12.0%) improving from severe to mild OSA and 6 of 40 participants (15.0%) experiencing complete remission of OSA (Figure 2; eFigure 1 in Supplement 2). At 6 months after intervention, 14 of 34 participants (41.1%) in the intervention group improved by 1 category, with 5 of 21 participants (23.8%) improving from severe to mild OSA and 10 of 34 participants (29.4%) experiencing complete remission of OSA. Notably, in the intervention group, 18 of 40 participants (45.0%) no longer required CPAP therapy at the intervention end point, and 21 of 34 participants (61.8%) no longer required CPAP therapy at 6 months after intervention; cessation of this therapy was counseled based on OSA severity reduction to a mild category and the absence of concomitant symptoms of sleepiness, impaired cognition, mood disturbance, insomnia, or other conditions, such as hypertension, ischemic heart disease, or history of stroke.

In the intervention group, similar results were observed for changes in oxyhemoglobin saturation end points (eg, oxyhemoglobin desaturation index: change at 8 weeks, −16.0 events/h [95% CI, −21.4 to −10.7 events/h]; change at 6 months, −23.5 events/h [95% CI, −29.2 to −17.8 events/h]), sleep efficiency (change at 8 weeks, 5.7% [95% CI, 2.5%-8.8%]; change at 6 months, 7.6% [95% CI, 4.3%-10.9%]), sleep maintenance (eg, wake after sleep onset: change at 8 weeks, −17.2 minutes [95% CI, −32.5 to −1.9 minutes]; change at 6 months, −25.8 minutes [95% CI, −41.9 to −9.7 minutes]), sleep architecture (eg, rapid eye movement sleep: change at 8 weeks, 2.5% [95% CI, 0.3%-4.7%]; change at 6 months, 4.5% [95% CI, 2.2%-6.8%]), subjective sleep quality (Pittsburgh Sleep Quality Index: change at 8 weeks, −2.8 points [95% CI, −3.8 to −1.8 points]; change at 6 months, −3.6 points [95% CI, −4.7 to −2.5 points]), and sleepiness (Epworth Sleepiness Scale: change at 8 weeks, −4.6 points [95% CI, −6.3 to −2.9 points]; change at 6 months, −6.8 points [95% CI, −8.6 to −5.0 points]). These results were almost identical to those obtained using the per-protocol approach (eTable 3 in Supplement 2).

With regard to changes from the intervention end point to 6 months after intervention, participants in the intervention group not only maintained improvements in all sleep-related end points but continued to experience significant improvements in oxyhemoglobin desaturation index (change, −7.4 events/h; 95% CI, −13.1 to −1.7 events/h) and sleepiness (change in Epworth Sleepiness Scale score, −2.2 points; 95% CI, −4.0 to −0.4 points) (eFigure 2 and eTable 4 in Supplement 2). Individual values at baseline, the intervention end point, and 6 months after intervention and changes in AHI (primary end point) by group are shown in Figure 2. Reductions in BMI were significantly associated with changes in AHI over time; decreases in BMI were correlated with reductions in AHI (*r* = 0.83; *P* < .001) (eFigure 3 in Supplement 2).

### Body Composition and Cardiometabolic Risk

Participants in the intervention group had greater reductions in body weight at the intervention end point (change, –7.1 kg; 95% CI, –8.6 to –5.5 kg) than participants in the control group (change, –0.3 kg; 95% CI, –1.9 to 1.4 kg), with a mean difference of –6.8 kg (95% CI, –8.7 to –4.9 kg; *P* < .001) between groups (Table 3). Similar results were found at 6 months after intervention (change, –6.9 kg [95% CI, –8.5 to 5.2 kg] in the intervention group vs –1.2 kg [95% CI, –3.0 to 0.6 km] in the control group), with a mean difference of –5.7 kg (95% CI, –7.7 to –3.6 kg; *P* < .001) between groups. Participants in the intervention vs control group had greater reductions in BMI (change at 8 weeks, −2.5 [95% CI, −3.0 to −1.9] vs −0.2 [95% CI, −0.8 to 0.4]; change at 6 months, −2.4 [95% CI, −3.0 to −1.8] vs −0.6 [95% CI, −1.2 to 0.02]), neck circumference (change at 8 weeks, −2.3 cm [95% CI, −2.8 to −1.7 cm] vs −0.3 cm [95% CI, −0.9 to 0.2 cm]; change at 6 months, −2.9 cm [95% CI, −3.5 to −2.3] vs 0.2 cm [95% CI, −0.5 to 0.8 cm]), chest circumference (change at 8 weeks, −3.4 cm [95% CI, −4.6 to −2.1 cm] vs 0.5 cm [95% CI, −0.8 to 1.8 cm]; change at 6 months, −4.1 cm [95% CI, −5.4 to −2.8 cm] vs 0.7 cm [95% CI, −0.8 to 2.1 cm]), waist circumference (change at 8 weeks, −6.9 cm [95% CI, −8.3 to −5.5 cm] vs −0.2 cm [95% CI, −0.2 to 1.3 cm]; change at 6 months, −8.8 cm [95% IC, −10.3 to −7.2 cm] vs 0.3 cm [95% CI, −1.4 to 2.0 cm]), fat mass (change at 8 weeks, −2.9 kg [95% CI, −4.5 to −1.3 kg] vs 1.4 kg [95% CI, −0.3 to 3.1 kg]; change at 6 months, −6.5 kg [95% CI, −8.2 to −4.8 kg] vs 0.2 kg [−1.7 to 2.1 kg]), and visceral adipose tissue (change at 8 weeks, −106.2 g [95% CI, −187.2 to −25.3 g] vs 32.6 g [95% CI, −52.0 to 117.1 g]; change at 6 months, −268.4 g [95% CI, −354.3 to −182.6 g] vs −26.3 g [95% CI, −119.7 to 67.1 g]). At 6 months after intervention, body weight decreased by 7%, fat mass by 19%, and visceral adipose tissue by 26% in the intervention group.

**Table 3.** Secondary Body Composition and Cardiometabolic Risk End Points

End point	Control group (n = 49)	Intervention group (n = 40)	Mean difference between groups^a^
**Body weight and composition**			
Body weight, kg (95% CI)			
Baseline	99.6 (94.5 to 104.6)	103.3 (97.6 to 108.9)	NA
Change at 8 wk	–0.3 (–1.9 to 1.4)	–7.1 (–8.6 to –5.5)	–6.8 (–8.7 to –4.9)^b^
Change at 6 mo	–1.2 (–3.0 to 0.6)	–6.9 (–8.5 to –5.2)	–5.7 (–7.7 to –3.6)^b^
BMI (95% CI)			
Baseline	33.9 (32.4 to 35.5)	35.0 (33.4 to 36.7)	NA
Change at 8 wk	–0.2 (–0.8 to 0.4)	–2.5 (–3.0 to –1.9)	–2.3 (–2.9 to –1.6)^b^
Change at 6 mo	–0.6 (–1.2 to 0.02)	–2.4 (–3.0 to –1.8)	–1.8 (–2.5 to –1.1)^b^
Neck circumference, cm (95% CI)			
Baseline	45.5 (44.4 to 46.5)	45.0 (43.9 to 46.2)	NA
Change at 8 wk	–0.3 (–0.9 to 0.2)	–2.3 (–2.8 to –1.7)	–1.9 (–2.6 to –1.3)^b^
Change at 6 mo	0.2 (–0.5 to 0.8)	–2.9 (–3.5 to –2.3)	–3.1 (–3.8 to –2.4)^b^
Chest circumference, cm (95% CI)			
Baseline	117.4 (114.5 to 120.2)	118.0 (114.9 to 121.2)	NA
Change at 8 wk	0.5 (–0.8 to 1.8)	–3.4 (–4.6 to –2.1)	–3.8 (–5.3 to –2.4)^b^
Change at 6 mo	0.7 (–0.8 to 2.1)	–4.1 (–5.4 to –2.8)	–4.8 (–6.4 to –3.2)^b^
Waist circumference, cm (95% CI)			
Baseline	117.9 (114.3 to 121.5)	119.0 (115.0 to 122.9)	NA
Change at 8 wk	–0.2 (–1.7 to 1.3)	–6.9 (–8.3 to –5.5)	–6.8 (–8.5 to –5.0)^b^
Change at 6 mo	0.3 (–1.4 to 2.0)	–8.8 (–10.3 to –7.2)	–9.0 (–10.9 to –7.2)^b^
Fat mass, kg (95% CI)			
Baseline	33.8 (31.0 to 36.7)	34.9 (31.8 to 38.0)	NA
Change at 8 wk	1.4 (–0.3 to 3.1)	–2.9 (–4.5 to –1.3)	–4.3 (–6.2 to –2.4)^b^
Change at 6 mo	0.2 (–1.7 to 2.1)	–6.5 (–8.2 to –4.8)	–6.6 (–8.7 to –4.6)^b^
Visceral adipose tissue, g (95% CI)			
Baseline	1049.2 (969.8 to 1128.5)	1017.3 (929.5 to 1105.2)	NA
Change at 8 wk	32.6 (–52.0 to 117.1)	–106.2 (–187.2 to –25.3)	–138.8 (–234.7 to –42.6)^c^
Change at 6 mo	–26.3 (–119.7 to 67.1)	–268.4 (–354.3 to –182.6)	–242.2 (–346.1 to –137.8)^b^
Lean mass, kg (95% CI)			
Baseline	60.8 (58.3 to 63.4)	63.0 (60.2 to 65.8)	NA
Change at 8 wk	–2.2 (–3.5 to –0.9)	–2.7 (–4.0 to –1.5)	–0.5 (–2.0 to 0.9)
Change at 6 mo	–1.3 (–2.7 to –0.2)	0.3 (–1.0 to 1.6)	1.6 (–0.1 to 3.2)
**Cardiometabolic risk**			
BP			
Systolic, mm Hg (95% CI)			
Baseline	142.3 (138.3 to 146.3)	143.7 (139.2 to 148.1)	NA
Change at 8 wk	–0.7 (–5.1 to 3.6)	–7.9 (–12.1 to –3.7)	–7.2 (–12.2 to –2.2)^c^
Change at 6 mo	2.6 (–2.2 to 7.4)	–13.9 (–18.3 to –9.5)	–16.5 (–21.9 to –11.1)^b^
Diastolic, mm Hg (95% CI)			
Baseline	82.1 (79.0 to 85.2)	84.0 (80.6 to 87.4)	NA
Change at 8 wk	0.2 (–4.4 to 4.8)	–5.7 (–10.3 to –1.2)	–6.0 (–11.3 to –0.6)^d^
Change at 6 mo	2.0 (–3.1 to 7.1)	–7.4 (–12.1 to –2.6)	–9.3 (–15.1 to –3.6)^e^
Mean BP, mm Hg (95% CI)			
Baseline	102.2 (99.2 to 105.2)	103.9 (100.6 to 107.2)	NA
Change at 8 wk	2.2 (–2.1 to 6.6)	–6.5 (–10.3 to –2.6)	–6.4 (–10.9 to –1.9)^c^
Change at 6 mo	2.3 (–2.2 to 6.8)	–9.6 (–13.6 to –5.5)	–11.8 (–16.6 to –6.9)^b^
Glucose metabolism			
Glucose, mg/dL (95% CI)			
Baseline	102.0 (96.0 to 107.9)	95.5 (89.1 to 102.0)	NA
Change at 8 wk	0.1 (–4.9 to 5.1)	–6.7 (–11.4 to –2.0)	–6.8 (–12.4 to –1.2)^f^
Change at 6 mo	3.6 (–1.9 to 9.0)	–6.6 (–11.6 to –1.6)	–10.2 (–16.2 to –4.1)^g^
Insulin, IU/mL (95% CI)			
Baseline	14.1 (11.9 to 16.2)	13.0 (10.7 to 15.3)	NA
Change at 8 wk	1.6 (–0.5 to 3.7)	–4.9 (–6.9 to –2.9)	–6.5 (–8.9 to –4.2)^b^
Change at 6 mo	0.3 (–2.0 to 2.5)	–5.2 (–7.3 to –3.1)	–5.4 (–8.0 to –2.9)^b^
HOMA-IR index, score (95% CI)^h^			
Baseline	3.5 (2.7 to 4.2)	3.2 (2.3 to 4.0)	NA
Change at 8 wk	0.5 (–0.6 to 1.6)	–1.3 (–2.4 to –0.3)	–1.9 (–3.1 to –0.6)^i^
Change at 6 mo	0.4 (–0.8 to 1.6)	–1.4 (–2.5 to –0.3)	–1.8 (–3.1 to –0.5)^j^
Lipid metabolism			
Total cholesterol, mg/dL (95% CI)			
Baseline	176.4 (165.8 to 187.1)	189.6 (178.1 to 201.0)	NA
Change at 8 wk	6.4 (–4.4 to 17.2)	–19.4 (–29.5 to –9.3)	–25.8 (–37.9 to –13.7)^b^
Change at 6 mo	5.7 (–6.0 to 17.4)	–16.6 (–27.3 to –5.9)	–22.3 (–35.3 to –9.3)^g^
HDL-C, mg/dL (95% CI)			
Baseline	44.7 (41.5 to 47.8)	47.1 (43.9 to 50.3)	NA
Change at 8 wk	1.9 (–0.9 to 4.8)	0.2 (–2.2 to 2.6)	–1.7 (–4.7 to 1.3)
Change at 6 mo	0.7 (–2.5 to 3.8)	3.0 (0.5 to 5.4)	2.3 (–1.0 to 5.6)
LDL-C, mg/dL (95% CI)			
Baseline	113.0 (103.7 to 122.3)	119.5 (110.1 to 128.9)	NA
Change at 8 wk	0.5 (–8.9 to 10.0)	–15.0 (–22.9 to –7.1)	–15.5 (–25.5 to –5.5)^k^
Change at 6 mo	8.2 (–2.3 to 18.7)	–14.2 (–22.4 to –6.0)	–22.4 (–33.3 to –11.6)^b^
Triglycerides, mg/dL (95% CI)			
Baseline	156.5 (136.3 to 176.7)	129.5 (107.7 to 151.2)	NA
Change at 8 wk	1.5 (–19.1 to 22.2)	–24.5 (–43.7 to –5.2)	–26.0 (–49.2 to –2.9)^d^
Change at 6 mo	8.3 (–14.0 to 30.6)	–23.7 (–44.1 to –3.3)	–32.0 (–56.9 to –7.3)^j^
Apolipoprotein A1, mg/dL (95% CI)			
Baseline	128.1 (122.4 to 133.7)	131.0 (124.9 to 137.1)	NA
Change at 8 wk	5.7 (–0.5 to 12.0)	–0.6 (–6.5 to 5.4)	–6.3 (–13.4 to 0.7)
Change at 6 mo	0.9 (–5.5 to 7.4)	9.5 (3.6 to 15.5)	8.6 (1.4 to 15.8)^f^
Apolipoprotein B, mg/dL (95% CI)			
Baseline	96.2 (89.9 to 102.6)	102.5 (95.6 to 109.3)	NA
Change at 8 wk	2.2 (–4.3 to 8.8)	–11.9 (–18.2 to –5.6)	–14.1 (–21.6 to –6.7)^b^
Change at 6 mo	–1.1 (–7.8 to 5.7)	–15.2 (–21.5 to –8.9)	–14.1 (–21.7 to –6.6)^b^
Liver function			
AST, IU/L (95% CI)			
Baseline	25.3 (22.3 to 28.3)	25.3 (22.2 to 28.4)	NA
Change at 8 wk	0.7 (–3.6 to 5.0)	–2.4 (–6.3 to 1.5)	–3.1 (–7.8 to 1.6)
Change at 6 mo	–0.7 (–5.1 to 3.6)	–4.8 (–8.7 to –0.9)	–4.0 (–8.8 to 0.7)
ALT, IU/L (95% CI)			
Baseline	28.9 (24.9 to 32.9)	29.6 (25.3 to 34.0)	NA
Change at 8 wk	0.5 (–4.6 to 5.6)	–4.0 (–8.9 to 0.8)	–4.6 (–10.4 to 1.2)
Change at 6 mo	–0.2 (–5.8 to 5.3)	–7.1 (–12.3 to –2.0)	–6.9 (–13.1 to –0.6)^b^
γ-GT, IU/L (95% CI)			
Baseline	44.1 (34.6 to 53.6)	38.2 (27.9 to 48.5)	NA
Change at 8 wk	3.7 (–4.7 to 12.0)	–11.2 (–18.9 to –3.5)	–14.9 (–24.2 to –5.6)^e^
Change at 6 mo	0.5 (–8.4 to 9.5)	–14.0 (–22.2 to –5.9)	–14.6 (–24.5 to –4.6)^i^
Fatty liver index, score (95% CI)			
Baseline	85.7 (80.5 to 90.8)	86.2 (80.6 to 91.7)	NA
Change at 8 wk	–1.5 (–6.3 to 3.3)	–13.7 (–18.0 to –9.4)	–12.2 (–17.5 to –6.9)^b^
Change at 6 mo	–0.1 (–5.1 to 5.0)	–17.5 (–22.1 to –12.9)	–17.4 (–23.0 to –11.8)^b^

Abbreviations: ALT, alanine aminotransferase; AST, aspartate aminotransferase; BMI, body mass index (calculated as weight in kilograms divided by height in meters squared); BP, blood pressure; HDL-C, high-density lipoprotein cholesterol; HOMA-IR, Homeostasis Model Assessment for Insulin Resistance; LDL-C, low-density lipoprotein cholesterol; γ-GT, γ-glutamyltransferase; NA, not applicable.

SI conversion factors: To convert ALT to μkat/L, multiply by 0.017; to convert apolipoprotein A1 to g/L, multiply by 0.01; to convert apolipoprotein B to g/L, multiply by 0.01; to convert AST to μkat/L, multiply by 0.017; to convert γ-GT to μkat/L, multiply by 0.017; to convert glucose to mmol/L, multiply by 0.05551; to convert HDL-C to mmol/L, multiply by 0.02586; to convert insulin to pmol/L, multiply by 6.945; to convert LDL-C to mmol/L, multiply by 0.02586; to convert total cholesterol to mmol/L, multiply by 0.02586; to convert triglycerides to mmol/L, multiply by 0.01129.

^a^ Derived using the group × visit interaction term from a linear mixed-effects model that included study group, time (baseline, 8 weeks, and 6 months), and study group × time interaction term as fixed effects and participant as random effect.

^b^ *P* < .001 for time × study group interactions.

^c^ *P* = .006 for time × study group interactions.

^d^ *P* = .03 for time × study group interactions.

^e^ *P* = .002 for time × study group interactions.

^f^ *P* = .02 for time × study group interactions.

^g^ *P* = .001 for time × study group interactions.

^h^ The HOMA-IR range in the study sample was 0.09 to 11.7. Values higher than 1.85 were considered to be indicators of insulin resistance in this sample of Spanish men.

^i^ *P* = .005 for time × study group interactions.

^j^ *P* = .01 for time × study group interactions.

^k^ *P* = .003 for time × study group interactions.

Greater improvements in cardiometabolic risk (including blood pressure and glucose and lipid metabolism) and liver function end points were also found in the intervention vs control group at both the intervention end point (eg, change in blood pressure, −6.5 mm Hg [95% CI, −10.3 to −2.6 mm Hg] vs 2.2 mm Hg [95% CI, −2.1 to 6.6 mm Hg]; change in γ-glutamyltransferase level, −11.2 IU/L [95% CI, −18.9 to −3.5 IU/L] vs 3.7 IU/L [95% CI, −4.7 to 12.0 IU/L]; to convert to μkat/L, multiply by 0.017) and 6 months after intervention (eg, change in blood pressure, −9.6 mm Hg [95% CI, −13.6 to −5.5 mm Hg] vs 2.3 mm Hg [95% CI, −2.2 to 6.8 mm Hg]; change in γ-glutamyltransferase level, −14.0 IU/L [95% CI, −22.2 to −5.9 IU/L] vs 0.5 IU/L [95% CI, −8.4 to 9.5 IU/L]) (Table 3). These results were almost identical to those obtained using the per-protocol approach (eTable 5 in Supplement 2). With regard to changes from the intervention end point to 6 months after intervention, benefits in body composition and cardiometabolic risk end points among the intervention group were not only sustained but significantly increased, as revealed by significant reductions in neck circumference (change, −0.7 cm; 95% CI, −1.2 to −0.1 cm), waist circumference (change, −1.8 cm; 95% CI, −3.3 to −0.3 cm), fat mass (change, −3.6 kg; 95% CI, −5.3 to −1.9 kg), and systolic blood pressure (change, −6.0 mm Hg; 95% CI, −10.4 to −1.5 mm Hg), among others (eTable 6 in Supplement 2).

### Health-Related Quality of Life and Lifestyle

Health-related quality of life in the intervention vs control group was significantly improved at the intervention end point and 6 months after intervention, as shown by changes in scores on the Sleep Apnea Quality of Life Index (change at 8 weeks, 0.8 points [95% CI, 0.5-1.1 points] vs 0.1 points [95% CI, −0.3 to 0.4 points]; change at 6 months, 1.1 points [95% CI, 0.7-1.5 points] vs 0.1 points [95% CI, −0.3 to 0.5 points]) and the 36-Item Short-Form Health Survey (eg, physical component summary score: change at 8 weeks, 6.0 points [95% CI, 2.4-9.7 points] vs 2.5 points [95% CI, −1.3 to 6.2 points]; change at 6 months, 6.5 points [95% CI, 2.6-10.3 points] vs −0.02 points [95% CI, −4.2 to 4.1 points]) (eTable 7 in Supplement 2). No significant changes in these health-related quality of life end points from the intervention end point to 6 months after intervention were found (eTable 8 in Supplement 2).

Participants in the intervention group who reported light to moderate alcohol consumption at baseline (27 of 40 individuals [67.5%]) reduced their alcohol intake to complete abstinence from the first week of the intervention to the intervention end point. At 6 months after intervention, 6 of 34 participants (17.5%) reported occasional alcohol intake (<1 drink/week), and 28 participants (87.5%) maintained complete abstinence. Seven of 10 participants (70.0%) in the intervention group who were current smokers at baseline attained complete smoking cessation at the intervention end point, and 3 of 10 participants (30.0%) reduced their tobacco consumption by 45% to 75%. At 6 months after intervention, 9 of 10 participants (90.0%) achieved and maintained complete smoking cessation.

Participants in the intervention group also had significant improvements in physical activity and dietary behaviors at both the intervention end point (change in physical activity, 8.8 km/d [95% CI, 7.3-10.4 km/d]; change in Food Behavior Checklist score, 12.0 points [95% CI, 9.5-14.5 points]) and 6 months after intervention (change in physical activity, 6.0 km/d [95% CI, 4.3-7.6 km/d]; change in Food Behavior Checklist score, 9.2 points [95% CI, 6.5-11.9 points]) (eTable 9 in Supplement 2), although slight reductions from the intervention end point to 6 months after intervention were found in both end points (change in physical activity, −2.8 km/d [95% CI, −4.5 to −1.2 km/d]; change in Food Behavior Checklist score, −2.8 points [95% CI, −5.5 to −0.1 points]) (eTable 10 in Supplement 2). No discernible differences in lifestyle habits were found among participants in the control group from baseline to the intervention end point or from baseline to 6 months after intervention.

### Adverse Events

No serious adverse events that led to death, life-threatening illness, permanent impairment, or hospitalization with serious health conditions, related or unrelated to the study intervention or participation, occurred from baseline to the intervention end point or from baseline to 6 months after intervention.

## Discussion

This randomized clinical trial showed that a novel 8-week interdisciplinary weight loss and lifestyle intervention that was carefully designed to conform to existing evidenced-based clinical practice guidelines^22,23,47,48^ led to improvement or even remission of OSA and coexisting conditions among adults with moderate to severe OSA who had overweight or obesity and were receiving CPAP therapy. Although recommended, weight loss and lifestyle interventions for OSA treatment are rarely implemented for the care of patients with this condition because of the modest quality of evidence and the methodological weaknesses present in this field of research.^22,23^

The weight loss and lifestyle intervention group had a clinically meaningful reduction in AHI of 51% at the intervention end point; 15.0% of participants attained complete remission of OSA, and 45.0% no longer required CPAP therapy. After 6 months, the reduction in AHI was 57%; complete remission of OSA was attained by 29.4% of participants, and 61.8% no longer required CPAP therapy. The intervention group notably exhibited similar reductions of 7% in body weight, 19% in fat mass, and 26% in visceral adipose tissue at 6 months after intervention. Furthermore, these results were strengthened by the evidence of significant improvement in important cardiometabolic end points involved in the pathogenesis of cardiovascular diseases. Based on reductions in systolic and diastolic blood pressure observed at the intervention end point, which were not only sustained but significantly increased at 6 months after intervention, the intervention group may have lowered their risk of stroke death by 40% and lowered their risk of death from ischemic heart disease or other vascular causes by 30%.^54^

To our knowledge, these results are representative of the best achieved using current behavioral approaches.^24^ The mechanisms by which weight loss and lifestyle changes substantially ameliorate OSA and coexisting conditions are probably multifactorial. Gathered evidence suggests that almost 60% of moderate to severe OSA is associated with obesity,^55^ which contributes to alterations of the airway anatomy and collapsibility as well as respiratory modulation.^21^ We found that reductions in BMI were significantly associated with changes in AHI over time. Adverse lifestyle behaviors, such as poor diet, low physical activity levels, smoking, and alcohol intake, have also been reported to be associated with OSA independent of body habitus.^31,32,33,56^ Thus, a combination of both weight loss and lifestyle changes may even resolve OSA in individuals with overweight or obesity.^5,23^ In addition, there is a well-recognized dose-dependent relationship between weight loss and improvement in cardiometabolic end points, with weight loss of even 5% resulting in enhanced metabolic function.^57^ Given that OSA and obesity act synergistically and have independent associations with cardiovascular risk,^21^ the beneficial effects of weight loss on cardiometabolic risk factor profiles are likely to be heightened in patients with both obesity and OSA.

### Strengths and Limitations

This study has several strengths. A major strength is the balance in the efficacy-effectiveness continuum achieved by our clinical trial, with a satisfactory internal validity accompanied by a high degree of generalizability due to our inclusion and exclusion criteria.^30^ The eligibility criteria were based on a thorough consideration of the potential generalizability of results; thus, no criteria regarding potential responsiveness, comorbidities, adherence rates, or use of nonhypnotic medications were established. Our sample therefore reflected the heterogeneity of the population with OSA. Given the study design and significant results obtained, this clinical trial may provide a clear and compelling rationale for the use of an alternative approach that is readily adaptable to real-world practice settings. Another strength is the study’s inclusion of smoking and alcohol avoidance, factors that have not been previously considered despite their recognized association with the occurrence and worsening of OSA.^31,32^

The study also has limitations. A main limitation is the sole inclusion of men in the study sample; the generalization of our findings is therefore limited to this population. The sample also included only Spanish participants; thus, our results are restricted to this ethnic population. Although this study included a 6-month follow-up assessment, the study’s duration may not have been sufficient to determine long-term intervention effects and maintenance of benefits. Due to ethical considerations, we did not include any group for whom no therapy was provided; CPAP therapy is the standard of care for moderate to severe OSA, and the inclusion of a group not receiving CPAP therapy may not be feasible.^22^

## Conclusions

In this randomized clinical trial involving Spanish men with moderate to severe OSA receiving CPAP therapy, an 8-week interdisciplinary weight loss and lifestyle intervention resulted in clinically meaningful and sustainable improvements in specific OSA-related outcomes and cardiometabolic comorbidities as well as increased health-related quality of life. Given the high prevalence of OSA, its complex and reciprocal interaction with obesity, and the fact that both conditions are readily treatable through an integrated behavioral intervention, health care professionals and policy makers might consider this approach as a central strategy to address the substantial impact of OSA on the health and welfare of populations.

## Notes

Supplement 1.
Trial Protocol

Supplement 2. **eMethods 1.** Study Organization and Eligibility Criteria **eMethods 2.** Study Assessments and End Points **eMethods 3.** Weight Loss and Lifestyle Intervention **eMethods 4.** Assessment of Adherence and Integrity of Intervention and Intervention Adherence **eTable 1.** Missing Values in Secondary Cardiometabolic Risk End Points **eTable 2.** Baseline Characteristics of Study Participants (Per-Protocol Approach) **eTable 3.** Primary and Secondary Sleep-Related End Points (Per-Protocol Approach) **eTable 4.** Primary and Secondary Sleep-Related End Points (Change From 8 Weeks to 6 Months) **eTable 5.** Secondary Body Composition and Cardiometabolic Risk End Points (Per-Protocol Approach) **eTable 6.** Secondary Body Composition and Cardiometabolic Risk End Points (Change From 8 Weeks to 6 Months) **eTable 7.** Health-Related Quality of Life End Points **eTable 8.** Health-Related Quality of Life End Points (Change From 8 Weeks to 6 Months) **eTable 9.** Physical Activity and Dietary Behavior End Points **eTable 10.** Physical Activity and Dietary Behavior End Points (Change From 8 Weeks to 6 Months) **eFigure 1.** Obstructive Sleep Apnea Severity at Baseline and 8 Weeks After Intervention in the Intervention Group **eFigure 2.** Apnea-Hypopnea Index End Point (Change From 8 Weeks to 6 Months) **eFigure 3.** Association Between Changes in Apnea-Hypopnea Index Over Time and Changes in Body Mass Index
eReferences

Supplement 3.
Data Sharing Statement
