# Supplementary material for: Effect of an Interdisciplinary Weight Loss and Lifestyle Intervention on Obstructive Sleep Apnea Severity: The INTERAPNEA Randomized Clinical Trial
Source: JAMA Netw Open. 2022 Apr 22;5(4):e228212. doi: 10.1001/jamanetworkopen.2022.8212 (PMC9034401; doi:10.1001/jamanetworkopen.2022.8212)
Supplement: Supplement 1. — Trial Protocol [file jamanetwopen-e228212-s001.pdf]

Trial Protocol and Statistical Analysis Plan

This supplement contains the following items:

1. Original study protocol.
2. Final study protocol.
3. Summary of changes to study protocol.
4. Original Statistical Analysis Plan.
5. Final Statistical Analysis Plan.
6. Summary of changes to Statistical Analysis Plan.

An investigator-initiated and conducted, randomized, controlled trial of INTERdisciplinary weight loss and lifestyle intervention for the treatment of moderate-to-severe obstructive sleep APNEA

PROTOCOL

<u>**CONTACT DETAILS**</u>

INTERAPNEA Research Team
Sleep and Health Promotion Laboratory
Mind, Brain, and Behavior Research Center (CIMCYC)
University of Granada
Campus Universitario de Cartuja, C/ del Profesor Clavera S/N,
18011 Granada, Spain
Email: acarneiro@ugr.es

CONTENTS

1. BACKGROUND AND RATIONALE 3

1.1. OSA — A MAJOR HEALTH PROBLEM 3

1.2. CPAP — FIRST-LINE TREATMENT FOR OSA 3

1.3. WEIGHT LOSS AND LIFESTYLE INTERVENTION FOR OSA 4

2. AIMS AND OBJECTIVES 4

2.1. PRIMARY AIM 4

2.2. SECONDARY AIMS 5

3. METHODS 6

3.1. DESIGN 6

3.2. ELIGIBILITY CRITERIA 6

3.3. RECRUITMENT, ENROLLMENT AND RANDOMIZATION 7

3.4. ASSESSMENTS 9

3.5. ENDPOINTS 11

3.6. WEIGHT LOSS AND LIFESTYLE INTERVENTION ARM 16

3.7. ASSESSMENT OF INTERVENTION COMPLIANCE AND INTEGRITY 24

3.8. USUAL CARE ARM 25

3.9. SERIOUS ADVERSE EVENTS 25

3.10. POWER AND SAMPLE SIZE 26

3.11. STATISTICAL ANALYSIS 26

4. ORGANIZATION 27

5. ETHICAL ISSUES 27

5.1. INSTITUTIONAL ETHICS COMMITTEE APPROVAL 27

5.2. PARTICIPANT CONSENT 28

5.3. CONFIDENCIALITY AND PRIVACY 28

5.4. PROVISION OF THE TRIAL INTERVENTION TO ALL PARTICIPANTS 28

6. REFERENCES 28

1. BACKGROUND AND RATIONALE

1.1. OSA — A Major Public Health Problem

Obstructive sleep apnea (OSA), produced by repeated upper airway collapse during sleep, has increasingly become the focus of numerous current interdisciplinary research attributed not only to its high prevalence but also to the wide range of adverse health consequences of this condition [1]. The repeated events of complete (apnea) or partial (hypopnea) pharyngeal obstruction occurred while sleeping lead to intermittent hypoxic episodes, hypercapnia, sleep fragmentation, and upsurges of sympathetic activity [2]. Driven by these short-term consequences, OSA is closely related to increased morbidity and mortality [3], including cardio-metabolic disorders [4], neurocognitive abnormalities [5], impaired daily functioning and mood [6], and greater risk of vehicle and occupational accidents [7,8].

It has recently been estimated that up to 38% of adults suffer from OSA, being more prevalent in the male sex, the elderly, and in those who are obese [9]. OSA risk factors, therefore, include obesity, sex, age, and adverse lifestyle habits such as sedentariness, poor nutrition, smoking, and alcohol intake [10]. According to epidemiological studies, nearly 60% of moderate to severe OSA is attributable to obesity [11], which contributes to alterations of the airway anatomy and collapsibility, respiratory modulation, resting lung volume, and neurohormonal mediators on ventilation [12]. Given the exponential increase of obesity prevalence in the overall population, which has nearly tripled since 1975 — 39% of adults aged 18 years and over in 2016 —, OSA prevalence is not only worryingly high but also likely to rise in upcoming years [13].

1.2. CPAP — First-Line Treatment for OSA

The current treatment of choice is continuous positive airway pressure (CPAP) [14], a mechanical device used to maintain upper airway patency, thereby improving OSA main symptoms and consequences through the reduction of the number of apnea-hypopnea episodes per hour of sleep (i.e. apnea-hypopnea index, AHI) [15-17]. However, CPAP is a chronic day-to-day treatment — it does not cure OSA in the long-term —, and its use may be rejected or abandoned due to discomfort and/or other inconveniences [18]. Most importantly, CPAP does not address the major high-risk factors of OSA, i.e. obesity and adverse lifestyles.

1.3. Weight Loss and Lifestyle Intervention for OSA

Alternative or combined behavioral interventions including weight loss through dietary approaches and exercise, sleep hygiene, and avoidance of alcohol and tobacco consumption are required and strongly recommended in the most recent practical guidelines from the American Academy of Sleep Medicine (AASM) [14,19]. According to our recently published systematic review and meta-analysis on the effectiveness of these interventions [1], the combination of diet and exercise may be an effective treatment in improving OSA outcomes in middle-aged males with moderate to severe OSA.

Yet, the number of reported randomized controlled trials addressing both weight loss components as a combination was significantly low and only included effects on specific OSA outcomes such as AHI, oxygen desaturation index, and excessive daytime sleepiness [1]. Furthermore, no original studies actively focusing on the cessation of tobacco and alcohol consumption were found [1], factors which have been shown to be common in patients with OSA and associated with the worsening of this condition [20-21]. Thus, the actual effectiveness of potential interdisciplinary interventions for the improvement of OSA main symptoms and consequences still remains unclear. Considering the vast and severe OSA consequences and comorbidities, with obesity being a major risk factor for this condition, there is a need for well-designed studies comprising all these aspects and evaluating the potential clinical and economic relevance of these interventions for OSA and related diseases.

2. AIMS AND OBJECTIVES

The interdisciplinary weight loss and lifestyle intervention for OSA (INTERAPNEA) trial aims to determine the efficacy of a novel eight-week interdisciplinary weight loss and lifestyle intervention for the improvement of OSA and comorbidities in overweight/obese adults with CPAP-treated moderate-to-severe OSA.

2.1. Primary Aim

The primary aim of the INTERAPNEA trial is to design, implement and test the efficacy of an eight-week interdisciplinary weight loss and lifestyle intervention combined with usual-care (i.e. CPAP) to improve OSA severity (i.e. reduction of AHI) in overweight/obese adults with moderate-to-severe OSA. We hypothesize that the eight-week interdisciplinary weight loss and lifestyle intervention will lead to a greater and

significant reduction of AHI and/or even remission of OSA as compared with usual-care alone (i.e. CPAP).

2.2. Secondary Aims

The secondary aims of this trial include the determination of the efficacy of the eight-week interdisciplinary weight loss and lifestyle intervention combined with usual-care (i.e. CPAP) to improve other sleep-related outcomes, body weight and composition, OSA-related coexisting conditions/comorbidities (cardiometabolic risk), health-related quality of life, and daily functioning and mood, in overweight/obese adults with moderate-to-severe OSA. Accordingly, we hypothesize that:

- Relative to usual-care alone (i.e. CPAP), an eight-week interdisciplinary weight loss and lifestyle intervention combined with usual-care will lead to a significant improvement of oxyhemoglobin saturation outcomes, sleep efficiency and maintenance, sleep architecture, and subjective sleep quality and sleepiness.
- Relative to usual-care alone (i.e. CPAP), an eight-week interdisciplinary weight loss and lifestyle intervention combined with usual-care will lead to a significant reduction of body weight and improvement of other anthropometric and body composition outcomes (neck, chest and waist circumferences, fat mass, and visceral adipose tissue).
- Relative to usual-care alone (i.e. CPAP), an eight-week interdisciplinary weight loss and lifestyle intervention combined with usual-care will lead to a significant improvement of OSA-related cardiovascular risk outcomes (hypertension, dyslipidemia, insulin resistance, and liver diseases).
- Relative to usual-care alone (i.e. CPAP), an eight-week interdisciplinary weight loss and lifestyle intervention combined with usual-care will lead to a significant improvement of health-related quality of life and overall psychological health including daily functioning and mood.

3. METHODS

3.1. Design

The INTERAPNEA study is an investigator-initiated, randomized, parallel-group, open-label trial designed to evaluate the effects of an eight-week interdisciplinary weight loss and lifestyle intervention combined with usual-care (i.e. CPAP), as compared with usual-care alone, on OSA severity (i.e. apnea-hypopnea index [AHI]; number of apneas and hypopneas per hour of sleep), and OSA-related comorbidities among adults with moderate-to-severe OSA.

3.2. Eligibility Criteria

Eligible participants will be adults previously diagnosed with moderate-to-severe OSA (AHI equal or greater than 15 [22]) from the province of Granada (Spain). They must be between 18 and 65 years old, and have a body mass index (BMI) equal to or greater than 25 kg/m^2. A full list of the study's inclusion and exclusion criteria are exposed in Table 1. Due to the well-evidenced higher incidence and prevalence of OSA in the male sex [9] and the differences in OSA phenotypes between men and women [23], we will only include male participants in the study. Furthermore, non-pharmacological and non-surgical weight loss interventions have been shown to be less effective in women [1,24], such that different approaches are needed in this population with OSA.

Table 1. Eligibility criteria.

Inclusion criteria	Exclusion criteria
• Men aged 18–65 years<br>• CPAP-treated moderate to severe OSA (AHI equal to or greater than 15 events/hour)<br>• BMI equal to or greater than 25 kg/m^2<br>• Not participating in a weight loss program<br>• Willing to provide informed consent and acceptance of random group assignment	• Presence of any other primary sleep disorder<br>• Presence of any mental disorder (including depression, anxiety, and addiction to alcohol or other substances)<br>• Presence of any other severe organic disease, except for those comorbid to OSA<br>• Regular use of neuroleptic, sedative or hypnotic drugs, or any other medication that may cause sleep disturbances or increased daytime sleepiness

AHI = apnea-hypopnea index; BMI = body mass index; CPAP = continuous positive airway pressure; OSA = obstructive sleep apnea.

Potential participants will be medically examined and must complete a health history revision prior to their inclusion in the study in order to ensure no hindrance/harm related to the assessment and intervention protocols. Should any incident or medical problem arise during the intervention, participants will be physically and psychologically examined and, if necessary, excluded from the study. Clinical trial liability insurance will be contracted for the INTERAPNEA study, providing legal and financial protection to the sponsor-investigators, and compensation to participants in the case of an injury or any damage incurred in and as a result of the study.

3.3. Recruitment, Enrollment and Randomization

Recruitment

The recruitment of participants will be performed using different strategies including enrollment from the collaborating hospital sleep unit, and use of mass media (e.g. press, magazines, radio and television news, and websites). A brief in-person or phone screening will be conducted on potentially interested participants to provide general information about the study and determine suitability of inclusion. Patients willing to participate and appearing to meet the inclusion criteria will be required to attend an in-person briefing on the rationale and study aims, inclusion and exclusion criteria, assessments to be performed, and components and characteristics of the intervention. After clarification by the research staff of any participant's doubts or questions, signatures of informed consent will be obtained from participants that meet the eligibility criteria, and appointments for the baseline assessment will be given. Participant flow from recruitment to randomization stages are shown in Figure 1.

Enrollment

Upon obtaining signed informed consents, participant demographics and medical history will be collected, and a medical/physical examination will be performed to ensure feasibility of participant inclusion in the study. Subsequently, a sleep study through a complete full-night polysomnography and other sleep measurements (daytime sleepiness, sleep quality, circadian preference, and functional outcomes of OSA) will be conducted on and taken from each participant. Furthermore, lifestyle habits such as diet, exercise and tobacco and alcohol consumption will also be measured, as well as subjective health-related quality of life, and depressive and anxiety symptoms related to OSA. After completion of participant's medical and sleep studies, objective measurements of

cardiorespiratory fitness and body composition will be taken from each participant. All test trials will therefore be performed over three different days during a one to two-week period.

Randomization

After completing baseline measurements, eligible participants will be randomly assigned to either a control group or an interdisciplinary intervention group using a computer generated simple (unrestricted) randomization [25]. Each participant will be specifically informed of which arm they have been assigned to and requested not to reveal their allocation to the research staff involved in further assessments. Bias related to unblinded participants, treatment counsellors and/or outcome assessors affecting data validity will be addressed by achieving different levels of blinding across the study personnel and participants where feasible. Therefore, study personnel responsible for data collection and analysis will be blinded to allocation assignments at the follow-ups, and blinding of participants to details of study manuals and hypothesis will be attained. When blinding is not possible, rigorous procedures of standardization of data collection and intervention, through study manuals and continuous assessment of fidelity, will be followed to avoid potential bias and ensure internal and external validity of the study [26].

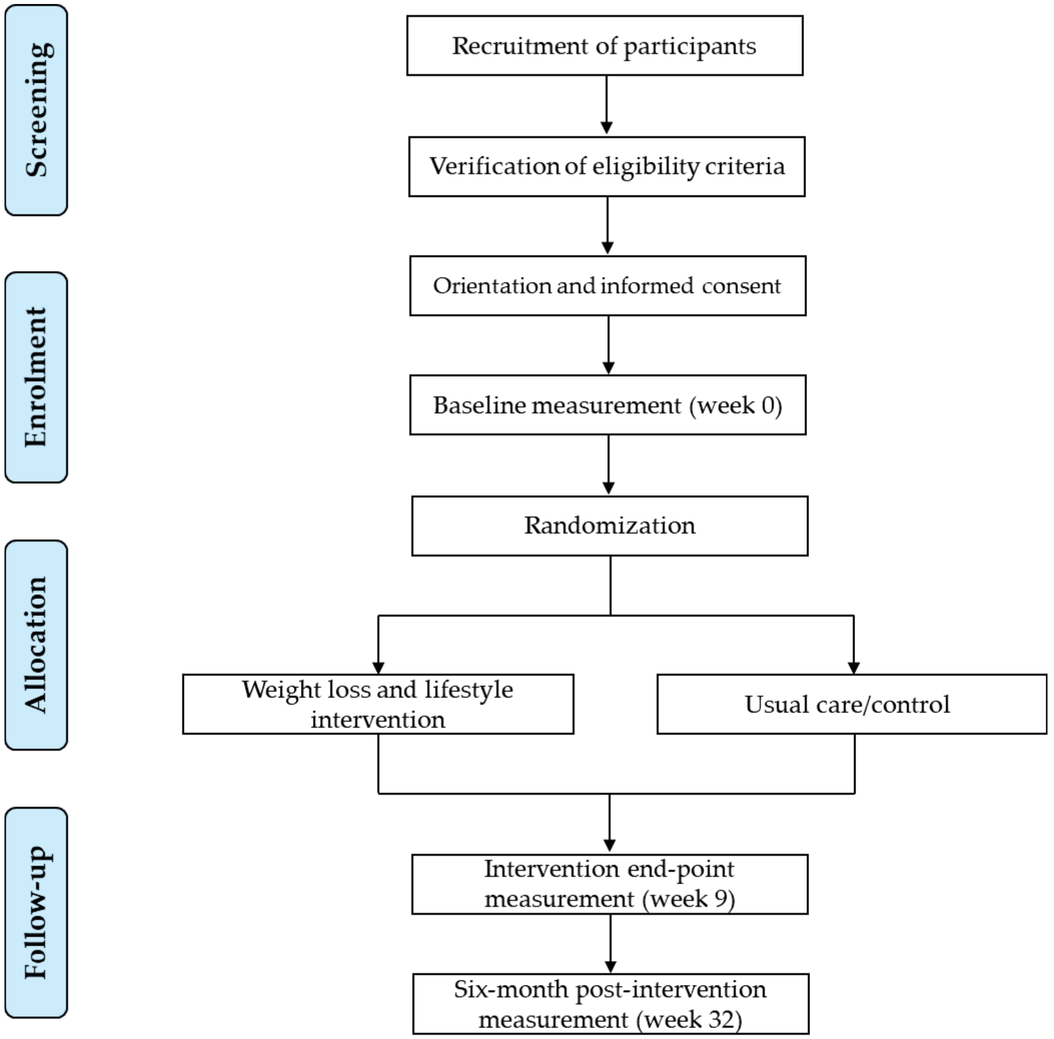

Figure 1. Flow diagram of the INTERAPNEA study participants.

3.4. Assessments

The primary outcome of INTERAPNEA study is the reduction in the number of apnea and/or hypopnea episodes per hour, i.e. AHI, assessed using a full-night ambulatory polysomnography. The main secondary outcomes include other neurophysical and cardiorespiratory polysomnographic outcomes, body weight and composition, physical fitness/cardiorespiratory fitness, and health blood biomarkers. Other variables of interest are subjective measurements of depressive and anxiety symptomatology related to OSA, impaired sleep (i.e. daytime sleepiness, sleep quality, and functional outcomes of OSA), health-related quality of life, and other lifestyle habit measurements (i.e. diet, physical activity, alcohol and tobacco consumption). All outcomes will be measured at baseline (week 0), intervention end-point (week 9), and 6 months post-intervention (week 32).

Assessment of primary and secondary outcomes will be organized and completed over three different days during a one to two-week period:

- Day 1: Potential participants will attend a medical examination and a blood test after a 12-hour overnight fast at the Sleep Unit of Virgen de las Nieves University Hospital.
- Day 2: Eligible participants will complete a full-night in-laboratory polysomnography (PSG; the gold-standard objective testing recommended by the AASM [27]), at the Sleep and Health Promotion Laboratory (CIMCYC). In order to avoid potential CPAP influence on PSG outcomes, participants will be required to withdraw from CPAP during the week prior to the PSG at baseline and follow-ups [28]. Prior to the PSG, participants will also complete a set of questionnaires assessing subjective variables related to sleep, general physical and psychological health, and lifestyle habits including diet, physical exercise, and alcohol and tobacco consumption.
- Day 3: During the third and last assessment day, participants will be required to attend the iMUDS for the measurement of anthropometric parameters, body composition and cardiorespiratory fitness.

Baseline physical activity and sleep habits will also be obtained through a seven-day self-reported daily step log and sleep diary. See Table 2 for study outcomes and measurements.

Table 2. INTERAPNEA study outcomes and measurements.

Variable	Measurement	Assessment
General health history and sociodemographic information	General medical examination (i.e. anamnesis, physical exploration, vital measurements, etc.)	Week 0
	Clinical and socio-demographic interview	Week 0, 9, 32
	Fasting blood test	Week 0, 9, 32
Sleep quality and health-related quality of life		
Sleep habits	Sleep diary	Week 0, 9, 32
Circadian preference/chronotype	Morningness/Eveningness Questionnaire	Week 0, 9, 32
Sleep quality	The Pittsburgh Sleep Quality Index	Week 0, 9, 32
Daytime sleepiness	Epworth Sleepiness Scale	Week 0, 9, 32
	Psychomotor Vigilance Test	Week 0, 9, 32
Perceived health-related quality of life	Sleep Apnea Quality of Life	Week 0, 9, 32
	Short-Form 36 Health Survey	Week 0, 9, 32
	General Health Questionnaire	Week 0, 9, 32
Objective sleep		
Neurophysiological outcomes	Polysomnography equipment	Week 0, 9, 32
Cardiorespiratory outcomes	Polysomnography equipment	Week 0, 9, 32
Body weight and composition		
BMI and anthropometric measurements	Weight and height measurement, and neck, chest and waist circumferences	Week 0, 9, 32
Body composition	Dual Energy X-ray Absorptiometry	Week 0, 9, 32
Lifestyle habits		
Physical exercise habits	Spring-levered pedometer and daily step logs	Week 0, 9, 32
Dietary habits	Food Behavior Checklist	Week 0, 9, 32
	Mediterranean Diet Adherence Screener	Week 0, 9, 32
Tobacco dependence and consumption	Self-reported tobacco consumption logs	Week 0, 9, 32
	The Fagerstrom Test for Nicotine Dependence	Week 0, 9, 32
Alcohol consumption	Self-reported alcohol consumption logs	Week 0, 9, 32
Physical fitness		
Cardiorespiratory fitness	2-km walk test	Week 0, 9, 32
Subjective physical fitness	International Fitness Scale	Week 0, 9, 32
Daily functioning and mood		
Functional outcomes related to sleepiness	Functional Outcomes of Sleep Questionnaire	Week 0, 9, 32
Subthreshold anxiety symptoms	State-Trait Anxiety Inventory	Week 0, 9, 32
Subthreshold depression symptoms	Beck Depression Inventory-Fast Screen	Week 0, 9, 32
	Inventario de Depresión Estado-Rasgo	Week 0, 9, 32

3.5. Endpoints

Primary endpoint

The primary outcome of the INTERAPNEA study is AHI, defined as the number of apnea (90% or greater drop in airflow for 10 seconds or longer) and hypopneas (30% or greater drop in airflow for 10 seconds or longer associated with ≥ 3% oxygen desaturation or an arousal) episodes per hour of sleep [29].

We will measure this outcome and other neurophysical and cardiorespiratory secondary outcomes through an in-laboratory PSG using SOMNOScreen™ PSG-Tele (SOMNOmedics, GmbH, Randersacker, Germany), or Somté PSG v2 system (Compumedics Limited, Abbotsford, Australia). The recordings will include all recommended physiologic signals such as electroencephalogram (three channels: F4-M1, C4-M1, O2-M1), electrooculogram (two channels: E1 and E2), electromyogram (two channels: submental and anterior tibialis muscles), and electrocardiogram (two channels). Cardiorespiratory measurements will include oral and nasal airflow (triple thermistor), oxyhaemoglobin saturation (SpO2) and pulse-rate (pulse oximeter), respiratory effort (chest and abdomen bands), and body position (sensor). All electrodes will be placed in accordance with the international 10-20 system [30], and recordings will be automatically and manually scored in 30-second epochs [31] by trained physicians using DOMINO (v2.7, SOMNOmedics, GmbH, Randersacker, Germany), or ProFusion PSG 3 (v3.3, Compumedics Limited, Abbotsford, Australia) associated computer software. All parameters, settings, filters, technical specifications, sleep stage scoring and event scoring will be performed in accordance with the AASM Manual for the Scoring of Sleep and Associated Events [29].

We will also specifically analyze AHI in rapid eye movement (REM) and non-REM sleep stages (N1, N2, and N3). Although it has been shown that REM apnea episodes may yield to more adverse cardiovascular consequences than non-REM obstructions [32], previous similar RCTs have rarely included the reduction in AHI differentiated by these sleep stages [1].

Secondary Endpoints

Neurophysical and cardiorespiratory polysomnographic outcomes

Secondary polysomnographic outcomes related to OSA, measured by PSG as above mentioned, are oxygen desaturation index (number of oxygen desaturation $\geq$ 3% per hour), SpO2 mean (average of oxygen saturation), SpO2 nadir (minimum oxygen saturation), sleep efficiency (total sleep time/total time in bed), sleep latency, wake after sleep onset, REM sleep stage, and nonREM sleep stages (N1, N2, and N3).

Physical fitness

Cardiorespiratory fitness will be measured through a 2-km walking test, which has been widely used and validated for accurate estimation of maximum oxygen uptake

(VO_{2max}) [33]. Participants will be required to walk over a marked 2 km track on a firm surface wearing a heart rate monitor (Polar RS800cx, Polar Electro, Kempele, Finland). Walking time and heart rate (HR) will be recorded at the end of the test. The maximal aerobic power will then be calculated considering age, BMI, performance time, and HR with the following formula VO_{2max} (ml/min/kg) = 116.2 - 2.98 * walking time (sec) - 0.11 * HR -0.14 * age - 0.39 * BMI [34]. Participant's scores will be obtained and placed within a fitness category. Subjective physical fitness will also be measured using the International Fitness Scale (IFIS) [35].

Body weight and composition

Body weight and height will be measured using a calibrated scale and stadiometer (model 799, Electronic Column Scale, Hamburg, Germany) with participants wearing undergarments. Neck, chest and waist circumferences will also be measured following standard procedures recommended by the International Society for the Advancement of Kinanthropometry (ISAK) [36]. Body composition measurements including fat mass (kg), fat free mass, lean mass (kg), visceral adipose tissue (kg), and bone mineral density (g/cm^2) will be obtained through a full-body dual energy X-ray absorptiometry (DXA) scanner (Discovery Wi, Hologic, Inc., Bedford, MA, USA). Quality controls, positioning of participants and analyses of results will be performed following the manufacturer's recommendations. Automatic delineation of anatomic regions will be performed using APEX 4.0.2. software.

Blood biomarkers

Blood biomarkers will include glucose metabolism (glucose [mg/dl], insulin [IU/ml] and insulin resistance as indicated by the homeostasis model assessment of insulin resistance [HOMA-IR] index), lipid metabolism (total cholesterol [mg/dl], high-density lipoprotein cholesterol [HDL-C; mg/dl], low-density lipoprotein cholesterol [LDL-C; mg/dl], triglycerides [mg/dl], and apolipoproteins A1 and B [mg/dl]), and liver function (aspartate aminotransferase [AST; IU/l], alanine aminotransferase [ALT; IU/l], γ-glutamyltransferase [γ-GT], and fatty liver index [FLI]). These variables will be measured through blood samples obtained from participants' antecubital vein in a supine position during the morning in a fasting state. Samples will be collected into prechilled ethylene diamine tetra-acetic acid-containing tubes (Vacutainer SST, Becton Dickinson, Plymouth, UK) and immediately centrifuged (i.e. 15 minutes at 3,000 rpm), aliquoted and

stored at −80°C for further plasma analysis. Glucose levels will be measured by spectrophotometric techniques (AU5800, Beckman Coulter, Brea, California, USA). Insulin will be assessed by chemiluminescence immunoassay with paramagnetic particles (UniCel DxI 800, Beckman Coulter, Brea, California, USA). HOMA-IR will be calculated as fasting glucose (mmol/l) times the level of fasting insulin (UU/ml) divided by 22.5. Total cholesterol, HDL-C, triglycerides, and apolipoproteins A1 and B will be automatically evaluated by spectrophotometric techniques (AU5800, Beckman Coulter, Brea, California, USA). LDL-C will be calculated as the level of total cholesterol minus the level of HDL-C minus 0.45 times the level of triglycerides. AST, ALT and γ-GT will be calculated by absorption spectrophotometric techniques (Beckman Coulter, Brea, California, USA). FLI will be calculated with the formula FLI = ((e 0.953·loge (Triglycerides) + 0.139·BMI + 0.718·loge (γ-GT) + 0.053·Waist Circumference - 15.745)) / ((1 + e 0.953·loge (Triglycerides) + 0.139·BMI + 0.718·loge (γ-GT) + 0.053·Waist Circumference - 15.745)) ·100.

Systolic blood pressure (mm Hg), diastolic blood pressure (mm Hg) and mean blood pressure (mm Hg) will also be considered as cardiometabolic risk outcomes. Blood pressure will be measured with an ambulatory blood pressure monitor (Omron M3 Blood Pressure Monitor, OMRON Healthcare, Hoofddorp, Netherlands) in a sitting position after at least 5 minutes of rest. The mean of two measurements will be recorded. Mean blood pressure will be calculated at each reading as one third of systolic pressure plus two thirds of diastolic pressure.

Lifestyle habits: Dietary habits, physical activity, smoking, and alcohol intake

Participants' dietary habits will be evaluated using the validated 14-item Mediterranean diet screener (MEDAS), which evaluates food consumption frequency (12 items) and characteristic dietary habits of the Mediterranean diet (2 items) [37]. MEDAS items are scored with 0 or 1, the total score ranging from 0 to 14 points. The 22-item Food Behavior Checklist (FBC) will also be used to assess participants' food intake and habits [38]. FBC comprises seven subscales including consumption of fruit and vegetables (9 items), diet quality (4 items), fast food (3 items), dairy/calcium (2 items), sweetened beverages (2 items), meat (1 item) and food security (1 item). This instrument has been shown to be effective at evaluating dietary behavior changes after nutrition education interventions promoting healthy diets [38].

Physical activity will be measured using daily step logs recorded by participants with a spring-levered pedometer (OcioDual, Alicante, Spain). Participants will be required to wear the pedometer all day and register the number of steps achieved per day in a seven-day step log. The average steps per day will then be calculated at baseline and follow-ups.

Regarding the remaining lifestyle habits, smoking and alcohol intake will be measured at baseline and follow-ups using seven-day self-reported tobacco and alcohol consumption logs. Recordings will include number of cigarettes/alcoholic units consumed per day, cigarette brand/type of alcoholic drink, time, situation, and perceived pleasure (from 0 to 10). The validated form of the Fagerström Test for Nicotine Dependence (FTND) [39] will also be used to assess participants' nicotine dependence in all assessments.

Daily functioning and mood

OSA impact on daily functioning and mood will be measured through validated versions of the Functional Outcomes of Sleep Questionnaire (FOSQ) [40], Beck Depression Inventory-Fast Screen (BDI-FS) [41], State-Trait Anxiety Inventory (STAI) [42] and Inventario de Depresión Estado-Rasgo (IDER) [43]. It has been shown that impaired daytime functioning and depressive and anxiety symptoms are very common in patients with OSA, being higher in patients with more severe OSA and a greater BMI [44]. Hence, participants will complete a set of questionnaires on these symptoms not only to measure inclusion/exclusion criteria but also to analyze potential changes in daily functioning and mood driven by the INTERAPNEA study intervention.

Daytime sleepiness, sleep quality and health-related quality of life

The Epworth Sleepiness Scale (ESS) [45], an 8-item Likert-based scale, will be used to obtain subjective measurements of participant's daytime sleepiness. Excessive daytime sleepiness is the most common consequence of OSA due to sleep fragmentation and deprivation, and one of the mediating factors for other OSA outcomes such as daily functioning, social and occupational disturbances [6-8]. We will also include the Psychomotor Vigilance Test (PVT) [46], regarded as a potential and reliable objective measure of sleepiness, using PC-PVT v 2.0 software (Biotechnology HPC Software Applications Institute [BHSAI], https://pcpvt.bhsai.org/pcpvt/register.xhtml, Frederick, Maryland, USA) on a personal computer [47,48]. This 10-minute sustained attention task

consists of responding to visual stimulus randomly presented in a black screen each 2 to 10 seconds, to which participants have to respond by clicking the mouse, with the reaction time being registered and analyzed in terms of response speed and number of lapses (reaction time > 500 milliseconds).

As sleep quality is also closely related to daily functioning, mood and, thus, participant's general quality of life and well-being [49,50], we will measure potential benefits of the INTERAPNEA study intervention on these variables through the validated versions of the Pittsburgh Sleep Quality Index (PSQI) [51], Sleep Apnea Quality of Life (SAQLI) [52], Short-Form 36 Health Survey (SF-36) [53], and General Health Questionnaire (GHQ-28) [54].

In addition, we will subjectively measure circadian preference or individual's chronotype using the validated reduced 5-item version of the Morningness-Eveningness Questionnaire (MEQ) [55], an outcome which has been closely related to age, BMI and, in turn, OSA [56]. Evidence suggests that evening-type chronotype may be highly associated with greater unhealthy eating behaviors, sleep disruption, poor sleep quality and mood disturbances, all playing a part in the development and severity of OSA [56,57].

3.6. Weight Loss and Lifestyle Intervention Arm

The design, implementation and evaluation of the INTERAPNEA study intervention components and characteristics are based on results of previous epidemiological and clinical research [1,10] as well as on international evidenced-based clinical practice guidelines for the management of OSA [14,19]. Considering our previous research [1] and with the final aim of the intervention being adaptable to actual primary health-care settings, the intervention will last eight weeks, and will be composed of five different modules (i) nutritional behavior change, (ii) moderate aerobic exercise, (iii) smoking reduction and cessation, (iv) alcohol intake avoidance, and (v) sleep hygiene (see Table 3). Each component will include group-based weekly sessions of 60-90 minutes lead and supervised by a trained professional in the field (i.e. human nutrition and dietetics, physical activity and sport sciences, and psychology).

The key-factor of this interdisciplinary intervention will be the use of the Transtheoretical Model of Health Behavior Change (TM) by Prochaska and Diclemente [58]. This well-evidenced model of behavior change is based on integrating different intervention theories into an interventional approach that considers different stages, processes and principles of change with the premise of establishing sustainable health-

related behaviors or habits [58]. Consciousness raising, self-reevaluation, counterconditioning, stimulus control, contingency management, goal-setting, self-monitoring, self-efficacy, and decisional balance are some of the processes and principles of change addressed by this theory and, therefore, included in the five different INTERAPNEA intervention components. Physical and dietary interventions for weight loss using strategies of TM and, thus, psychological support, have been shown to be more effective than other approaches in overweight and obese patients [59] and, specifically, in those with OSA [1].

Table 3. Description and timing of the INTERAPNEA intervention modules and components.

<table>
<tr><th>Module</th><th>Objectives/Description</th><th>Number of sessions</th><th>Frequency of sessions</th><th>General behavioral change techniques</th></tr>
<tr><td>Nutritional behavior change</td><td>Nutrition education and dietary patterns change</td><td>8</td><td>Once a week</td><td rowspan="5">• Motivation and preparation for action
• Goal-setting and action-planning
• Self-monitoring and functional behavioral analysis
• Review of behavioral goals, action plans, and adherence
• Problem solving and social skills
• Self-efficacy, maintenance, and relapse prevention</td></tr>
<tr><td>Physical exercise</td><td>Supervised moderate aerobic exercise and increase daily steps by 15% each week</td><td>8</td><td>Once a week</td></tr>
<tr><td>Sleep hygiene</td><td>Change of inappropriate sleep habits: insufficient sleep, consumption of coffee, alcohol and tobacco, and inappropriate sleep schedule and environment</td><td>4</td><td>Once each two weeks</td></tr>
<tr><td>Tobacco cessation</td><td>Nicotine and cigarette fading: reduction of nicotine and number of cigarettes by 30% each week</td><td>8</td><td>Once a week</td></tr>
<tr><td>Alcohol avoidance</td><td>Alcohol consumption fading: reduction of alcohol consumption by 30% each week</td><td>4</td><td>Once each two weeks</td></tr>
</table>

Nutritional behavior change

Diet quality and dietary patterns have been shown to be closely related to sleep disruption, fragmentation and poor sleep quality found in OSA [60]. Recent studies have shown that high-fat intake is associated with lower sleep efficiency and REM sleep and higher arousal index, whereas high-carbohydrate intake may improve sleep duration and architecture by producing reductions in sleep-onset latency and higher proportions of REM sleep [60]. Regarding intake of micronutrients, vitamin D — which has been associated with insulin resistance in OSA [61] — and magnesium deficiencies have also been related to shorter sleep duration, poorer sleep quality and higher daytime sleepiness

[62,63]. Therefore, dietary components including milk, fish, fruit and vegetables may yield beneficial effects on sleep and, in turn, OSA [60].

Furthermore, evidence suggests that sleep disturbances occurred in OSA, in turn, have adverse consequences on calorie intake and expenditure, exposing therefore a two-way relationship between dietary habits and sleep [64]. Empirical studies have revealed that sleep fragmentation and deprivation are related to higher energy intake of unhealthy food due to increased hunger, food craving, food reward and portion size selection [65-66]. Neurocognitive impairments found in patients with OSA such as attention and episodic memory deficits [67] have also been associated with higher intake of saturated fats, loss of control over food intake, and thus uncontrolled eating [68].

Hence, the INTERAPNEA intervention includes a nutrition module comprising eight sessions (once a week) of 60-90 minutes in a group format addressing dietary patterns using integrated techniques of nutrition education and behavioral change such as specific goal-setting, cognitive restructuring, stimulus control, progressive muscle relaxation, social skills and assertiveness, and problem solving skills. The nutrition education is based on the World Health Organization (WHO) latest recommendations on food intake and healthy diet (see Table 4 for detailed topics) and each session will follow a three-part format: i) Brief review of previous session and participant's adherence to recommendations; ii) Development of the main nutrition education component of each session using an interactive group discussion layout; iii) Resolution of participant's questions and/or concerns, and setting of specific goals. No specific or individualized diet will be indicated to participants.

Table 4. Description of nutrition education per session.

Session	Nutrition education topics
Session 1	Adverse consequences of obesity, importance of healthy nutrition and body composition on health, and positive effects of changes in nutrition.
Session 2	Maintenance of a healthy nutrition based on the Harvard Plate model: increasing consumption of healthy food (vegetables, fruits, legumes, nuts, extra olive virgin oil, fish and shellfish, white meat, eggs and herbs) and decreasing consumption of unhealthy food (ultra-processed foods, excessive salt consumption, processed meats, red meat, alcohol, and high-calorie foods and beverages).
Session 3	Food myths and health risks of miracle diets.
Session 4	Strategies to improve satiety and decrease appetite: decreasing dishes dietary energy density, choosing food with low dietary energy density, managing dietary fat intake, including enough fiber and protein quantity, limiting sugar and ultra-processed foods, choosing water and low-calorie beverages, and managing portion sizes.
Session 5	Healthy breakfast and snacks: avoiding unhealthy breakfast and snacks and turning them into healthy.
Session 6	Healthy cooking, food purchase and choices when eating out.
Session 7	How to read nutritional labels of food and distinguish between healthy and unhealthy food.
Session 8	Nutritional strategies to improve sleep quality.

Physical exercise

Physical exercise has been shown to be effective in enhancing OSA outcomes and health-related consequences [1,69,70]. Due to the close association between OSA and obesity, a significant and sustainable increase of physical activity could lead to a reduced body weight and, in turn, improvement of the upper airway structure, function, and resting lung volume [12]. Furthermore, physical exercise could also assist the balance of energy intake and expenditure [71], and improve respiratory center modulation through a reduction of the high leptin and ghrelin hormone levels, abnormalities linked to excessive energy intake and found in OSA patients [72]. Yet, some research found that exercise benefits on OSA were independent to weight loss [70], suggesting that there are other related mechanisms potentially leading to OSA enhancement such as the increase of sleep efficiency and slow wave sleep [73], and a decrease of fluid accumulation implicated in the upper airway collapse [74], both due to the direct association between physical exercise and sleep.

Therefore, the INTERAPNEA study will include an eight-week physical exercise program consisting of weekly 60 min-sessions of supervised moderate instensity aerobic exercise (i.e. 55-65% of the heart rate reserve) and individualized goal-setting of increasing daily steps per week. Previous studies have emphasized that walking may be the exercise modality achieving higher levels of weight loss and increased

cardiorespiratory fitness in adults with obesity and CPAP-treated OSA [75]. Thus, in the weekly supervised training sessions, participants will be required to walk at a moderate intensity for 60 minutes wearing a heart rate monitor in order to train themselves to walk at that intensity during the rest of the week. With respect to goal-setting, they will be advised to increase their daily steps by 15% per week, based on their daily steps logs.

Sleep hygiene

Sleep hygiene refers to the practice of certain behaviors that facilitate sleep onset and maintenance (e.g. regular sleep schedule, appropriate sleep environment, exercise-training, and healthy nutrition), and avoidance of habits interfering with sleep (e.g. daytime napping, smoking, alcohol intake, and use of hypnotics) [76]. Patients with OSA frequently exhibit poor sleep hygiene including voluntary sleep restriction, irregular sleep schedule, inappropriate sleep environment, and excessive consumption of alcohol, nicotine and/or caffeine [77]. Accordingly, previous studies have supported the inclusion of this component on the treatment of OSA as effective in improving sleep quantity and efficiency, and therefore daytime sleepiness [1,78,79].

The INTERAPNEA study intervention will include a sleep hygiene module comprising 60 min sessions supervised by a psychologist specialized in the evaluation and treatment of sleep disorders. As most sleep hygiene topics will be covered in simultaneous modules, there will be four sessions distributed over the eight weeks of the intervention, consisting of sleep hygiene education on causes of sleep disturbances and mistaken sleep related knowledge (see Table 5). Sessions will also be based on treating those frequent inadequate sleep habits found in patients with OSA, i.e. sleep restriction, irregular schedule and inappropriate sleep environment.

Table 5. Summary of components of the sleep hygiene module per session.

Session	Intervention objectives/components
Session 1	• Goal-setting and action-planning: Objective specification and commitment<br>• Self-monitoring: Sleep diary<br>• Psychoeducation: What is sleep hygiene?<br>• Cognitive restructuring: Irrational, false or inaccurate beliefs about sleep<br>• Breathing and relaxation techniques: Diaphragmatic breathing and progressive muscle relaxation
Session 2	• Review of behavioral goals, action plans, and compliance (participant's homework)<br>• Self-monitoring: Sleep diary<br>• Psychoeducation: Voluntary sleep restriction; coffee, alcohol and tobacco consumption before sleep; and irregular sleep schedule and environment.<br>• Cognitive restructuring: Irrational, false or inaccurate beliefs about sleep<br>• Review of diaphragmatic breathing and progressive muscle relaxation
Session 3	• Review of behavioral goals, action plans, and compliance (participant's homework)<br>• Self-monitoring: Sleep diary<br>• Vicarious and self-reinforcement: Changes in sleep habits achieved and benefits<br>• Stimulus control and bedtime restriction<br>• Review of diaphragmatic breathing and progressive muscle relaxation
Session 4	• Review of behavioral goals, action plans, and compliance (participant's homework)<br>• Self-monitoring: Sleep diary<br>• Vicarious and self-reinforcement: Changes in sleep habits achieved and benefits<br>• Review of all intervention components: Main factors of sleep hygiene, diaphragmatic breathing and progressive muscle relaxation, stimulus control and bedtime restriction<br>• Maintenance and relapse prevention: Analysis of high risk situations for unhygienic sleep.

Reduction and avoidance of tobacco consumption

Smoking has been related to the worsening of OSA via different mechanisms such as changes in sleep architecture and increase of arousal threshold from sleep, reduction of the upper airway muscle tones and neural reflexes, and increased inflammation of the upper airway, all due to nicotine and smoke inhalation [21]. In turn, OSA could also be a predisposing factor for smoking addiction, nicotine acting as a reward or self-medication related to the depressive and anxiety symptoms commonly found in OSA [80]. Although this association has been well elucidated, there are no empirical evidences of the potential beneficial effects of smoking cessation on OSA as, surpringsily, there are no studies focusing on active smoking cessation interventions in patients with this condition [1].

Therefore, we will include a smoking reduction and avoidance module in the INTERAPNEA study intervention. Participants with smoking addiction and willing to quit will be required to attend a weekly 60-90 minute session over eight weeks lead by two clinical psychologists. The specific intervention is based on the group behavior therapy for smoking cessation by Becoña et al. [81]. This therapy seeks the progressive reduction of tobacco consumption through the use of nicotine and cigarette fading [82], and behavior-change techniques such as information on smoking, self-monitoring,

stimulus control, avoidance of withdrawal symptoms, and relapse prevention (see Table 6). Nicotine and cigarette fading has been shown to be the most effective method to reduce and stop smoking with abstinence rates of 86% at the end of treatment and nearly 60% at a 12 month follow-up [83].

Thus, participants will be mainly required to keep a daily record of number of cigarettes smoked and triggers for smoking (self-monitoring), change the type of cigarette smoked to a lesser nicotine content brand each week (30%, 60% and 90% nicotine reductions from baseline), reduce the number of cigarettes smoked by 30% weekly, and avoid smoking in three different situations per week (stimulus control). Through the sessions, other behavior change techniques will be used such as discussion of health consequences of smoking and quitting (motivation), muscle and cognitive relaxation techniques to address withdrawal symptoms, and identification of high-risk situations for smoking and problem-solving skills (relapse prevention).

Table 6. Summary of components of the smoking cessation module per session.

Session	Intervention objectives/components
Session 1	• Goal-setting and action-planning: Objective specification and commitment<br>• Self-monitoring: Cigarette consumption logs<br>• Psychoeducation: Cigarette components and smoking consequences<br>• Cognitive restructuring: Irrational, false or inaccurate beliefs about smoking cessation<br>• Stimulus control: Reduction strategies<br>• Nicotine and cigarette fading: Reduction of nicotine/number of cigarettes by 30% from baseline<br>• Breathing and relaxation techniques: Diaphragmatic breathing and progressive muscle relaxation
Session 2	• Review of behavioral goals, action plans, and compliance (participant's homework)<br>• Self-monitoring: Cigarette consumption logs<br>• Cognitive restructuring: Irrational, false or inaccurate beliefs about smoking cessation<br>• Stimulus control: Smoking avoidance in three different situations and other reduction strategies<br>• Nicotine and cigarette fading: Reduction of nicotine/number of cigarettes by 30% from week 1<br>• Maintenance and relapse prevention: Avoidance of withdrawal symptoms<br>• Review of diaphragmatic breathing and progressive muscle relaxation
Session 3	• Review of behavioral goals, action plans, and compliance (participant's homework)<br>• Self-monitoring: Cigarette consumption logs<br>• Vicarious and self-reinforcement: Changes in smoking achieved and benefits<br>• Stimulus control: Smoking avoidance in six different situations and other reduction strategies<br>• Nicotine and cigarette fading: Reduction of nicotine/number of cigarettes by 30% from week 2<br>• Maintenance and relapse prevention: Avoidance of withdrawal symptoms<br>• Review of diaphragmatic breathing and progressive muscle relaxation
Session 4	• Review of behavioral goals, action plans, and compliance (participant's homework)<br>• Self-monitoring: Cigarette consumption logs<br>• Vicarious and self-reinforcement: Changes in smoking achieved and benefits<br>• Stimulus control: Smoking avoidance in nine different situations and other reduction strategies<br>• Nicotine and cigarette fading: Reduction of nicotine/number of cigarettes by 30% from week 3<br>• Maintenance and relapse prevention: Avoidance of withdrawal symptoms<br>• Review of diaphragmatic breathing and progressive muscle relaxation

Session 5	• Review of behavioral goals, action plans, and compliance (participant's homework)<br>• Self-monitoring: Cigarette consumption logs<br>• Vicarious and self-reinforcement: Changes in smoking achieved and benefits<br>• Abstinence planning: Setting the day when abstinence starts<br>• Problem solving and social skills: High risk situations for smoking and alternative behaviors<br>• Maintenance and relapse prevention: Avoidance of withdrawal symptoms<br>• Review of diaphragmatic breathing and progressive muscle relaxation
Sessions 6, 7, 8	• Review of behavioral goals, action plans, and compliance (participant's homework)<br>• Self-monitoring: Cigarette consumption logs/Review of abstinence<br>• Abstinence planning: Setting the day when abstinence starts<br>• Vicarious and self-reinforcement: Changes in smoking achieved and benefits<br>• Problem solving and social skills: High risk situations for smoking and alternative behaviors<br>• Review of diaphragmatic breathing and progressive muscle relaxation<br>• Maintenance and relapse prevention: Difference between lapse and relapse

Reduction and avoidance of alcohol intake

Alcohol intake has also been related to the development and worsening of OSA not only for its direct and indirect effects on weight gain but also due to its negative impact on breathing parameters during sleep [20]. Recent meta-analyses on alcohol and risk of sleep apnea emphasized that alcohol intake increases the risk of breathing cessation episodes by 25%, thus increasing AHI and reducing mean SaO2 during sleep [84]. Potential explanations of these adverse consequences may be the alcohol-related hypotonia of oropharyngeal muscles during sleep, and depression of the arousal response to asphyxia, both caused by the alcohol depressant effects on the central nervous system [85].

Therefore, the INTERAPNEA study intervention will include an alcohol intake reduction and avoidance module supervised by two clinical psychologists. As we will be treating excessive alcohol intake as opposed to alcohol dependence, this module will last eight weeks comprising fortnightly sessions of 60 minutes. Similar to the smoking cessation module, the main content of this specific component is the progressive reduction of alcohol intake in those participants with no alcohol addiction but excessive consumption (see Table 7). Thus, participants will be indicated to reduce the number of units of alcohol consumed per day/week by 30% each week, keeping a log of alcohol-consumption per day including units of alcohol consumed and triggers of consumption. During the sessions, participants will receive detailed information of alcohol general and specific to OSA health-related consequences. Furthermore, behavior change techniques such as stimulus control, muscle and cognitive relaxation and problem-solving skills related to alcohol consumption will be used.

Table 7. Summary of components of the alcohol avoidance module per session.

Session	Intervention objectives/components
Session 1	• Goal-setting and action-planning: Objective specification and commitment<br>• Self-monitoring: Alcohol-consumption logs<br>• Psychoeducation: Alcohol consumption and adverse consequences for obstructive sleep apnea<br>• Cognitive restructuring: Irrational, false or inaccurate beliefs about alcohol consumption<br>• Alcohol fading: Reduction of alcohol consumption/number of alcoholic drinks by 30% from baseline<br>• Breathing and relaxation techniques: Diaphragmatic breathing and progressive muscle relaxation
Session 2	• Review of behavioral goals, action plans, and compliance (participant's homework)<br>• Self-monitoring: Alcohol-consumption logs<br>• Cognitive restructuring: Irrational, false or inaccurate beliefs about smoking cessation<br>• Alcohol fading: Reduction of alcohol consumption/number of alcoholic drinks by 30% from week 1<br>• Stimulus control: Reduction/abstinence strategies<br>• Review of diaphragmatic breathing and progressive muscle relaxation
Session 3	• Review of behavioral goals, action plans, and compliance (participant's homework)<br>• Self-monitoring: Alcohol-consumption logs<br>• Vicarious and self-reinforcement: Changes in alcohol consumption achieved and benefits<br>• Abstinence planning: Setting the day when abstinence starts<br>• Stimulus control: Reduction/abstinence strategies<br>• Review of diaphragmatic breathing and progressive muscle relaxation
Session 4	• Review of behavioral goals, action plans, and compliance (participant's homework)<br>• Self-monitoring: Alcohol-consumption logs<br>• Vicarious and self-reinforcement: Changes in alcohol consumption achieved and benefits<br>• Problem solving and social skills: High risk situations for drinking alcohol and alternative behaviors<br>• Review of all intervention components: diaphragmatic breathing and progressive muscle relaxation, stimulus control<br>• Maintenance and relapse prevention: Difference between lapse and relapse

3.7. Assessment of Intervention Compliance and Integrity

Integrity of the intervention and treatment fidelity will be evaluated and ensured through the design and implementation of different strategies of process assessment, monitoring and enhancement in order to guarantee internal and external validity of the trial [86].

Firstly, regarding the study design and provider of intervention training, we developed a comprehensive hand-book for the qualified INTERAPNEA study intervention providers/professionals/training personnel of each module (nutrition, physical exercise, sleep hygiene, and tobacco and alcohol consumption). Each intervention manual identifies the theoretical model of the intervention and provides detailed descriptions of session objectives, treatment guidelines in accordance with each objective (i.e. contents, tasks and activities, recommendations, and timing), participant's homework, and material needed for each session. We will also provide each participant

with an adapted patient-handbook for each intervention component including descriptions of sessions, and work and logging sheets.

Secondly, we will ensure fidelity in the treatment delivery, receipt, and enactment through the use of these intervention protocols/manuals and monitoring of the implementation. Regarding the treatment delivery, the standardization of the intervention will support the protocol adherence of providers and the treatment differentiation (i.e. the delivery of the target treatment and no other). Furthermore, we will include a check-list for provider's self-report concerning the achievement of session objectives. With respect to the treatment receipt and enactment, fidelity will be assessed and confirmed through different strategies such as the structuring of the intervention around achievement-based objectives, collecting and reviewing of participants self-monitored data (daily steps log, sleep diaries, alcohol and tobacco consumption records), and information delivery in different formats (e.g. written in the handbooks, and verbal and visual in the sessions).

Finally, apart from the above mention strategies, we will consider complementary approaches in order to reduce participant drop-out rates and increase adherence such as prevention of commitments or vacation periods, use of well-equipped and conditioned facilities, and supervision by a qualified and certified pair of providers in each session motivating and supporting participants. Participants' attendance to each intervention session will be recorded by providers, and phone-calls will be made to assess causes of absence and determine further participation in the intervention.

3.7. Usual Care Arm

Participants with moderate-severe OSA randomly assigned to the usual care group (control) will receive, apart from CPAP treatment, a 30 min session of general advice on weight loss and lifestyle change from a sleep disordered-breathing specialist. Informative leaflets describing the positive effects of healthy nutrition, physical activity, sleep hygiene and tobacco and alcohol avoidance for OSA will also be provided to these participants. Additionally, the opportunity to receive the INTERAPNEA study intervention will be offered to this control group after the six month follow-up.

3.8. Serious Adverse Events

Serious adverse events occurring to participants from baseline to intervention endpoint and/or 6-month follow-up, related or unrelated to the study intervention or participation, will be collected and registered over the course of the trial by the study

personnel. These are defined as events that lead to death, life-threatening illness, permanent impairment, or hospitalization with a serious health condition.

3.9. Power and Sample Size

The sample size calculation and power of the study are based on the data of previously reported studies contrasted, combined, and synthesized in our recent systematic review and meta-analysis [1]. We considered following the formula $\boldsymbol{n} = \frac{2(Z_\alpha + Z_{1-\beta})^2\sigma^2}{\Delta^2}$, where n is the required sample size, $Z\alpha$ and $Z(1\text{-}\beta)$ are the constants set by convention according to the accepted α error and power of the study, respectively, σ is the estimated standard deviation, and Δ is the expected effect size. Therefore, we expect to detect an effect size of -8.36 on AHI (pooled raw mean difference of previous trials) [1], considering a type 1 error/α error of 5%, and a statistical power of 90%. Regarding the estimated AHI variability, we established an σ of 11.98, considering the AHI pooled standard deviation of all independent samples included in our previous research [1]. As a result, the expected sample size is of ≈ 35 participants per arm of our controlled clinical trial. However, assuming a maximum of a 17.25% drop-out rate (based on the average drop-out rate of previous studies [1]), we decided to recruit a total sample size of ≈ 42 participants for each study group. Thus, a total of ≈ 84 patients with moderate to severe OSA will be enrolled in the INTERAPNEA study. For practical and feasibility reasons, and based on our previous experience [87,88], the study will be conducted in sets of a maximum of 30 persons.

3.10. Statistical Analysis

We will perform descriptive and exploratory preliminary analyses of all the study variables to reveal violations of statistical assumptions, distributions, imbalances between the study groups, associations between study variables, covariates/confounders, amount of missing data, and drop-out patterns.

The intervention effects on primary and secondary study outcomes will be assessed through multi-level mixed analyses using the package *nlme* [89] from the R statistical program. This method will allow us to analyze differences including time and group allocation as within and between subject factors, respectively, adjusting for potential covariates or levels such as set of participants. Therefore, time and group/condition will be considered as fixed variables, and sets/waves of participants as a random factor. With these specifications, we will be able to control the intra-class correlation (per set of

participants nested within conditions) between scores at baseline and post-test or follow-up. Attrition propensity will be calculated using a model predicting the actual attrition with baseline values [90]. Baseline measurements included in these attrition propensity prediction models will include set of participants, participant's allocation, age, OSA severity, BMI, and motivation to change. All analyses will be performed with an intention-to-treat (ITT) approach, including all participants as originally allocated after randomization. Per-protocol comparison of groups including only those participants who fully completed the originally allocated treatment will also be computed and compared with ITT analysis results.

4. ORGANIZATION

The Sleep and Health Promotion Laboratory of the Mind, Brain and Behavior Research Center (CIMCYC) from the University of Granada (Granada, Spain) was responsible for the study design and organization, participant recruitment process, data collection and management, randomization and participant allocation, trial monitoring, and reporting of the study process and results. Participants previously diagnosed with moderate-to-severe OSA and potentially meeting the inclusion criteria were recruited from the collaborating sleep-disordered breathing unit of the Virgen de las Nieves University Hospital (Granada, Spain). Data collection at baseline and intervention endpoint, as well as implementation of the weight loss and lifestyle intervention, was performed in two different settings of the University of Granada (Granada, Spain): the Sleep and Health Promotion Laboratory (CIMCYC) and the Sport and Health University Research Institute (iMUDS). General and specific OSA standard care (i.e. continuous positive airway pressure [CPAP]) of all participants enrolled in the trial continued being provided by their primary care team from the Virgen de las Nieves University Hospital.

5. ETHICAL ISSUES

5.1. Institutional Ethics Committee Approval

This study conforms to the last revised Ethical Principles for Medical Research Involving Human Subjects comprised in the Declaration of Helsinki and written approvals will be sought from all relevant Research Ethics Committees (i.e. Institutional Review Board) before the recruitment of participants. Specifically, approval will be sought from the Research Ethics Committees of: (a) University of Granada (Granada,

Spain); (b) Virgen de las Nieves University Hospital (Granada, Spain); and (c) Junta de Andalucía (Spain).

5.2. Participant Consent

All participants will receive accurate information on the study assessments and intervention, and written informed consent from each participant will be obtained prior to any data collection. Signed copies of the Consent Form and a Patient Information Sheet will be given to participants. Those participants unable or not willing to give informed consent will not be eligible for enrollment in this study.

The Patient Information Sheet will also clearly state that participants have the right to withdraw from the study at any time without prejudice or explanation.

5.3. Confidentiality and Privacy

The confidentiality and privacy of all participants will be cautiously respected and ensured throughout the conduct of the study. All data will only be identified by a unique identification number/code given to each participant at the beginning of the trial; all data treatment and analyses being correspondingly blinded.

5.4. Provision of the Trial Intervention to All Participants

The interdisciplinary weight loss and lifestyle intervention will be offered to all participants randomly assigned to the usual-care/control group (i.e. CPAP alone) at the conclusion of the trial. This information is included in the patient information and consent forms.

6. REFERENCES

1. Carneiro-Barrera, A.; Díaz-Román, A.; Guillén-Riquelme, A.; Buela-Casal, G. Weight loss and lifestyle interventions for obstructive sleep apnea in adults: Systematic review and meta-analysis. (Unpublished)
2. Jordan, A.S.; McSharry, D.G.; Malhotra, A. Adult obstructive sleep apnea. Lancet 2014, 383, 736–747. doi:10.1016/S0140-6736(13)60734-5
3. Fu, Y.; Xia, Y.; Yi, H.; Xu, H.; Guan, J.; Yin, S. Meta-analysis of all-cause and cardiovascular mortality in obstructive sleep apnea with or without continuous positive airway pressure treatment. Sleep Breath. 2017, 21, 181–189. doi:10.1007/s11325-016-1393-1
4. Hoyos, C.M.; Drager, L.F.; Patel, S.R. OSA and cardiometabolic risk: What's the bottom line? Respirology 2017, 22, 420–429. doi:10.1111/resp.12984
5. Blackwell, T.; Yaffe, K.; Laffan, A.; Redline, S.; Ancoli-Israel, S.; Ensrud, K.E.; Song, Y.; Stone, K.L. Associations between sleep-disordered breathing, nocturnal hypoxemia, and subsequent cognitive decline in older community-dwelling men: The

Osteoporotic Fractures in Men Sleep Study. J. Am. Geriatr. Soc. 2015, 63, 453–461. doi:10.1111/jgs.13321
6. Guglielmi, O.; Gómez, A.I.S.; Jurado-Gámez, B.; Buela-Casal, G. Obstructive sleep apnea syndrome effects on quality of life and daytime sleepiness. Univ. Psychol. 2013, 12, 601–611. doi:10.11144/Javeriana.UPSY12-2.esah
7. Tregear, S.; Reston, J.; Schoelles, K.; Phillips, B. Obstructive sleep apnea and risk of motor vehicle crash: Systematic review and meta-analysis. J. Clin. Sleep Med. 2009, 5, 573–581.
8. Jurado-Gámez, B.; Guglielmi, O.; Gude, F.; Buela-Casal, G. Workplace accidents, absenteeism and productivity in patients with sleep apnea. Arch. Bronconeumol. 2015, 51, 213–218. doi:10.1016/j.arbr.2014.12.002
9. Senaratna, C.V.; Perret, J.L.; Lodge, C.J.; Lowe, A.J.; Campbell, B.E.; Matheson, M.C.; Hamilton, G.S.; Dharmage, S.C. Prevalence of obstructive sleep apnea in the general population: a systematic review. Sleep Med. Rev. 2017, 34, 70–81. doi:10.1016/j.smrv.2016.07.002
10. Senaratna, C.V.; English, D.R.; Currier, D.; Perret, J.L.; Lowe, A.; Lodge, C.; Russell, M.; Sahabandu, S.; Matheson, M.C.; Hamilton, G.S.; Dharmage, S.C. Sleep apnea in Australian men: Disease burden, co-morbidities, and correlates from the Australian longitudinal study on male health. BMC Public Health 2016, 16, 51–61. doi:10.1186/s12889-016-3703-8
11. Young, T.; Peppard, P.E.; Taheri, S. Excess weight and sleep-disordered breathing. J. Appl. Physiol. 2005, 99, 1592–1599. doi:10.1152/japplphysiol.00587.2005
12. Ong, C.W.; O'Driscoll, D.M.; Truby, H.; Naughton, M.T.; Hamilton, G.S. The reciprocal interaction between obesity and obstructive sleep apnea. Sleep Med. Rev. 2013, 17, 123–131. doi:10.1016/j.smrv.2012.05.002
13. World Health Organization. Obesity and overweight. Available on: https://www.who.int/news-room/fact-sheets/detail/obesity-and-overweight
14. Epstein, L.J.; Kristo, D.; Strollo, P.J.; Friedman, N.; Malhotra, A.; Patil, S.P.; Ramar, K.; Rogers, R.; Schwab, R.J.; Weaver, E.M.; Weinstein, M.D. Clinical guideline for the evaluation, management and long-term care of obstructive sleep apnea in adults. J. Clin. Sleep Med. 2009, 5, 263–276.
15. Zhao, Y.Y.; Wang, R.; Gleason, K.J.; Lewis, E.F.; Quan, S.F.; Toth, C.M.; Morrical, M.; Rueschman, M.; Weng, J.; Ware, J.H.; Mittleman, M.A.; Redline, S. Effect of continuous positive airway pressure treatment on health-related quality of life and sleepiness in high cardiovascular risk individuals with sleep apnea: Best apnea interventions for research (BestAIR) trial. Sleep 2017, 40, zsx040. doi:10.1093/sleep/zsx040
16. Sánchez, A.I.; Martínez, P.; Miró, E.; Bardwell, W.A.; Buela-Casal, G. CPAP and behavioral therapies in patients with obstructive sleep apnea: effects on daytime sleepiness, mood, and cognitive function. Sleep Med. Rev. 2009, 13, 223–233. doi:10.1016/j.smrv.2008.07.002
17. Jurado-Gámez, B.; Guglielmi, O.; Gude-Sampedro, F.; Buela-Casal, G. Effect of CPAP therapy on job productivity and psychosocial occupational health in patients

with moderate to severe sleep apnea. Sleep Breath. 2015, 19, 1293–1299. doi:10.1007/s11325-015-1162-6
18. Wolkove, N.; Baltzan, M.; Kamel, H.; Dabrusin, R.; Palayew, M. Long-term compliance with continuous positive airway pressure in patients with obstructive sleep apnea. Can. Respir. J. 2008, 15, 365–369. doi:10.1155/2008/534372
19. Morgenthaler, T.I.; Kapen, S.; Lee-Chiong, T.; Alessi, C.; Boehlecke, B.; Brown, T.; Coleman, J.; Friedman, L.; Kapur, V.; Owens, J.; Pancer, J.; Swick, T. Practice parameters for the medical therapy of obstructive sleep apnea. Sleep 2006, 29, 1031–1035.
20. Kolla, B.P.; Foroughi, M.; Saeidifard, F.; Chakravorty, S.; Wang, Z.; Mansukhani, M.P. The impact of alcohol on breathing parameters during sleep: a systematic review and meta-analysis. Sleep Med. Rev. 2018, 42, 59–67. doi:10.1016/j.smrv.2018.05.007
21. Krishnan, V.; Dixon-Williams, S.; Thornton, J.D. Where there is smoke… there is sleep apnea: Exploring the relationship between smoking and sleep apnea. Chest 2014, 146, 1673–1680. doi:10.1378/chest.14-0772.
22. American Academy of Sleep Medicine. International Classifcation of Sleep Disorders, 3rd ed; American Academy of Sleep Medicine: Darien, IL, USA, 2014
23. Basoglu, O.K.; Tasbakan, M.S. Gender differences in clinical and polysomnographic features of obstructive sleep apnea: A clinical study of 2827 patients. Sleep Breath. 2018, 22, 241–249. doi:10.1007/s11325-017-1482-9
24. Harreiter, J.; Kautzky-Willer, A. Sex and gender differences in prevention of type 2 diabetes. Front. Endocrinol. 2018, 9, 220. doi:10.3389/fendo.2018.00220
25. Schulz, K.F.; Grimes, D.A. Generation of allocation sequences in randomised trials: chance, not choice. Lancet 2002, 359, 515–519. doi:10.1016/S0140-6736(02)07683-3
26. Friedberg, J.P.; Lipsitz, S.R.; Natarajan, S. Challenges and recommendations for blinding in behavioral interventions illustrated using a case study of a behavioral intervention to lower blood pressure. Patient Educ. Couns. 2010, 78, 5–11. doi:10.1016/j.pec.2009.04.009
27. Kapur, V.K.; Auckley, D.H.; Chowdhuri, S.; Kuhlmann, D.C.; Mehra, R.; Ramar, K.; Harrod, C.G. Clinical Practice Guideline for Diagnostic Testing for Adult Obstructive Sleep Apnea: An American Academy of Sleep Medicine Clinical Practice Guideline. J. Clin. Sleep Med. 2017, 13, 479–504. doi:10.5664/jcsm.6506.
28. Boudewyns, A.; Sforza, E.; Zamagni, M.; Krieger, J. Respiratory effort during sleep apneas after interruption of long-term CPAP treatment in patients with obstructive sleep apnea. Chest 1996, 110, 120–127. doi:10.1378/chest.110.1.120
29. Berry, R.B.; Budhiraja, R.; Gottlieb, D.J.; Gozal, D.; Iber, C.; Kapur, V.K.; Marcus, C.L.; Mehra, R.; Parthasarathy, S.; Quan, S.F.; Redline, S.; Strohl, K.P.; Ward, S.L.D.; Tangredi, M.M. Rules for scoring respiratory events in sleep: update of the 2007 AASM manual for the scoring of sleep and associated events. Deliberations of the sleep apnea definitions task force of the American Academy of Sleep Medicine. J. Clin. Sleep Med. 2012, 8, 597–619. doi:10.5664/jcsm.217

30. Jasper, H.H. Report of the committee on methods of clinical examination in electroencephalography: 1957. Electroencephalogr. Clin. Neurophysiol. 1958, 10, 370–375. doi:10.1016/0013-4694(58)90053-1
31. Rechtschaffen, A.; Kales, A. A manual of standardized terminology, techniques and scoring system of sleep stages in human subjects; Brain Information Service, Brain Research Institute, University of California: Los Angeles, CA, USA, 1968.
32. Mokhlesi, B.; Varga, A.W. Obstructive sleep apnea and cardiovascular disease: REM sleep matters! Am. J. Respir. Crit. Care Med. 2018, 197, 554–556. doi:10.1164/rccm.201710-2147ED.
33. Oja, P.; Laukkanen, R.; Pasanen, M.; Tyry, T.; Vuori, I. A 2-km walking test for assessing the cardiorespiratory fitness of healthy adults. Int. J. Sports Med. 1991, 12, 356–362. doi:10.1055/s-2007-1024694
34. Oja, P.; Tuxworth, B. Eurofit for adults: Assessment of health-related fitness. Council of Europe Publishing: Finland, 1995, pp. 1–13.
35. Ortega, F.B.; Sánchez-López, M.; Solera-Martínez, M.; Fernández-Sánchez, A.; Sjöström, M.; Martínez-Vizcaino, V. Self-reported and measured cardiorespiratory fitness similarly predict cardiovascular disease risk in young adults. Scand. J. Med. Sci. Sports 2013, 23, 749–757. doi:10.1111/j.1600-0838.2012.01454.x
36. Marfell-Jones, M.J.; Stewart, A.D.; de Ridder, J.H. International standards for anthropometric assessment; International Society for the Advancement of Kinanthropometry: Wellington, New Zealand, 2012.
37. Schröder, H.; Fitó, M.; Estruch, R.; Martínez-González, M.A.; Corella, D.; Salas-Salvadó, J.; Lamuela-Raventós, R.; Ros E.; Salaverría, I.; Fiol, M.; Lapetra, J.; Vinyoles, E.; Gómez-Gracia, E.; Lahoz, C.; Serra-Majem, L.; Pintó, X.; Ruiz-Gutierrez, V.; Covas, M.I. A short screener is valid for assessing Mediterranean diet adherence among older Spanish men and women. J. Nutr. 2011, 141, 1140–1145. doi:10.3945/jn.110.135566
38. Banna, J.C.; Townsend, M.S. Assessing factorial and convergent validity and reliability of a food behavior checklist for Spanish-speaking participants in US Department of Agriculture nutrition education programmes. Public Health Nutr. 2011, 14, 1165–1176. doi:10.1017/S1368980010003058
39. Becoña, E.; Gómez-Durán, B.; Alvarez-Soto, E.; García, M.P. [Scores of Spanish smokers on Fagerström's Tolerance Questionnaire]. Psychol. Rep. 1992, 71, 1227–1233.
40. Ferrer, M.; Vilagut, G.; Monasterio, C.; Montserrat, J.M.; Mayos, M.; Alonso, J. [Measurement of the perceived impact of sleep problems: the Spanish version of the functional outcomes sleep questionnaire and the Epworth sleepiness scale]. Med. Clin. (Barc). 1999, 113, 250–255.
41. Sanz, J.; Izquierdo, A.; García-Vera, M.P.; Dpto. I+D Pearson Clinical & Talent Assessment. BDI-FS, Inventario de Depresión de Beck para pacientes médicos; Pearson: Madrid, España, 2011.
42. Buela-Casal, G.; Guillén-Riquelme, A.; Seisdedos-Cubero, N. Cuestionario de Ansiedad Estado-Rasgo: Adaptación española, 9th ed.; TEA Ediciones: Madrid, España, 2016.

43. Spielberger, C.D.; Agudelo, D.; Buela-Casal, G. Inventario de Depresión Estado/Rasgo (IDER); TEA Ediciones: Madrid, España, 2008.
44. Rezaeitalab, F.; Moharrari, F.; Saberi, S.; Asadpour, H.; Rezaeetalab, F. The correlation of anxiety and depression with obstructive sleep apnea syndrome. J. Res. Med. Sci. 2014, 19, 205–210.
45. Chiner, E.; Arriero, J.M.; Signes-Costa, J.; Marco, J.; Fuentes, I. [Validation of the Spanish version of the Epworth Sleepiness Scale in patients with a sleep apnea syndrome]. Arch. Bronconeumol. 1999, 35, 422–427.
46. Dinges, D.F.; Powell, J.W. Microcomputer analyses of performance on a portable, simple visual RT task during sustained operations. Behav. Res. Meth. Instrum. Comp. 1985, 17, 652–655. doi:10.3758/BF03200977
47. Khitrov, M.Y.; Laxminarayan, S.; Thorsley, D.; Ramakrishnan, S.; Rajaraman, S.; Wesensten, N.J.; Reifman, J. PC-PVT: A platform for psychomotor vigilance task testing, analysis, and prediction. Behav. Res. Methods. 2014, 46, 140–147. doi:10.3758/s13428-013-0339-9
48. Reifman, J.; Kumar, K.; Khitrov, M.Y.; Liu, J.; Ramakrishnan, S. PC-PVT 2.0: An updated platform for psychomotor vigilance task testing, analysis, prediction, and visualization. J. Neurosci. Methods. 2018, 304, 39–45. doi:10.1016/j.jneumeth.2018.04.007
49. Becker, N.B.; de Jesus, S.N.; Viseu, J.N.; Stobäus, C.D.; Guerreiro, M.; Domingues, R.B. Depression and quality of life in older adults: Mediation effect of sleep quality. Int. J. Clin. Health Psychol. 2018, 18, 8–17. doi:10.1016/j.ijchp.2017.10.002
50. Wersebe, H.; Lieb, R.; Meyer, A.H.; Miche, M.; Mikoteit, T.; Imboden, C.; Hoyer, J.; Bader, K.; Hatzinger, M.; Gloster, A.T. Well-being in major depression and social phobia with and without comorbidity. Int. J. Clin. Health Psychol. 2018, 18, 201–208. doi:10.1016/j.ijchp.2018.06.004
51. Royuela, A.; Macías, J.A. Propiedades clinimétricas de la versión castellana del Cuestionario de Pittsburgh. Vigilia-Sueño 1997, 9, 81–94.
52. Catalán, P.; Martínez, A.; Herrejón, A.; Martínez-García, M.Á.; Soler-Cataluña, J.J.; Román-Sánchez, P.; Pinel, J.; Blanquer, R. [Internal consistency and validity of the Spanish version of the quality of life questionnaire specific for obstructive sleep apnea: Sleep apnea quality of life index]. Arch. Bronconeumol. 2012, 48, 431–442. doi:10.1016/j.arbres.2012.05.004
53. Alonso, J.; Prieto, L.; Antó, J.M. [The Spanish version of the SF-36 Health Survey (the SF-36 health questionnaire): an instrument for measuring clinical results]. Med. Clin. (Barc). 1995, 104, 771–776.
54. Lobo, A.; Pérez-Echeverría, M.J.; Artal, J. Validity of the scaled version of the General Health Questionnaire (GHQ-28) in a Spanish population. Psychol. Med. 1986, 16, 135–140.
55. Adan, A.; Almirall, H. Horne and Östberg morningness–eveningness questionnaire: A reduced scale. Pers. Indiv. Differ. 1991, 12, 241–253. doi:10.1016/0191-8869(91)90110-W

56. Kim, L.J.; Coelho, F.M.; Hirotsu, C.; Bittencourt, L.; Tufik, S.; Andersen, M.L. Is the chronotype associated with obstructive sleep apnea? Sleep Breath. 2015, 19, 645–651. doi:10.1007/s11325-014-1070-1
57. Lucassen, E.A.; Zhao, X.; Rother, K.I.; Mattingly, M.S.; Courville, A.B.; de Jonge, L.; Csako, G.; Cizza, G. Evening chronotype is associated with changes in eating behavior, more sleep apnea, and increased stress hormones in short sleeping obese individuals. PLoS One 2013, 8, e56519. doi:10.1371/journal.pone.0056519
58. Prochaska, J.O.; Velicer, W.F. The transtheoretical model of health behavior change. Am. J. Health Promot. 1997, 12, 38–48. doi:10.4278/0890-1171-12.1.38
59. Burgess, E.; Hassmén, P.; Welvaert, M.; Pumpa, K.L. Behavioral treatment strategies to improve adherence to lifestyle intervention programmes in adults with obesity: A systematic review and meta-analysis. Clin. Obes. 2017, 7, 105–114. doi:10.1111/cob.12180
60. St-Onge, M.P.; Mikic, A.; Pietrolungo, C.E. Effects of diet on sleep quality. Adv. Nutr. 2016, 7, 938–949. doi:10.3945/an.116.012336
61. Bozkurt, N.C.; Cakal, E.; Sahin, M.; Ozkaya, E.C.; Firat, H.; Delibasi, T. The relation of serum 25-hydroxyvitamin-D levels with severity of obstructive sleep apnea and glucose metabolism abnormalities. Endocrine 2012, 41, 518–525. doi:10.1007/s12020-012-9595-1
62. Gao, Q.; Kou, T.; Zhuang, B.; Ren, Y.; Dong, X.; Wang, Q. The Association between vitamin D deficiency and sleep disorders: A systematic review and meta-analysis. Nutrients 2018, 10, 1395. doi:10.3390/nu10101395
63. Cao, Y.; Zhen, S.; Taylor, A.W.; Appleton, S.; Atlantis, E.; Shi, Z. Magnesium intake and sleep disorder symptoms: Findings from the Jiangsu Nutrition Study of Chinese adults at five-year follow-up. Nutrients 2018, 10, 1354. doi:10.3390/nu10101354
64. McHill, A.W.; Wright, K.P.Jr. Role of sleep and circadian disruption on energy expenditure and in metabolic predisposition to human obesity and metabolic disease. Obes. Rev. 2017, 18, 15–24. doi:10.1111/obr.12503
65. St-Onge, M.P.; Wolfe, S.; Sy, M.; Shechter, A.; Hirsch, J. Sleep restriction increases the neuronal response to unhealthy food in normal-weight individuals. Int. J. Obes. 2014, 38, 411–416. doi:10.1038/ijo.2013.114
66. St-Onge, M.P.; McReynolds, A.; Trivedi, Z.B.; Roberts, A.L.; Sy, M.; Hirsch, J. Sleep restriction leads to increased activation of brain regions sensitive to food stimuli. Am. J. Clin. Nutr. 2012, 95, 818–824. doi:10.3945/ajcn.111.027383
67. Wallace, A.; Bucks, R.S. Memory and obstructive sleep apnea: A meta-analysis. Sleep 2013, 36, 203–220. doi:10.5665/sleep.2374
68. Martin, A.A.; Davidson, T.L.; McCrory, M.A. Deficits in episodic memory are related to uncontrolled eating in a sample of healthy adults. Appetite 2018, 124, 33–42. doi:10.1016/j.appet.2017.05.011
69. Aiello, K.D.; Caughey, W.G.; Nelluri, B2.; Sharma, A.; Mookadam, F.; Mookadam, M. Effect of exercise training on sleep apnea: A systematic review and meta-analysis. Respir. Med. 2016, 116, 85–92. doi:10.1016/j.rmed.2016.05.015
70. Iftikhar, I.H.; Kline, C.E.; Youngstedt, S.D. Effects of exercise training on sleep apnea: a meta-analysis. Lung 2014, 192, 175e84. doi:10.1007/s00408-013-9511-3

71. Shook, R.P.; Hand, G.A.; Drenowatz, C.; Hebert, J.R.; Paluch, A.E.; Blundell, J.E.; Hill, J.O.; Katzmarzyk, P.T.; Church, T.S.; Blair, S.N. Low levels of physical activity are associated with dysregulation of energy intake and fat mass gain over 1 year. Am. J. Clin. Nutr. 2015, 102, 1332–1338. doi:10.3945/ajcn.115.115360
72. Shechter, A. Obstructive sleep apnea and energy balance regulation: A systematic review. Sleep Med. Rev. 2017, 34, 59–69. doi:10.1016/j.smrv.2016.07.001
73. Ueno, L.M.; Drager, L.F.; Rodrigues, A.C.; Rondon, M.U.; Braga, A.M.; Mathias, W.Jr.; Krieger, E.M.; Barretto, A.C.; Middlekauff, H.R.; Lorenzi-Filho, G.; Negrão C.E.. Effects of exercise training in patients with chronic heart failure and sleep apnea. Sleep 2009, 32, 637–647. doi:10.1093/sleep/32.5.637
74. Redolfi, S.; Arnulf, I.; Pottier, M.; Bradley, T.D.; Similowski, T. Effects of venous compression of the legs on overnight rostral fluid shift and obstructive sleep apnea. Respir. Physiol. Neurobiol. 2011, 175, 390–393. doi:10.1016/j.resp.2011.01.001
75. Evans, R.A.; Dolmage, T.E.; Robles, P.G.; Brooks, D.; Goldstein, R.S. The effects of exercise modality and intensity on energy expenditure and cardiorespiratory response in adults with obesity and treated obstructive sleep apnea. Chron. Respir. Dis. 2017, 14, 342–351. doi:10.1177/1479972316643699
76. Buela-Casal, G.; Sierra, J. C. Evaluación y tratamiento de los trastornos del sueño [Evaluation and treatment of sleep disorders]. In Manual de Evaluación y Tratamientos Psicológicos, 2nd ed.; Buela-Casal, G., Sierra, J.C., Eds.; Biblioteca Nueva: Madrid, España, 2004; pp. 393–438.
77. Kitamura, T.; Miyazaki, S.; Koizumi, H.; Takeuchi, S.; Tabata, T.; Suzuki, H. Sleep hygiene education for patients with obstructive sleep apnea. Sleep Biol. Rhythms 2016, 14, 101–106. doi:10.1007/s41105-015-0015-9
78. Chakravorty, I.; Cayton, R.M.; Szczepura, A. Health utilities in evaluating intervention in the sleep apnea/hypopnea syndrome. Eur. Respir. J. 2002, 20, 1233–1238.
79. Ackel-D'Elia, C.; da Silva, A.C.; Silva, R.S.; Truksinas, E.; Sousa, B.S.; Tufik, S.; de Mello, M.T.; Bittencourt, L.R. Effects of exercise training associated with continuous positive airway pressure treatment in patients with obstructive sleep apnea syndrome. Sleep Breath. 2012, 16, 723–735. doi:10.1007/s11325-011-0567-0
80. Schrand, J.R. Is sleep apnea a predisposing factor for tobacco use? Med. Hypotheses 1996, 47, 443 - 448.
81. Becoña, E. Programa para dejar de fumar [Program for smoking cessation]; Nova Galicia Edicións: Vigo, España, 2007.
82. Foxx, R. M., & Brown, R. A. (1979). Nicotine fading, self-monitoring for cigarette abstinence or controlled smoking. J. Applied Behav. Anal. 1979, 12, 115–125.
83. Becoña, E.; García, M.P. Nicotine fading and smokeholding methods to smoking cessation. Psychol. Rep. 1993, 73, 779–786.
84. Simou, E.; Britton, J.; Leonardi-Bee, J. Alcohol and the risk of sleep apnea: a systematic review and meta-analysis. Sleep Med. 2018, 42, 38–46. doi:10.1016/j.sleep.2017.12.005
85. Issa, F.G.; Sullivan, C.E. Alcohol, snoring and sleep apnea. J. Neurol. Neurosurg. Psychiatry 1982, 45, 353–359.

86. Borrelli, B. The assessment, monitoring, and enhancement of treatment fidelity in public health clinical trials. J. Public Health Dent. 2011, 71, 52–63. doi:10.1111/j.1752-7325.2011.00233.x
87. Amaro-Gahete, F.J.; De-la-O, A.; Jurado-Fasoli, L.; Espuch-Oliver, A.; Robles-González, L.; Navarro-Lomas, G.; de Haro, T.; Femia, P.; Castillo, M.J.; Gutierrez, A. Exercise training as S-Klotho protein stimulator in sedentary healthy adults: Rationale, design, and methodology. Contemp. Clin. Trials Commun. 2018, 11, 10–19. doi:10.1016/j.conctc.2018.05.013
88. Sánchez-Delgado, G.; Martinez-Tellez, B.; Olza, J.; Aguilera, C.M.; Labayen, I.; Ortega, F.B.; Chillon, P.; Fernandez-Reguera, C.; Alcantara, J.M.A.; Martinez-Avila, W.D.; Muñoz-Hernandez, V.; Acosta, F.M.; Prados-Ruiz, J.; Amaro-Gahete, F.J.; Hidalgo-Garcia, L.; Rodriguez, L..; Ruiz, Y.A..; Ramirez-Navarro, A.; Muros-de Fuentes, M.A.; García-Rivero, Y.; Sanchez-Sanchez, R.; de Dios Beas Jimenez, J.; de Teresa, C.; Navarrete, S.; Lozano, R.; Brea-Gomez, E.; Rubio-Lopez, J.; Ruiz, M.R.; Cano-Nieto, A.; Llamas-Elvira, J.M.; Jimenez Rios, J.A.; Gil, A.; Ruiz, J.R. Activating brown adipose tissue through exercise (ACTIBATE) in young adults: Rationale, design and methodology. Contemp. Clin. Trials 2015, 45, 416–425. doi:10.1016/j.cct.2015.11.004
89. Pinheiro, J.; Bates, D.; DebRoy, S.; Sarkar, D.; R Core Team. nlme: Linear and nonlinear mixed effects models. R package version 3.1-137. Available on: https://CRAN.R-project.org/package=nlme
90. Berger, V.W. The reverse propensity score to detect selection bias and correct for baseline imbalances. Stat Med. 2005, 24, 2777–2787. doi:10.1002/sim.2141

An investigator-initiated and conducted, randomized, controlled trial of INTERdisciplinary weight loss and lifestyle intervention for the treatment of moderate-to-severe obstructive sleep APNEA

PROTOCOL

CONTACT DETAILS

INTERAPNEA Research Team
Sleep and Health Promotion Laboratory
Mind, Brain, and Behavior Research Center (CIMCYC)
University of Granada
Campus Universitario de Cartuja, C/ del Profesor Clavera S/N,
18011 Granada, Spain
Email: acarneiro@ugr.es

CONTENTS

1. BACKGROUND AND RATIONALE 3

1.1. OSA — A MAJOR HEALTH PROBLEM 3

1.2. CPAP — FIRST-LINE TREATMENT FOR OSA 3

1.3. WEIGHT LOSS AND LIFESTYLE INTERVENTION FOR OSA 4

2. AIMS AND OBJECTIVES 4

2.1. PRIMARY AIM 4

2.2. SECONDARY AIMS 5

3. METHODS 6

3.1. DESIGN 6

3.2. ELIGIBILITY CRITERIA 6

3.3. RECRUITMENT, ENROLLMENT AND RANDOMIZATION 7

3.4. ASSESSMENTS 9

3.5. ENDPOINTS 11

3.6. WEIGHT LOSS AND LIFESTYLE INTERVENTION ARM 16

3.7. ASSESSMENT OF INTERVENTION COMPLIANCE AND INTEGRITY 24

3.8. USUAL CARE ARM 25

3.9. SERIOUS ADVERSE EVENTS 25

3.10. POWER AND SAMPLE SIZE 25

3.11. STATISTICAL ANALYSIS 26

4. ORGANIZATION 27

5. ETHICAL ISSUES 27

5.1. INSTITUTIONAL ETHICS COMMITTEE APPROVAL 27

5.2. PARTICIPANT CONSENT 27

5.3. CONFIDENCIALITY AND PRIVACY 28

5.4. PROVISION OF THE TRIAL INTERVENTION TO ALL PARTICIPANTS 28

6. REFERENCES 28

1. BACKGROUND AND RATIONALE

1.1. OSA — A Major Public Health Problem

Obstructive sleep apnea (OSA), produced by repeated upper airway collapse during sleep, has increasingly become the focus of numerous current interdisciplinary research attributed not only to its high prevalence but also to the wide range of adverse health consequences of this condition [1]. The repeated events of complete (apnea) or partial (hypopnea) pharyngeal obstruction occurred while sleeping lead to intermittent hypoxic episodes, hypercapnia, sleep fragmentation, and upsurges of sympathetic activity [2]. Driven by these short-term consequences, OSA is closely related to increased morbidity and mortality [3], including cardio-metabolic disorders [4], neurocognitive abnormalities [5], impaired daily functioning and mood [6], and greater risk of vehicle and occupational accidents [7,8].

It has recently been estimated that up to 38% of adults suffer from OSA, being more prevalent in the male sex, the elderly, and in those who are obese [9]. OSA risk factors, therefore, include obesity, sex, age, and adverse lifestyle habits such as sedentariness, poor nutrition, smoking, and alcohol intake [10]. According to epidemiological studies, nearly 60% of moderate to severe OSA is attributable to obesity [11], which contributes to alterations of the airway anatomy and collapsibility, respiratory modulation, resting lung volume, and neurohormonal mediators on ventilation [12]. Given the exponential increase of obesity prevalence in the overall population, which has nearly tripled since 1975 — 39% of adults aged 18 years and over in 2016 —, OSA prevalence is not only worryingly high but also likely to rise in upcoming years [13].

1.2. CPAP — First-Line Treatment for OSA

The current treatment of choice is continuous positive airway pressure (CPAP) [14], a mechanical device used to maintain upper airway patency, thereby improving OSA main symptoms and consequences through the reduction of the number of apnea-hypopnea episodes per hour of sleep (i.e. apnea-hypopnea index, AHI) [15-17]. However, CPAP is a chronic day-to-day treatment — it does not cure OSA in the long-term —, and its use may be rejected or abandoned due to discomfort and/or other inconveniences [18]. Most importantly, CPAP does not address the major high-risk factors of OSA, i.e. obesity and adverse lifestyles.

1.3. Weight Loss and Lifestyle Intervention for OSA

Alternative or combined behavioral interventions including weight loss through dietary approaches and exercise, sleep hygiene, and avoidance of alcohol and tobacco consumption are required and strongly recommended in the most recent practical guidelines from the American Academy of Sleep Medicine (AASM) [14,19]. According to our recently published systematic review and meta-analysis on the effectiveness of these interventions [1], the combination of diet and exercise may be an effective treatment in improving OSA outcomes in middle-aged males with moderate to severe OSA.

Yet, the number of reported randomized controlled trials addressing both weight loss components as a combination was significantly low and only included effects on specific OSA outcomes such as AHI, oxygen desaturation index, and excessive daytime sleepiness [1]. Furthermore, no original studies actively focusing on the cessation of tobacco and alcohol consumption were found [1], factors which have been shown to be common in patients with OSA and associated with the worsening of this condition [20-21]. Thus, the actual effectiveness of potential interdisciplinary interventions for the improvement of OSA main symptoms and consequences still remains unclear. Considering the vast and severe OSA consequences and comorbidities, with obesity being a major risk factor for this condition, there is a need for well-designed studies comprising all these aspects and evaluating the potential clinical and economic relevance of these interventions for OSA and related diseases.

2. AIMS AND OBJECTIVES

The interdisciplinary weight loss and lifestyle intervention for OSA (INTERAPNEA) trial aims to determine the efficacy of a novel eight-week interdisciplinary weight loss and lifestyle intervention for the improvement of OSA and comorbidities in overweight/obese adults with CPAP-treated moderate-to-severe OSA.

2.1. Primary Aim

The primary aim of the INTERAPNEA trial is to design, implement and test the efficacy of an eight-week interdisciplinary weight loss and lifestyle intervention combined with usual-care (i.e. CPAP) to improve OSA severity (i.e. reduction of AHI) in overweight/obese adults with moderate-to-severe OSA. We hypothesize that the eight-week interdisciplinary weight loss and lifestyle intervention will lead to a greater and

significant reduction of AHI and/or even remission of OSA as compared with usual-care alone (i.e. CPAP).

2.2. Secondary Aims

The secondary aims of this trial include the determination of the efficacy of the eight-week interdisciplinary weight loss and lifestyle intervention combined with usual-care (i.e. CPAP) to improve other sleep-related outcomes, body weight and composition, OSA-related coexisting conditions/comorbidities (cardiometabolic risk), health-related quality of life, and daily functioning and mood, in overweight/obese adults with moderate-to-severe OSA. Accordingly, we hypothesize that:

- Relative to usual-care alone (i.e. CPAP), an eight-week interdisciplinary weight loss and lifestyle intervention combined with usual-care will lead to a significant improvement of oxyhemoglobin saturation outcomes, sleep efficiency and maintenance, sleep architecture, and subjective sleep quality and sleepiness.
- Relative to usual-care alone (i.e. CPAP), an eight-week interdisciplinary weight loss and lifestyle intervention combined with usual-care will lead to a significant reduction of body weight and improvement of other anthropometric and body composition outcomes (neck, chest and waist circumferences, fat mass, and visceral adipose tissue).
- Relative to usual-care alone (i.e. CPAP), an eight-week interdisciplinary weight loss and lifestyle intervention combined with usual-care will lead to a significant improvement of OSA-related cardiovascular risk outcomes (hypertension, dyslipidemia, insulin resistance, and liver diseases).
- Relative to usual-care alone (i.e. CPAP), an eight-week interdisciplinary weight loss and lifestyle intervention combined with usual-care will lead to a significant improvement of health-related quality of life and overall psychological health including daily functioning and mood.

3. METHODS

3.1. Design

The INTERAPNEA study (ClinicalTrials.gov ID: NCT03851653) is an investigator-initiated, randomized, parallel-group, open-label trial designed to evaluate the effects of an eight-week interdisciplinary weight loss and lifestyle intervention combined with usual-care (i.e. CPAP), as compared with usual-care alone, on OSA severity (i.e. apnea-hypopnea index [AHI]; number of apneas and hypopneas per hour of sleep), and OSA-related comorbidities among adults with moderate-to-severe OSA.

3.2. Eligibility Criteria

Eligible participants will be adults previously diagnosed with moderate-to-severe OSA (AHI equal or greater than 15 [22]) from the province of Granada (Spain). They must be between 18 and 65 years old, and have a body mass index (BMI) equal to or greater than 25 kg/m^2. A full list of the study's inclusion and exclusion criteria are exposed in Table 1. Due to the well-evidenced higher incidence and prevalence of OSA in the male sex [9] and the differences in OSA phenotypes between men and women [23], we will only include male participants in the study. Furthermore, non-pharmacological and non-surgical weight loss interventions have been shown to be less effective in women [1,24], such that different approaches are needed in this population with OSA.

Table 1. Eligibility criteria.

Inclusion criteria	Exclusion criteria
• Men aged 18–65 years<br>• CPAP-treated moderate to severe OSA (AHI equal to or greater than 15 events/hour)<br>• BMI equal to or greater than 25 kg/m^2<br>• Not participating in a weight loss program<br>• Willing to provide informed consent and acceptance of random group assignment	• Presence of any other primary sleep disorder<br>• Presence of any mental disorder (including depression, anxiety, and addiction to alcohol or other substances)<br>• Presence of any other severe organic disease, except for those comorbid to OSA<br>• Regular use of neuroleptic, sedative or hypnotic drugs, or any other medication that may cause sleep disturbances or increased daytime sleepiness

AHI = apnea-hypopnea index; BMI = body mass index; CPAP = continuous positive airway pressure; OSA = obstructive sleep apnea.

Potential participants will be medically examined and must complete a health history revision prior to their inclusion in the study in order to ensure no hindrance/harm related to the assessment and intervention protocols. Should any incident or medical problem arise during the intervention, participants will be physically and psychologically examined and, if necessary, excluded from the study. Clinical trial liability insurance will be contracted for the INTERAPNEA study, providing legal and financial protection to the sponsor-investigators, and compensation to participants in the case of an injury or any damage incurred in and as a result of the study.

3.3. Recruitment, Enrollment and Randomization

Recruitment

The recruitment of participants will be performed using different strategies including enrollment from the collaborating hospital sleep unit, and use of mass media (e.g. press, magazines, radio and television news, and websites). A brief in-person or phone screening will be conducted on potentially interested participants to provide general information about the study and determine suitability of inclusion. Patients willing to participate and appearing to meet the inclusion criteria will be required to attend an in-person briefing on the rationale and study aims, inclusion and exclusion criteria, assessments to be performed, and components and characteristics of the intervention. After clarification by the research staff of any participant's doubts or questions, signatures of informed consent will be obtained from participants that meet the eligibility criteria, and appointments for the baseline assessment will be given. Participant flow from recruitment to randomization stages are shown in Figure 1.

Enrollment

Upon obtaining signed informed consents, participant demographics and medical history will be collected, and a medical/physical examination will be performed to ensure feasibility of participant inclusion in the study. Subsequently, a sleep study through a complete full-night polysomnography and other sleep measurements (daytime sleepiness, sleep quality, circadian preference, and functional outcomes of OSA) will be conducted on and taken from each participant. Furthermore, lifestyle habits such as diet, exercise and tobacco and alcohol consumption will also be measured, as well as subjective health-related quality of life, and depressive and anxiety symptoms related to OSA. After completion of participant's medical and sleep studies, objective measurements of

cardiorespiratory fitness and body composition will be taken from each participant. All test trials will therefore be performed over three different days during a one to two-week period.

Randomization

After completing baseline measurements, eligible participants will be randomly assigned to either a control group or an interdisciplinary intervention group using a computer generated simple (unrestricted) randomization [25]. Each participant will be specifically informed of which arm they have been assigned to and requested not to reveal their allocation to the research staff involved in further assessments. Bias related to unblinded participants, treatment counsellors and/or outcome assessors affecting data validity will be addressed by achieving different levels of blinding across the study personnel and participants where feasible. Therefore, study personnel responsible for data collection and analysis will be blinded to allocation assignments at the follow-ups, and blinding of participants to details of study manuals and hypothesis will be attained. When blinding is not possible, rigorous procedures of standardization of data collection and intervention, through study manuals and continuous assessment of fidelity, will be followed to avoid potential bias and ensure internal and external validity of the study [26].

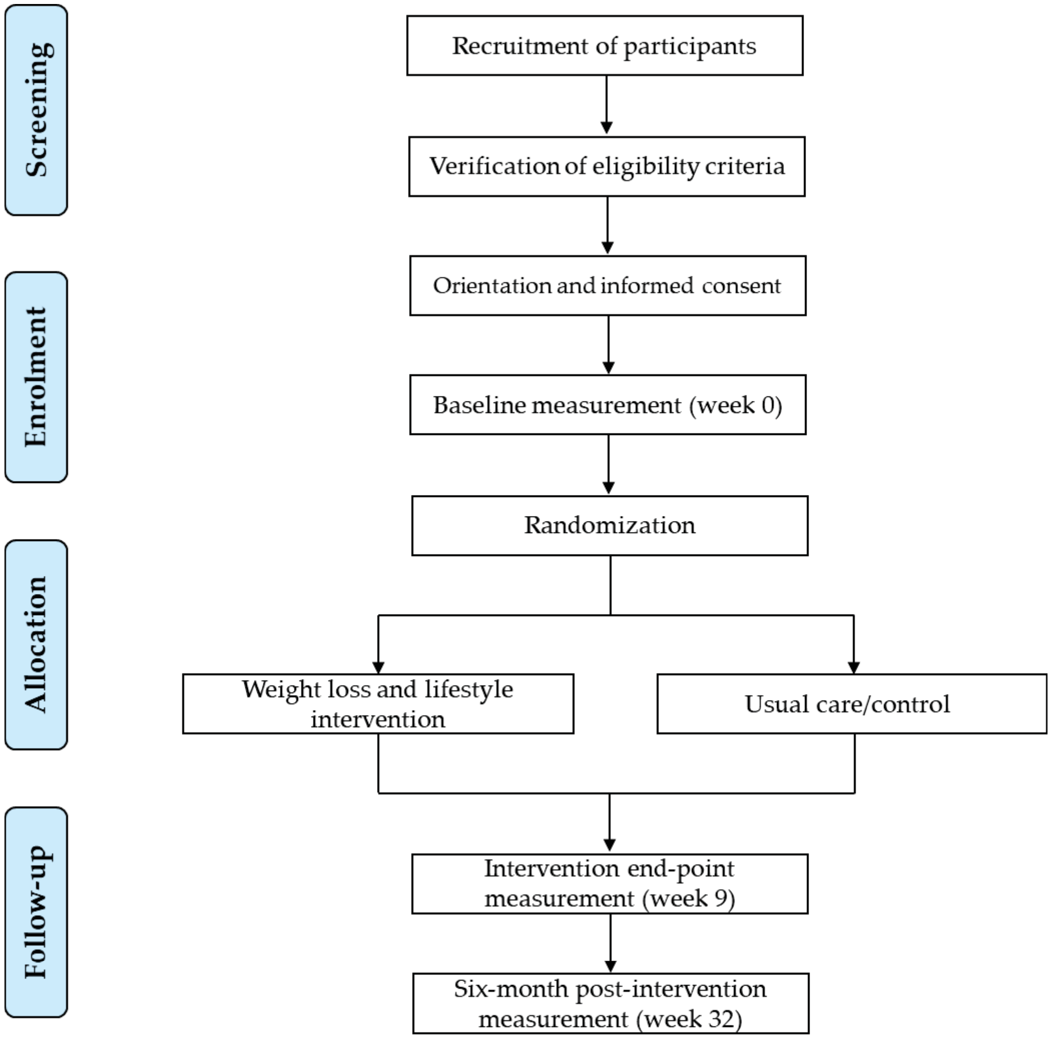

Figure 1. Flow diagram of the INTERAPNEA study participants.

3.4. Assessments

The primary outcome of INTERAPNEA study is the reduction in the number of apnea and/or hypopnea episodes per hour, i.e. AHI, assessed using a full-night ambulatory polysomnography. The main secondary outcomes include other neurophysical and cardiorespiratory polysomnographic outcomes, body weight and composition, physical fitness/cardiorespiratory fitness, and health blood biomarkers. Other variables of interest are subjective measurements of depressive and anxiety symptomatology related to OSA, impaired sleep (i.e. daytime sleepiness, sleep quality, and functional outcomes of OSA), health-related quality of life, and other lifestyle habit measurements (i.e. diet, physical activity, alcohol and tobacco consumption). All outcomes will be measured at baseline (week 0), intervention end-point (week 9), and 6 months post-intervention (week 32).

Assessment of primary and secondary outcomes will be organized and completed over three different days during a one to two-week period:

- Day 1: Potential participants will attend a medical examination and a blood test after a 12-hour overnight fast at the Sleep Unit of Virgen de las Nieves University Hospital.
- Day 2: Eligible participants will complete a full-night in-laboratory polysomnography (PSG; the gold-standard objective testing recommended by the AASM [27]), at the Sleep and Health Promotion Laboratory (CIMCYC). In order to avoid potential CPAP influence on PSG outcomes, participants will be required to withdraw from CPAP during the week prior to the PSG at baseline and follow-ups [28]. Prior to the PSG, participants will also complete a set of questionnaires assessing subjective variables related to sleep, general physical and psychological health, and lifestyle habits including diet, physical exercise, and alcohol and tobacco consumption.
- Day 3: During the third and last assessment day, participants will be required to attend the iMUDS for the measurement of anthropometric parameters, body composition and cardiorespiratory fitness.

Baseline physical activity and sleep habits will also be obtained through a seven-day self-reported daily step log and sleep diary. See Table 2 for study outcomes and measurements.

Table 2. INTERAPNEA study outcomes and measurements.

Variable	Measurement	Assessment
General health history and sociodemographic information	General medical examination (i.e. anamnesis, physical exploration, vital measurements, etc.)	Week 0
	Clinical and socio-demographic interview	Week 0, 9, 32
	Fasting blood test	Week 0, 9, 32
Sleep quality and health-related quality of life		
Sleep habits	Sleep diary	Week 0, 9, 32
Circadian preference/chronotype	Morningness/Eveningness Questionnaire	Week 0, 9, 32
Sleep quality	The Pittsburgh Sleep Quality Index	Week 0, 9, 32
Daytime sleepiness	Epworth Sleepiness Scale	Week 0, 9, 32
Perceived health-related quality of life	Sleep Apnea Quality of Life	Week 0, 9, 32
	Short-Form 36 Health Survey	Week 0, 9, 32
	General Health Questionnaire	Week 0, 9, 32
Objective sleep		
Neurophysiological outcomes	Polysomnography equipment	Week 0, 9, 32
Cardiorespiratory outcomes	Polysomnography equipment	Week 0, 9, 32
Body weight and composition		
BMI and anthropometric measurements	Weight and height measurement, and neck, chest and waist circumferences	Week 0, 9, 32
Body composition	Dual Energy X-ray Absorptiometry	Week 0, 9, 32
Lifestyle habits		
Physical exercise habits	Spring-levered pedometer and daily step logs	Week 0, 9, 32
Dietary habits	Food Behavior Checklist	Week 0, 9, 32
	Mediterranean Diet Adherence Screener	Week 0, 9, 32
Tobacco dependence and consumption	Self-reported tobacco consumption logs	Week 0, 9, 32
	The Fagerstrom Test for Nicotine Dependence	Week 0, 9, 32
Alcohol consumption	Self-reported alcohol consumption logs	Week 0, 9, 32
Physical fitness		
Cardiorespiratory fitness	2-km walk test	Week 0, 9, 32
Subjective physical fitness	International Fitness Scale	Week 0, 9, 32
Daily functioning and mood		
Functional outcomes related to sleepiness	Functional Outcomes of Sleep Questionnaire	Week 0, 9, 32
Subthreshold anxiety symptoms	State-Trait Anxiety Inventory	Week 0, 9, 32
Subthreshold depression symptoms	Beck Depression Inventory-Fast Screen	Week 0, 9, 32
	Inventario de Depresión Estado-Rasgo	Week 0, 9, 32

3.5. Endpoints

Primary endpoint

The primary outcome of the INTERAPNEA study is AHI, defined as the number of apnea (90% or greater drop in airflow for 10 seconds or longer) and hypopneas (30% or greater drop in airflow for 10 seconds or longer associated with ≥ 3% oxygen desaturation or an arousal) episodes per hour of sleep [29].

We will measure this outcome and other neurophysical and cardiorespiratory secondary outcomes through an in-laboratory PSG using SOMNOScreen™ PSG-Tele (SOMNOmedics, GmbH, Randersacker, Germany), or Somté PSG v2 system (Compumedics Limited, Abbotsford, Australia). The recordings will include all recommended physiologic signals such as electroencephalogram (three channels: F4-M1, C4-M1, O2-M1), electrooculogram (two channels: E1 and E2), electromyogram (two channels: submental and anterior tibialis muscles), and electrocardiogram (two channels). Cardiorespiratory measurements will include oral and nasal airflow (triple thermistor), oxyhaemoglobin saturation (SpO2) and pulse-rate (pulse oximeter), respiratory effort (chest and abdomen bands), and body position (sensor). All electrodes will be placed in accordance with the international 10-20 system [30], and recordings will be automatically and manually scored in 30-second epochs [31] by trained physicians using DOMINO (v2.7, SOMNOmedics, GmbH, Randersacker, Germany), or ProFusion PSG 3 (v3.3, Compumedics Limited, Abbotsford, Australia) associated computer software. All parameters, settings, filters, technical specifications, sleep stage scoring and event scoring will be performed in accordance with the AASM Manual for the Scoring of Sleep and Associated Events [29].

We will also specifically analyze AHI in rapid eye movement (REM) and non-REM sleep stages (N1, N2, and N3). Although it has been shown that REM apnea episodes may yield to more adverse cardiovascular consequences than non-REM obstructions [32], previous similar RCTs have rarely included the reduction in AHI differentiated by these sleep stages [1].

Secondary Endpoints

Neurophysical and cardiorespiratory polysomnographic outcomes

Secondary polysomnographic outcomes related to OSA, measured by PSG as above mentioned, are oxygen desaturation index (number of oxygen desaturation $\geq$ 3% per hour), SpO2 mean (average of oxygen saturation), SpO2 nadir (minimum oxygen saturation), sleep efficiency (total sleep time/total time in bed), sleep latency, wake after sleep onset, REM sleep stage, and nonREM sleep stages (N1, N2, and N3).

Physical fitness

Cardiorespiratory fitness will be measured through a 2-km walking test, which has been widely used and validated for accurate estimation of maximum oxygen uptake

(VO_{2max}) [33]. Participants will be required to walk over a marked 2 km track on a firm surface wearing a heart rate monitor (Polar RS800cx, Polar Electro, Kempele, Finland). Walking time and heart rate (HR) will be recorded at the end of the test. The maximal aerobic power will then be calculated considering age, BMI, performance time, and HR with the following formula VO_{2max} (ml/min/kg) = 116.2 - 2.98 * walking time (sec) - 0.11 * HR -0.14 * age - 0.39 * BMI [34]. Participant's scores will be obtained and placed within a fitness category. Subjective physical fitness will also be measured using the International Fitness Scale (IFIS) [35].

Body weight and composition

Body weight and height will be measured using a calibrated scale and stadiometer (model 799, Electronic Column Scale, Hamburg, Germany) with participants wearing undergarments. Neck, chest and waist circumferences will also be measured following standard procedures recommended by the International Society for the Advancement of Kinanthropometry (ISAK) [36]. Body composition measurements including fat mass (kg), fat free mass, lean mass (kg), visceral adipose tissue (kg), and bone mineral density (g/cm2) will be obtained through a full-body dual energy X-ray absorptiometry (DXA) scanner (Discovery Wi, Hologic, Inc., Bedford, MA, USA). An additional two-dimensional lumbar spine DXA scanner will also be performed in order to obtain the trabecular bone score (TBS); an index of bone microarchitecture that consistently and significantly predicts bone fracture risk. Quality controls, positioning of participants and analyses of results will be performed following the manufacturer's recommendations. Automatic delineation of anatomic regions will be performed using APEX 4.0.2. software.

Blood biomarkers

Blood biomarkers will include glucose metabolism (glucose [mg/dl], insulin [IU/ml] and insulin resistance as indicated by the homeostasis model assessment of insulin resistance [HOMA-IR] index), lipid metabolism (total cholesterol [mg/dl], high-density lipoprotein cholesterol [HDL-C; mg/dl], low-density lipoprotein cholesterol [LDL-C; mg/dl], triglycerides [mg/dl], and apolipoproteins A1 and B [mg/dl]), and liver function (aspartate aminotransferase [AST; IU/l], alanine aminotransferase [ALT; IU/l], γ-glutamyltransferase [γ-GT], and fatty liver index [FLI]). These variables will be measured through blood samples obtained from participants' antecubital vein in a supine position

during the morning in a fasting state. Samples will be collected into prechilled ethylene diamine tetra-acetic acid-containing tubes (Vacutainer SST, Becton Dickinson, Plymouth, UK) and immediately centrifuged (i.e. 15 minutes at 3,000 rpm), aliquoted and stored at −80°C for further plasma analysis. Glucose levels will be measured by spectrophotometric techniques (AU5800, Beckman Coulter, Brea, California, USA). Insulin will be assessed by chemiluminescence immunoassay with paramagnetic particles (UniCel DxI 800, Beckman Coulter, Brea, California, USA). HOMA-IR will be calculated as fasting glucose (mmol/l) times the level of fasting insulin (UU/ml) divided by 22.5. Total cholesterol, HDL-C, triglycerides, and apolipoproteins A1 and B will be automatically evaluated by spectrophotometric techniques (AU5800, Beckman Coulter, Brea, California, USA). LDL-C will be calculated as the level of total cholesterol minus the level of HDL-C minus 0.45 times the level of triglycerides. AST, ALT and γ-GT will be calculated by absorption spectrophotometric techniques (Beckman Coulter, Brea, California, USA). FLI will be calculated with the formula FLI = ((e 0.953·loge (Triglycerides) + 0.139·BMI + 0.718·loge (γ-GT) + 0.053·Waist Circumference - 15.745)) / ((1 + e 0.953·loge (Triglycerides) + 0.139·BMI + 0.718·loge (γ-GT) + 0.053·Waist Circumference - 15.745)) ·100.

Systolic blood pressure (mm Hg), diastolic blood pressure (mm Hg) and mean blood pressure (mm Hg) will also be considered as cardiometabolic risk outcomes. Blood pressure will be measured with an ambulatory blood pressure monitor (Omron M3 Blood Pressure Monitor, OMRON Healthcare, Hoofddorp, Netherlands) in a sitting position after at least 5 minutes of rest. The mean of two measurements will be recorded. Mean blood pressure will be calculated at each reading as one third of systolic pressure plus two thirds of diastolic pressure.

Lifestyle habits: Dietary habits, physical activity, smoking, and alcohol intake

Participants' dietary habits will be evaluated using the validated 14-item Mediterranean diet screener (MEDAS), which evaluates food consumption frequency (12 items) and characteristic dietary habits of the Mediterranean diet (2 items) [37]. MEDAS items are scored with 0 or 1, the total score ranging from 0 to 14 points. The 22-item Food Behavior Checklist (FBC) will also be used to assess participants' food intake and habits [38]. FBC comprises seven subscales including consumption of fruit and vegetables (9 items), diet quality (4 items), fast food (3 items), dairy/calcium (2 items), sweetened beverages (2 items), meat (1 item) and food security (1 item). This instrument has been

shown to be effective at evaluating dietary behavior changes after nutrition education interventions promoting healthy diets [38].

Physical activity will be measured using daily step logs recorded by participants with a spring-levered pedometer (OcioDual, Alicante, Spain). Participants will be required to wear the pedometer all day and register the number of steps achieved per day in a seven-day step log. The average steps per day will then be calculated at baseline and follow-ups.

Regarding the remaining lifestyle habits, smoking and alcohol intake will be measured at baseline and follow-ups using seven-day self-reported tobacco and alcohol consumption logs. Recordings will include number of cigarettes/alcoholic units consumed per day, cigarette brand/type of alcoholic drink, time, situation, and perceived pleasure (from 0 to 10). The validated form of the Fagerström Test for Nicotine Dependence (FTND) [39] will also be used to assess participants' nicotine dependence in all assessments.

Daily functioning and mood

OSA impact on daily functioning and mood will be measured through validated versions of the Functional Outcomes of Sleep Questionnaire (FOSQ) [40], Beck Depression Inventory-Fast Screen (BDI-FS) [41], State-Trait Anxiety Inventory (STAI) [42] and Inventario de Depresión Estado-Rasgo (IDER) [43]. It has been shown that impaired daytime functioning and depressive and anxiety symptoms are very common in patients with OSA, being higher in patients with more severe OSA and a greater BMI [44]. Hence, participants will complete a set of questionnaires on these symptoms not only to measure inclusion/exclusion criteria but also to analyze potential changes in daily functioning and mood driven by the INTERAPNEA study intervention.

Daytime sleepiness, sleep quality and health-related quality of life

The Epworth Sleepiness Scale (ESS) [45], an 8-item Likert-based scale, will be used to obtain subjective measurements of participant's daytime sleepiness. Excessive daytime sleepiness is the most common consequence of OSA due to sleep fragmentation and deprivation, and one of the mediating factors for other OSA outcomes such as daily functioning, social and occupational disturbances [6-8].

As sleep quality is also closely related to daily functioning, mood and, thus, participant's general quality of life and well-being [46,47], we will measure potential

benefits of the INTERAPNEA study intervention on these variables through the validated versions of the Pittsburgh Sleep Quality Index (PSQI) [48], Sleep Apnea Quality of Life (SAQLI) [49], Short-Form 36 Health Survey (SF-36) [50], and General Health Questionnaire (GHQ-28) [51].

In addition, we will subjectively measure circadian preference or individual's chronotype using the validated reduced 5-item version of the Morningness-Eveningness Questionnaire (MEQ) [52], an outcome which has been closely related to age, BMI and, in turn, OSA [53]. Evidence suggests that evening-type chronotype may be highly associated with greater unhealthy eating behaviors, sleep disruption, poor sleep quality and mood disturbances, all playing a part in the development and severity of OSA [53,54].

3.6. Weight Loss and Lifestyle Intervention Arm

The design, implementation and evaluation of the INTERAPNEA study intervention components and characteristics are based on results of previous epidemiological and clinical research [1,10] as well as on international evidenced-based clinical practice guidelines for the management of OSA [14,19]. Considering our previous research [1] and with the final aim of the intervention being adaptable to actual primary health-care settings, the intervention will last eight weeks, and will be composed of five different modules (i) nutritional behavior change, (ii) moderate aerobic exercise, (iii) smoking reduction and cessation, (iv) alcohol intake avoidance, and (v) sleep hygiene (see Table 3). Each component will include group-based weekly sessions of 60-90 minutes lead and supervised by a trained professional in the field (i.e. human nutrition and dietetics, physical activity and sport sciences, and psychology).

The key-factor of this interdisciplinary intervention will be the use of the Transtheoretical Model of Health Behavior Change (TM) by Prochaska and Diclemente [55]. This well-evidenced model of behavior change is based on integrating different intervention theories into an interventional approach that considers different stages, processes and principles of change with the premise of establishing sustainable health-related behaviors or habits [55]. Consciousness raising, self-reevaluation, counterconditioning, stimulus control, contingency management, goal-setting, self-monitoring, self-efficacy, and decisional balance are some of the processes and principles of change addressed by this theory and, therefore, included in the five different INTERAPNEA intervention components. Physical and dietary interventions for weight loss using strategies of TM and, thus, psychological support, have been shown to be more

effective than other approaches in overweight and obese patients [56] and, specifically, in those with OSA [1].

Table 3. Description and timing of the INTERAPNEA intervention modules and components.

<table>
<tr><th>Module</th><th>Objectives/Description</th><th>Number of sessions</th><th>Frequency of sessions</th><th>General behavioral change techniques</th></tr>
<tr><td>Nutritional behavior change</td><td>Nutrition education and dietary patterns change</td><td>8</td><td>Once a week</td><td rowspan="5">• Motivation and preparation for action
• Goal-setting and action-planning
• Self-monitoring and functional behavioral analysis
• Review of behavioral goals, action plans, and adherence
• Problem solving and social skills
• Self-efficacy, maintenance, and relapse prevention</td></tr>
<tr><td>Physical exercise</td><td>Supervised moderate aerobic exercise and increase daily steps by 15% each week</td><td>8</td><td>Once a week</td></tr>
<tr><td>Sleep hygiene</td><td>Change of inappropriate sleep habits: insufficient sleep, consumption of coffee, alcohol and tobacco, and inappropriate sleep schedule and environment</td><td>4</td><td>Once each two weeks</td></tr>
<tr><td>Tobacco cessation</td><td>Nicotine and cigarette fading: reduction of nicotine and number of cigarettes by 30% each week</td><td>8</td><td>Once a week</td></tr>
<tr><td>Alcohol avoidance</td><td>Alcohol consumption fading: reduction of alcohol consumption by 30% each week</td><td>4</td><td>Once each two weeks</td></tr>
</table>

Nutritional behavior change

Diet quality and dietary patterns have been shown to be closely related to biologic pathways involved in chronic disease etiology [57] and, specifically, to sleep disruption, fragmentation and poor sleep quality found in OSA [58]. Recent studies have shown that high-fat intake is associated with lower sleep efficiency and REM sleep and higher arousal index, whereas high-carbohydrate intake may improve sleep duration and architecture by producing reductions in sleep-onset latency and higher proportions of REM sleep [58]. Regarding intake of micronutrients, vitamin D — which has been associated with insulin resistance in OSA [59] — and magnesium deficiencies have also been related to shorter sleep duration, poorer sleep quality and higher daytime sleepiness [60,61]. Therefore, dietary components including milk, fish, fruit and vegetables may yield beneficial effects on sleep and, in turn, OSA [58].

Furthermore, evidence suggests that sleep disturbances occurred in OSA, in turn, have adverse consequences on calorie intake and expenditure, exposing therefore a two-way relationship between dietary habits and sleep [62]. Empirical studies have revealed that sleep fragmentation and deprivation are related to higher energy intake of unhealthy

food due to increased hunger, food craving, food reward and portion size selection [63-65]. Neurocognitive impairments found in patients with OSA such as attention and episodic memory deficits [66] have also been associated with higher intake of saturated fats, loss of control over food intake, and thus uncontrolled eating [67].

Hence, The INTERAPNEA intervention includes a nutrition module comprising eight sessions (once a week) of 60-90 minutes in a group format addressing dietary patterns using integrated techniques of nutrition education and behavioral change such as specific goal-setting, cognitive restructuring, stimulus control, progressive muscle relaxation, social skills and assertiveness, and problem solving skills. The nutrition education is based on the World Health Organization (WHO) latest recommendations on food intake and healthy diet (see Table 4 for detailed topics) and each session will follow a three-part format: i) Brief review of previous session and participant's adherence to recommendations; ii) Development of the main nutrition education component of each session using an interactive group discussion layout; iii) Resolution of participant's questions and/or concerns, and setting of specific goals. No specific or individualized diet will be indicated to participants.

Table 4. Description of nutrition education per session.

Session	Nutrition education topics
Session 1	Adverse consequences of obesity, importance of healthy nutrition and body composition on health, and positive effects of changes in nutrition.
Session 2	Maintenance of a healthy nutrition based on the Harvard Plate model: increasing consumption of healthy food (vegetables, fruits, legumes, nuts, extra olive virgin oil, fish and shellfish, white meat, eggs and herbs) and decreasing consumption of unhealthy food (ultra-processed foods, excessive salt consumption, processed meats, red meat, alcohol, and high-calorie foods and beverages).
Session 3	Food myths and health risks of miracle diets.
Session 4	Strategies to improve satiety and decrease appetite: decreasing dishes dietary energy density, choosing food with low dietary energy density, managing dietary fat intake, including enough fibre and protein quantity, limiting sugar and ultra-processed foods, choosing water and low-calorie beverages, and managing portion sizes.
Session 5	Healthy breakfast and snacks: avoiding unhealthy breakfast and snacks and turning them into healthy.
Session 6	Healthy cooking, food purchase and choices when eating out.
Session 7	How to read nutritional labels of food and distinguish between healthy and unhealthy food.
Session 8	Nutritional strategies to improve sleep quality.

Physical exercise

Physical exercise has been shown to be effective in enhancing OSA outcomes and health-related consequences [1,68,69]. Due to the close association between OSA and obesity, a significant and sustainable increase of physical activity could lead to a reduced body weight and, in turn, improvement of the upper airway structure, function, and resting lung volume [12]. Furthermore, physical exercise could also assist the balance of energy intake and expenditure [70], and improve respiratory center modulation through a reduction of the high leptin and ghrelin hormone levels, abnormalities linked to excessive energy intake and found in OSA patients [71]. Yet, some research found that exercise benefits on OSA were independent to weight loss [69], suggesting that there are other related mechanisms potentially leading to OSA enhancement such as the increase of sleep efficiency and slow wave sleep [72], and a decrease of fluid accumulation implicated in the upper airway collapse [73], both due to the direct association between physical exercise and sleep.

Therefore, the INTERAPNEA study will include an eight-week physical exercise program consisting of weekly 60 min-sessions of supervised moderate intensity aerobic exercise (i.e. 55-65% of the heart rate reserve) and individualized goal-setting of increasing daily steps per week. Previous studies have emphasized that walking may be the exercise modality, achieving higher levels of weight loss and increased cardiorespiratory fitness in adults with obesity and CPAP-treated OSA [74]. Thus, in the weekly supervised training sessions, participants will be required to walk at a moderate intensity for 60 minutes wearing a heart rate monitor in order to train themselves to walk at that intensity during the rest of the week. With respect to goal-setting, they will be advised to increase their daily steps by 15% per week, based on their daily steps logs.

Sleep hygiene

Sleep hygiene refers to the practice of certain behaviors that facilitate sleep onset and maintenance (e.g. regular sleep schedule, appropriate sleep environment, exercise-training, and healthy nutrition), and avoidance of habits interfering with sleep (e.g. daytime napping, smoking, alcohol intake, and use of hypnotics) [75]. Patients with OSA frequently exhibit poor sleep hygiene including voluntary sleep restriction, irregular sleep schedule, inappropriate sleep environment, and excessive consumption of alcohol, nicotine and/or caffeine [76]. Accordingly, previous studies have supported the inclusion

of this component on the treatment of OSA as effective in improving sleep quantity and efficiency, and therefore daytime sleepiness [1,77,78].

The INTERAPNEA study intervention will include a sleep hygiene module comprising 60 min sessions supervised by a psychologist specialized in the evaluation and treatment of sleep disorders. As most sleep hygiene topics will be covered in simultaneous modules, there will be four sessions distributed over the eight weeks of the intervention, consisting of sleep hygiene education on causes of sleep disturbances and mistaken sleep related knowledge (see Table 5). Sessions will also be based on treating those frequent inadequate sleep habits found in patients with OSA, i.e. sleep restriction, irregular schedule and inappropriate sleep environment.

Table 5. Summary of components of the sleep hygiene module per session.

Session	Intervention objectives/components
Session 1	• Goal-setting and action-planning: Objective specification and commitment<br>• Self-monitoring: Sleep diary<br>• Psychoeducation: What is sleep hygiene?<br>• Cognitive restructuring: Irrational, false or inaccurate beliefs about sleep<br>• Breathing and relaxation techniques: Diaphragmatic breathing and progressive muscle relaxation
Session 2	• Review of behavioral goals, action plans, and compliance (participant's homework)<br>• Self-monitoring: Sleep diary<br>• Psychoeducation: Voluntary sleep restriction; coffee, alcohol and tobacco consumption before sleep; and irregular sleep schedule and environment.<br>• Cognitive restructuring: Irrational, false or inaccurate beliefs about sleep<br>• Review of diaphragmatic breathing and progressive muscle relaxation
Session 3	• Review of behavioral goals, action plans, and compliance (participant's homework)<br>• Self-monitoring: Sleep diary<br>• Vicarious and self-reinforcement: Changes in sleep habits achieved and benefits<br>• Stimulus control and bedtime restriction<br>• Review of diaphragmatic breathing and progressive muscle relaxation
Session 4	• Review of behavioral goals, action plans, and compliance (participant's homework)<br>• Self-monitoring: Sleep diary<br>• Vicarious and self-reinforcement: Changes in sleep habits achieved and benefits<br>• Review of all intervention components: Main factors of sleep hygiene, diaphragmatic breathing and progressive muscle relaxation, stimulus control and bedtime restriction<br>• Maintenance and relapse prevention: Analysis of high risk situations for unhygienic sleep.

Reduction and avoidance of tobacco consumption

Smoking has been related to the worsening of OSA via different mechanisms such as changes in sleep architecture and increase of arousal threshold from sleep, reduction of the upper airway muscle tones and neural reflexes, and increased inflammation of the upper airway, all due to nicotine and smoke inhalation [21]. In turn, OSA could also be a predisposing factor for smoking addiction, nicotine acting as a reward or self-medication related to the depressive and anxiety symptoms commonly found in OSA [79]. Although

this association has been well elucidated, there are no empirical evidences of the potential beneficial effects of smoking cessation on OSA as, surpringsily, there are no studies focusing on active smoking cessation interventions in patients with this condition [1].

Therefore, we will include a smoking reduction and avoidance module in the INTERAPNEA study intervention. Participants with smoking addiction and willing to quit will be required to attend a weekly 60-90 minute session over eight weeks lead by two clinical psychologists. The specific intervention is based on the group behavior therapy for smoking cessation by Becoña et al. [80]. This therapy seeks the progressive reduction of tobacco consumption through the use of nicotine and cigarette fading [81], and behavior-change techniques such as information on smoking, self-monitoring, stimulus control, avoidance of withdrawal symptoms, and relapse prevention (see Table 6). Nicotine and cigarette fading has been shown to be the most effective method to reduce and stop smoking with abstinence rates of 86% at the end of treatment and nearly 60% at a 12 month follow-up [82].

Thus, participants will be mainly required to keep a daily record of number of cigarettes smoked and triggers for smoking (self-monitoring), change the type of cigarette smoked to a lesser nicotine content brand each week (30%, 60% and 90% nicotine reductions from baseline), reduce the number of cigarettes smoked by 30% weekly, and avoid smoking in three different situations per week (stimulus control). Through the sessions, other behavior change techniques will be used such as discussion of health consequences of smoking and quitting (motivation), muscle and cognitive relaxation techniques to address withdrawal symptoms, and identification of high-risk situations for smoking and problem-solving skills (relapse prevention).

Table 6. Summary of components of the smoking cessation module per session.

Session	Intervention objectives/components
Session 1	• Goal-setting and action-planning: Objective specification and commitment<br>• Self-monitoring: Cigarette consumption logs<br>• Psychoeducation: Cigarette components and smoking consequences<br>• Cognitive restructuring: Irrational, false or inaccurate beliefs about smoking cessation<br>• Stimulus control: Reduction strategies<br>• Nicotine and cigarette fading: Reduction of nicotine/number of cigarettes by 30% from baseline<br>• Breathing and relaxation techniques: Diaphragmatic breathing and progressive muscle relaxation
Session 2	• Review of behavioral goals, action plans, and compliance (participant's homework)<br>• Self-monitoring: Cigarette consumption logs<br>• Cognitive restructuring: Irrational, false or inaccurate beliefs about smoking cessation<br>• Stimulus control: Smoking avoidance in three different situations and other reduction strategies<br>• Nicotine and cigarette fading: Reduction of nicotine/number of cigarettes by 30% from week 1<br>• Maintenance and relapse prevention: Avoidance of withdrawal symptoms<br>• Review of diaphragmatic breathing and progressive muscle relaxation
Session 3	• Review of behavioral goals, action plans, and compliance (participant's homework)<br>• Self-monitoring: Cigarette consumption logs<br>• Vicarious and self-reinforcement: Changes in smoking achieved and benefits<br>• Stimulus control: Smoking avoidance in six different situations and other reduction strategies<br>• Nicotine and cigarette fading: Reduction of nicotine/number of cigarettes by 30% from week 2<br>• Maintenance and relapse prevention: Avoidance of withdrawal symptoms<br>• Review of diaphragmatic breathing and progressive muscle relaxation
Session 4	• Review of behavioral goals, action plans, and compliance (participant's homework)<br>• Self-monitoring: Cigarette consumption logs<br>• Vicarious and self-reinforcement: Changes in smoking achieved and benefits<br>• Stimulus control: Smoking avoidance in nine different situations and other reduction strategies<br>• Nicotine and cigarette fading: Reduction of nicotine/number of cigarettes by 30% from week 3<br>• Maintenance and relapse prevention: Avoidance of withdrawal symptoms<br>• Review of diaphragmatic breathing and progressive muscle relaxation
Session 5	• Review of behavioral goals, action plans, and compliance (participant's homework)<br>• Self-monitoring: Cigarette consumption logs<br>• Vicarious and self-reinforcement: Changes in smoking achieved and benefits<br>• Abstinence planning: Setting the day when abstinence starts<br>• Problem solving and social skills: High risk situations for smoking and alternative behaviors<br>• Maintenance and relapse prevention: Avoidance of withdrawal symptoms<br>• Review of diaphragmatic breathing and progressive muscle relaxation
Sessions 6, 7, 8	• Review of behavioral goals, action plans, and compliance (participant's homework)<br>• Self-monitoring: Cigarette consumption logs/Review of abstinence<br>• Abstinence planning: Setting the day when abstinence starts<br>• Vicarious and self-reinforcement: Changes in smoking achieved and benefits<br>• Problem solving and social skills: High risk situations for smoking and alternative behaviors<br>• Review of diaphragmatic breathing and progressive muscle relaxation<br>• Maintenance and relapse prevention: Difference between lapse and relapse

Reduction and avoidance of alcohol intake

Alcohol intake has also been related to the development and worsening of OSA not only for its direct and indirect effects on weight gain but also due to its negative impact on breathing parameters during sleep [20]. Recent meta-analyses on alcohol and risk of sleep apnea emphasized that alcohol intake increases the risk of breathing cessation

episodes by 25%, thus increasing AHI and reducing mean SaO2 during sleep [83]. Potential explanations of these adverse consequences may be the alcohol-related hypotonia of oropharyngeal muscles during sleep, and depression of the arousal response to asphyxia, both caused by the alcohol depressant effects on the central nervous system [84].

Therefore, the INTERAPNEA study intervention will include an alcohol intake reduction and avoidance module supervised by two clinical psychologists. As we will be treating excessive alcohol intake as opposed to alcohol dependence, this module will last eight weeks comprising fortnightly sessions of 60 minutes. Similar to the smoking cessation module, the main content of this specific component is the progressive reduction of alcohol intake in those participants with no alcohol addiction but excessive consumption (see Table 7). Thus, participants will be indicated to reduce the number of units of alcohol consumed per day/week by 30% each week, keeping a log of alcohol-consumption per day including units of alcohol consumed and triggers of consumption. During the sessions, participants will receive detailed information of alcohol general and specific to OSA health-related consequences. Furthermore, behavior change techniques such as stimulus control, muscle and cognitive relaxation and problem-solving skills related to alcohol consumption will be used.

Table 7. Summary of components of the alcohol avoidance module per session.

Session	Intervention objectives/components
Session 1	• Goal-setting and action-planning: Objective specification and commitment<br>• Self-monitoring: Alcohol-consumption logs<br>• Psychoeducation: Alcohol consumption and adverse consequences for obstructive sleep apnea<br>• Cognitive restructuring: Irrational, false or inaccurate beliefs about alcohol consumption<br>• Alcohol fading: Reduction of alcohol consumption/number of alcoholic drinks by 30% from baseline<br>• Breathing and relaxation techniques: Diaphragmatic breathing and progressive muscle relaxation
Session 2	• Review of behavioral goals, action plans, and compliance (participant's homework)<br>• Self-monitoring: Alcohol-consumption logs<br>• Cognitive restructuring: Irrational, false or inaccurate beliefs about smoking cessation<br>• Alcohol fading: Reduction of alcohol consumption/number of alcoholic drinks by 30% from week 1<br>• Stimulus control: Reduction/abstinence strategies<br>• Review of diaphragmatic breathing and progressive muscle relaxation
Session 3	• Review of behavioral goals, action plans, and compliance (participant's homework)<br>• Self-monitoring: Alcohol-consumption logs<br>• Vicarious and self-reinforcement: Changes in alcohol consumption achieved and benefits<br>• Abstinence planning: Setting the day when abstinence starts<br>• Stimulus control: Reduction/abstinence strategies<br>• Review of diaphragmatic breathing and progressive muscle relaxation

Session 4	• Review of behavioral goals, action plans, and compliance (participant's homework)<br>• Self-monitoring: Alcohol-consumption logs<br>• Vicarious and self-reinforcement: Changes in alcohol consumption achieved and benefits<br>• Problem solving and social skills: High risk situations for drinking alcohol and alternative behaviors<br>• Review of all intervention components: diaphragmatic breathing and progressive muscle relaxation, stimulus control<br>• Maintenance and relapse prevention: Difference between lapse and relapse	

3.7. Assessment of Intervention Compliance and Integrity

Integrity of the intervention and treatment fidelity will be evaluated and ensured through the design and implementation of different strategies of process assessment, monitoring and enhancement in order to guarantee internal and external validity of the trial [85].

Firstly, regarding the study design and provider of intervention training, we developed a comprehensive hand-book for the qualified INTERAPNEA study intervention providers/professionals/training personnel of each module (nutrition, physical exercise, sleep hygiene, and tobacco and alcohol consumption). Each intervention manual identifies the theoretical model of the intervention and provides detailed descriptions of session objectives, treatment guidelines in accordance with each objective (i.e. contents, tasks and activities, recommendations, and timing), participant's homework, and material needed for each session. We will also provide each participant with an adapted patient-handbook for each intervention component including descriptions of sessions, and work and logging sheets.

Secondly, we will ensure fidelity in the treatment delivery, receipt, and enactment through the use of these intervention protocols/manuals and monitoring of the implementation. Regarding the treatment delivery, the standardization of the intervention will support the protocol adherence of providers and the treatment differentiation (i.e. the delivery of the target treatment and no other). Furthermore, we will include a check-list for provider's self-report concerning the achievement of session objectives. With respect to the treatment receipt and enactment, fidelity will be assessed and confirmed through different strategies such as the structuring of the intervention around achievement-based objectives, collecting and reviewing of participants self-monitored data (daily steps log, sleep diaries, alcohol and tobacco consumption records), and information delivery in different formats (e.g. written in the handbooks, and verbal and visual in the sessions).

Finally, apart from the above mention strategies, we will consider complementary approaches in order to reduce participant drop-out rates and increase adherence such as

prevention of commitments or vacation periods, use of well-equipped and conditioned facilities, and supervision by a qualified and certified pair of providers in each session motivating and supporting participants. Participants' attendance to each intervention session will be recorded by providers, and phone-calls will be made to assess causes of absence and determine further participation in the intervention.

3.7. Usual Care Arm

Participants with moderate-severe OSA randomly assigned to the usual care group (control) will receive, apart from CPAP treatment, a 30 min session of general advice on weight loss and lifestyle change from a sleep disordered-breathing specialist. Informative leaflets describing the positive effects of healthy nutrition, physical activity, sleep hygiene and tobacco and alcohol avoidance for OSA will also be provided to these participants. Additionally, the opportunity to receive the INTERAPNEA study intervention will be offered to this control group after the six month follow-up.

3.8. Serious Adverse Events

Serious adverse events occurring to participants from baseline to intervention endpoint and/or 6-month follow-up, related or unrelated to the study intervention or participation, will be collected and registered over the course of the trial by the study personnel. These are defined as events that lead to death, life-threatening illness, permanent impairment, or hospitalization with a serious health condition.

3.9. Power and Sample Size

The sample size calculation and power of the study are based on the data of previously reported studies contrasted, combined, and synthesized in our recent systematic review and meta-analysis [1]. We considered following the formula $\boldsymbol{n} = \frac{2(Z_\alpha + Z_{1-\beta})^2\sigma^2}{\Delta^2}$, where n is the required sample size, $Z\alpha$ and $Z(1\text{-}\beta)$ are the constants set by convention according to the accepted α error and power of the study, respectively, σ is the estimated standard deviation, and Δ is the expected effect size. Therefore, we expect to detect an effect size of -8.36 on AHI (pooled raw mean difference of previous trials) [1], considering a type 1 error/α error of 5%, and a statistical power of 90%. Regarding the estimated AHI variability, we established an σ of 11.98, considering the AHI pooled standard deviation of all independent samples included in our previous research [1]. As a result, the expected sample size is of ≈ 35 participants per arm of our

controlled clinical trial. However, assuming a maximum of a 17.25% drop-out rate (based on the average drop-out rate of previous studies [1]), we decided to recruit a total sample size of ≈ 42 participants for each study group. Thus, a total of ≈ 84 patients with moderate to severe OSA will be enrolled in the INTERAPNEA study. For practical and feasibility reasons, and based on our previous experience [86,87], the study will be conducted in sets of a maximum of 30 persons.

3.10. Statistical Analysis

We will perform descriptive and exploratory preliminary analyses of all the study variables to reveal violations of statistical assumptions, distributions, imbalances between the study groups, associations between study variables, covariates/confounders, amount of missing data, and drop-out patterns.

Intervention effects on primary and secondary outcomes will be assessed based on linear mixed-effects models using the package *lme4* [88] from the R statistical program, with individual measures of growth being modeled as the function of randomly assigned group, assessment time, and the interaction between group and time (fixed variables). If required, effects of set of participants will also be included in the model as a random cluster factor. Estimations will be performed using the restricted maximum-likelihood method, including an unstructured covariance matrix to adjust for within-participant clustering resulting from the repeated-measures design. The model will assume that missing values are missing-at-random. Nevertheless, attrition propensity will be calculated using a logistic model predicting attrition with baseline values of set of participants, allocation group, OSA severity, age and BMI [89].

All estimations and analyses will be performed with an intention-to-treat approach (including all participants as originally allocated after randomization) and an additional per-protocol approach restricted to participants with a CPAP usage greater or equal to 4 hours per night on 70% of nights and, regarding the intervention group, at least 80% of attendance rate at intervention sessions.

4. ORGANIZATION

The Sleep and Health Promotion Laboratory of the Mind, Brain and Behavior Research Center (CIMCYC) from the University of Granada (Granada, Spain) was responsible for the study design and organization, participant recruitment process, data collection and management, randomization and participant allocation, trial monitoring, and reporting of the study process and results. Participants previously diagnosed with moderate-to-severe OSA and potentially meeting the inclusion criteria were recruited from the collaborating sleep-disordered breathing unit of the Virgen de las Nieves University Hospital (Granada, Spain). Data collection at baseline and intervention endpoint, as well as implementation of the weight loss and lifestyle intervention, was performed in two different settings of the University of Granada (Granada, Spain): the Sleep and Health Promotion Laboratory (CIMCYC) and the Sport and Health University Research Institute (iMUDS). General and specific OSA standard care (i.e. continuous positive airway pressure [CPAP]) of all participants enrolled in the trial continued being provided by their primary care team from the Virgen de las Nieves University Hospital.

5. ETHICAL ISSUES

5.1. Institutional Ethics Committee Approval

This study conforms to the last revised Ethical Principles for Medical Research Involving Human Subjects comprised in the Declaration of Helsinki and written approvals were obtained from all relevant Research Ethics Committees (i.e. Institutional Review Board) before the recruitment of participants. Specifically, approval were obtained from the Research Ethics Committees of: (a) University of Granada (Granada, Spain); (b) Virgen de las Nieves University Hospital (Granada, Spain); and (c) Junta de Andalucía (Spain) (0770-N-19).

5.2. Participant Consent

All participants will receive accurate information on the study assessments and intervention, and written informed consent from each participant will be obtained prior to any data collection. Signed copies of the Consent Form and a Patient Information Sheet will be given to participants. Those participants unable or not willing to give informed consent will not be eligible for enrollment in this study.

The Patient Information Sheet will also clearly state that participants have the right to withdraw from the study at any time without prejudice or explanation.

5.3. Confidentiality and Privacy

The confidentiality and privacy of all participants will be cautiously respected and ensured throughout the conduct of the study. All data will only be identified by a unique identification number/code given to each participant at the beginning of the trial; all data treatment and analyses being correspondingly blinded.

5.4. Provision of the Trial Intervention to All Participants

The interdisciplinary weight loss and lifestyle intervention will be offered to all participants randomly assigned to the usual-care/control group (i.e. CPAP alone) at the conclusion of the trial. This information is included in the patient information and consent forms.

6. REFERENCES

1. Carneiro-Barrera, A.; Díaz-Román, A.; Guillén-Riquelme, A.; Buela-Casal, G. Weight loss and lifestyle interventions for obstructive sleep apnea in adults: Systematic review and meta-analysis. Obes. Rev. 2019, 20, 750–762. doi:10.1111/obr.12824
2. Jordan, A.S.; McSharry, D.G.; Malhotra, A. Adult obstructive sleep apnea. Lancet 2014, 383, 736–747. doi:10.1016/S0140-6736(13)60734-5
3. Fu, Y.; Xia, Y.; Yi, H.; Xu, H.; Guan, J.; Yin, S. Meta-analysis of all-cause and cardiovascular mortality in obstructive sleep apnea with or without continuous positive airway pressure treatment. Sleep Breath. 2017, 21, 181–189. doi:10.1007/s11325-016-1393-1
4. Hoyos, C.M.; Drager, L.F.; Patel, S.R. OSA and cardiometabolic risk: What's the bottom line? Respirology 2017, 22, 420–429. doi:10.1111/resp.12984
5. Blackwell, T.; Yaffe, K.; Laffan, A.; Redline, S.; Ancoli-Israel, S.; Ensrud, K.E.; Song, Y.; Stone, K.L. Associations between sleep-disordered breathing, nocturnal hypoxemia, and subsequent cognitive decline in older community-dwelling men: The Osteoporotic Fractures in Men Sleep Study. J. Am. Geriatr. Soc. 2015, 63, 453–461. doi:10.1111/jgs.13321
6. Guglielmi, O.; Gómez, A.I.S.; Jurado-Gámez, B.; Buela-Casal, G. Obstructive sleep apnea syndrome effects on quality of life and daytime sleepiness. Univ. Psychol. 2013, 12, 601–611. doi:10.11144/Javeriana.UPSY12-2.esah
7. Tregear, S.; Reston, J.; Schoelles, K.; Phillips, B. Obstructive sleep apnea and risk of motor vehicle crash: Systematic review and meta-analysis. J. Clin. Sleep Med. 2009, 5, 573–581.
8. Jurado-Gámez, B.; Guglielmi, O.; Gude, F.; Buela-Casal, G. Workplace accidents, absenteeism and productivity in patients with sleep apnea. Arch. Bronconeumol. 2015, 51, 213–218. doi:10.1016/j.arbr.2014.12.002

9. Senaratna, C.V.; Perret, J.L.; Lodge, C.J.; Lowe, A.J.; Campbell, B.E.; Matheson, M.C.; Hamilton, G.S.; Dharmage, S.C. Prevalence of obstructive sleep apnea in the general population: a systematic review. Sleep Med. Rev. 2017, 34, 70–81. doi:10.1016/j.smrv.2016.07.002
10. Senaratna, C.V.; English, D.R.; Currier, D.; Perret, J.L.; Lowe, A.; Lodge, C.; Russell, M.; Sahabandu, S.; Matheson, M.C.; Hamilton, G.S.; Dharmage, S.C. Sleep apnea in Australian men: Disease burden, co-morbidities, and correlates from the Australian longitudinal study on male health. BMC Public Health 2016, 16, 51–61. doi:10.1186/s12889-016-3703-8
11. Young, T.; Peppard, P.E.; Taheri, S. Excess weight and sleep-disordered breathing. J. Appl. Physiol. 2005, 99, 1592–1599. doi:10.1152/japplphysiol.00587.2005
12. Ong, C.W.; O'Driscoll, D.M.; Truby, H.; Naughton, M.T.; Hamilton, G.S. The reciprocal interaction between obesity and obstructive sleep apnea. Sleep Med. Rev. 2013, 17, 123–131. doi:10.1016/j.smrv.2012.05.002
13. World Health Organization. Obesity and overweight. Available on: https://www.who.int/news-room/fact-sheets/detail/obesity-and-overweight
14. Epstein, L.J.; Kristo, D.; Strollo, P.J.; Friedman, N.; Malhotra, A.; Patil, S.P.; Ramar, K.; Rogers, R.; Schwab, R.J.; Weaver, E.M.; Weinstein, M.D. Clinical guideline for the evaluation, management and long-term care of obstructive sleep apnea in adults. J. Clin. Sleep Med. 2009, 5, 263–276.
15. Zhao, Y.Y.; Wang, R.; Gleason, K.J.; Lewis, E.F.; Quan, S.F.; Toth, C.M.; Morrical, M.; Rueschman, M.; Weng, J.; Ware, J.H.; Mittleman, M.A.; Redline, S. Effect of continuous positive airway pressure treatment on health-related quality of life and sleepiness in high cardiovascular risk individuals with sleep apnea: Best apnea interventions for research (BestAIR) trial. Sleep 2017, 40, zsx040. doi:10.1093/sleep/zsx040
16. Sánchez, A.I.; Martínez, P.; Miró, E.; Bardwell, W.A.; Buela-Casal, G. CPAP and behavioral therapies in patients with obstructive sleep apnea: effects on daytime sleepiness, mood, and cognitive function. Sleep Med. Rev. 2009, 13, 223–233. doi:10.1016/j.smrv.2008.07.002
17. Jurado-Gámez, B.; Guglielmi, O.; Gude-Sampedro, F.; Buela-Casal, G. Effect of CPAP therapy on job productivity and psychosocial occupational health in patients with moderate to severe sleep apnea. Sleep Breath. 2015, 19, 1293–1299. doi:10.1007/s11325-015-1162-6
18. Wolkove, N.; Baltzan, M.; Kamel, H.; Dabrusin, R.; Palayew, M. Long-term compliance with continuous positive airway pressure in patients with obstructive sleep apnea. Can. Respir. J. 2008, 15, 365–369. doi:10.1155/2008/534372
19. Morgenthaler, T.I.; Kapen, S.; Lee-Chiong, T.; Alessi, C.; Boehlecke, B.; Brown, T.; Coleman, J.; Friedman, L.; Kapur, V.; Owens, J.; Pancer, J.; Swick, T. Practice parameters for the medical therapy of obstructive sleep apnea. Sleep 2006, 29, 1031–1035.
20. Kolla, B.P.; Foroughi, M.; Saeidifard, F.; Chakravorty, S.; Wang, Z.; Mansukhani, M.P. The impact of alcohol on breathing parameters during sleep: a systematic

review and meta-analysis. Sleep Med. Rev. 2018, 42, 59–67. doi:10.1016/j.smrv.2018.05.007
21. Krishnan, V.; Dixon-Williams, S.; Thornton, J.D. Where there is smoke… there is sleep apnea: Exploring the relationship between smoking and sleep apnea. Chest 2014, 146, 1673–1680. doi:10.1378/chest.14-0772.
22. American Academy of Sleep Medicine. International Classifcation of Sleep Disorders, 3rd ed; American Academy of Sleep Medicine: Darien, IL, USA, 2014
23. Basoglu, O.K.; Tasbakan, M.S. Gender differences in clinical and polysomnographic features of obstructive sleep apnea: A clinical study of 2827 patients. Sleep Breath. 2018, 22, 241–249. doi:10.1007/s11325-017-1482-9
24. Harreiter, J.; Kautzky-Willer, A. Sex and gender differences in prevention of type 2 diabetes. Front. Endocrinol. 2018, 9, 220. doi:10.3389/fendo.2018.00220
25. Schulz, K.F.; Grimes, D.A. Generation of allocation sequences in randomised trials: chance, not choice. Lancet 2002, 359, 515–519. doi:10.1016/S0140-6736(02)07683-3
26. Friedberg, J.P.; Lipsitz, S.R.; Natarajan, S. Challenges and recommendations for blinding in behavioral interventions illustrated using a case study of a behavioral intervention to lower blood pressure. Patient Educ. Couns. 2010, 78, 5–11. doi:10.1016/j.pec.2009.04.009
27. Kapur, V.K.; Auckley, D.H.; Chowdhuri, S.; Kuhlmann, D.C.; Mehra, R.; Ramar, K.; Harrod, C.G. Clinical Practice Guideline for Diagnostic Testing for Adult Obstructive Sleep Apnea: An American Academy of Sleep Medicine Clinical Practice Guideline. J. Clin. Sleep Med. 2017, 13, 479–504. doi:10.5664/jcsm.6506.
28. Boudewyns, A.; Sforza, E.; Zamagni, M.; Krieger, J. Respiratory effort during sleep apneas after interruption of long-term CPAP treatment in patients with obstructive sleep apnea. Chest 1996, 110, 120–127. doi:10.1378/chest.110.1.120
29. Berry, R.B.; Budhiraja, R.; Gottlieb, D.J.; Gozal, D.; Iber, C.; Kapur, V.K.; Marcus, C.L.; Mehra, R.; Parthasarathy, S.; Quan, S.F.; Redline, S.; Strohl, K.P.; Ward, S.L.D.; Tangredi, M.M. Rules for scoring respiratory events in sleep: update of the 2007 AASM manual for the scoring of sleep and associated events. Deliberations of the sleep apnea definitions task force of the American Academy of Sleep Medicine. J. Clin. Sleep Med. 2012, 8, 597–619. doi:10.5664/jcsm.217
30. Jasper, H.H. Report of the committee on methods of clinical examination in electroencephalography: 1957. Electroencephalogr. Clin. Neurophysiol. 1958, 10, 370–375. doi:10.1016/0013-4694(58)90053-1
31. Rechtschaffen, A.; Kales, A. A manual of standardized terminology, techniques and scoring system of sleep stages in human subjects; Brain Information Service, Brain Research Institute, University of California: Los Angeles, CA, USA, 1968.
32. Mokhlesi, B.; Varga, A.W. Obstructive sleep apnea and cardiovascular disease: REM sleep matters! Am. J. Respir. Crit. Care Med. 2018, 197, 554–556. doi:10.1164/rccm.201710-2147ED.
33. Oja, P.; Laukkanen, R.; Pasanen, M.; Tyry, T.; Vuori, I. A 2-km walking test for assessing the cardiorespiratory fitness of healthy adults. Int. J. Sports Med. 1991, 12, 356–362. doi:10.1055/s-2007-1024694

34. Oja, P.; Tuxworth, B. Eurofit for adults: Assessment of health-related fitness. Council of Europe Publishing: Finland, 1995, pp. 1–13.
35. Ortega, F.B.; Sánchez-López, M.; Solera-Martínez, M.; Fernández-Sánchez, A.; Sjöström, M.; Martínez-Vizcaino, V. Self-reported and measured cardiorespiratory fitness similarly predict cardiovascular disease risk in young adults. Scand. J. Med. Sci. Sports 2013, 23, 749–757. doi:10.1111/j.1600-0838.2012.01454.x
36. Marfell-Jones, M.J.; Stewart, A.D.; de Ridder, J.H. International standards for anthropometric assessment; International Society for the Advancement of Kinanthropometry: Wellington, New Zealand, 2012.
37. Schröder, H.; Fitó, M.; Estruch, R.; Martínez-González, M.A.; Corella, D.; Salas-Salvadó, J.; Lamuela-Raventós, R.; Ros E.; Salaverría, I.; Fiol, M.; Lapetra, J.; Vinyoles, E.; Gómez-Gracia, E.; Lahoz, C.; Serra-Majem, L.; Pintó, X.; Ruiz-Gutierrez, V.; Covas, M.I. A short screener is valid for assessing Mediterranean diet adherence among older Spanish men and women. J. Nutr. 2011, 141, 1140–1145. doi:10.3945/jn.110.135566
38. Banna, J.C.; Townsend, M.S. Assessing factorial and convergent validity and reliability of a food behavior checklist for Spanish-speaking participants in US Department of Agriculture nutrition education programmes. Public Health Nutr. 2011, 14, 1165–1176. doi:10.1017/S1368980010003058
39. Becoña, E.; Gómez-Durán, B.; Alvarez-Soto, E.; García, M.P. [Scores of Spanish smokers on Fagerström's Tolerance Questionnaire]. Psychol. Rep. 1992, 71, 1227–1233.
40. Ferrer, M.; Vilagut, G.; Monasterio, C.; Montserrat, J.M.; Mayos, M.; Alonso, J. [Measurement of the perceived impact of sleep problems: the Spanish version of the functional outcomes sleep questionnaire and the Epworth sleepiness scale]. Med. Clin. (Barc). 1999, 113, 250–255.
41. Sanz, J.; Izquierdo, A.; García-Vera, M.P.; Dpto. I+D Pearson Clinical & Talent Assessment. BDI-FS, Inventario de Depresión de Beck para pacientes médicos; Pearson: Madrid, España, 2011.
42. Buela-Casal, G.; Guillén-Riquelme, A.; Seisdedos-Cubero, N. Cuestionario de Ansiedad Estado-Rasgo: Adaptación española, 9th ed.; TEA Ediciones: Madrid, España, 2016.
43. Spielberger, C.D.; Agudelo, D.; Buela-Casal, G. Inventario de Depresión Estado/Rasgo (IDER); TEA Ediciones: Madrid, España, 2008.
44. Rezaeitalab, F.; Moharrari, F.; Saberi, S.; Asadpour, H.; Rezaeetalab, F. The correlation of anxiety and depression with obstructive sleep apnea syndrome. J. Res. Med. Sci. 2014, 19, 205–210.
45. Chiner, E.; Arriero, J.M.; Signes-Costa, J.; Marco, J.; Fuentes, I. [Validation of the Spanish version of the Epworth Sleepiness Scale in patients with a sleep apnea syndrome]. Arch. Bronconeumol. 1999, 35, 422–427.
46. Becker, N.B.; de Jesus, S.N.; Viseu, J.N.; Stobäus, C.D.; Guerreiro, M.; Domingues, R.B. Depression and quality of life in older adults: Mediation effect of sleep quality. Int. J. Clin. Health Psychol. 2018, 18, 8–17. doi:10.1016/j.ijchp.2017.10.002

47. Wersebe, H.; Lieb, R.; Meyer, A.H.; Miche, M.; Mikoteit, T.; Imboden, C.; Hoyer, J.; Bader, K.; Hatzinger, M.; Gloster, A.T. Well-being in major depression and social phobia with and without comorbidity. Int. J. Clin. Health Psychol. 2018, 18, 201–208. doi:10.1016/j.ijchp.2018.06.004
48. Royuela, A.; Macías, J.A. Propiedades clinimétricas de la versión castellana del Cuestionario de Pittsburgh. Vigilia-Sueño 1997, 9, 81–94.
49. Catalán, P.; Martínez, A.; Herrejón, A.; Martínez-García, M.Á.; Soler-Cataluña, J.J.; Román-Sánchez, P.; Pinel, J.; Blanquer, R. [Internal consistency and validity of the Spanish version of the quality of life questionnaire specific for obstructive sleep apnea: Sleep apnea quality of life index]. Arch. Bronconeumol. 2012, 48, 431–442. doi:10.1016/j.arbres.2012.05.004
50. Alonso, J.; Prieto, L.; Antó, J.M. [The Spanish version of the SF-36 Health Survey (the SF-36 health questionnaire): an instrument for measuring clinical results]. Med. Clin. (Barc). 1995, 104, 771–776.
51. Lobo, A.; Pérez-Echeverría, M.J.; Artal, J. Validity of the scaled version of the General Health Questionnaire (GHQ-28) in a Spanish population. Psychol. Med. 1986, 16, 135–140.
52. Adan, A.; Almirall, H. Horne and Östberg morningness–eveningness questionnaire: A reduced scale. Pers. Indiv. Differ. 1991, 12, 241–253. doi:10.1016/0191-8869(91)90110-W
53. Kim, L.J.; Coelho, F.M.; Hirotsu, C.; Bittencourt, L.; Tufik, S.; Andersen, M.L. Is the chronotype associated with obstructive sleep apnea? Sleep Breath. 2015, 19, 645–651. doi:10.1007/s11325-014-1070-1
54. Lucassen, E.A.; Zhao, X.; Rother, K.I.; Mattingly, M.S.; Courville, A.B.; de Jonge, L.; Csako, G.; Cizza, G. Evening chronotype is associated with changes in eating behavior, more sleep apnea, and increased stress hormones in short sleeping obese individuals. PLoS One 2013, 8, e56519. doi:10.1371/journal.pone.0056519
55. Prochaska, J.O.; Velicer, W.F. The transtheoretical model of health behavior change. Am. J. Health Promot. 1997, 12, 38–48. doi:10.4278/0890-1171-12.1.38
56. Burgess, E.; Hassmén, P.; Welvaert, M.; Pumpa, K.L. Behavioral treatment strategies to improve adherence to lifestyle intervention programmes in adults with obesity: A systematic review and meta-analysis. Clin. Obes. 2017, 7, 105–114. doi:10.1111/cob.12180
57. Guillermo, C.; Boushey, C.J.; Franke, A.A.; Monroe, K.R.; Lim, U.; Wilkens, L.R.; Le Marchand, L.; Maskarinec, G. Diet quality and biomarker profiles related to chronic disease prevention: The Multiethnic Cohort Study. J. Am. Coll. Nutr. 2019, 10, 1–8. doi:10.1080/07315724.2019.1635921
58. St-Onge, M.P.; Mikic, A.; Pietrolungo, C.E. Effects of diet on sleep quality. Adv. Nutr. 2016, 7, 938–949. doi:10.3945/an.116.012336
59. Bozkurt, N.C.; Cakal, E.; Sahin, M.; Ozkaya, E.C.; Firat, H.; Delibasi, T. The relation of serum 25-hydroxyvitamin-D levels with severity of obstructive sleep apnea and glucose metabolism abnormalities. Endocrine 2012, 41, 518–525. doi:10.1007/s12020-012-9595-1

60. Gao, Q.; Kou, T.; Zhuang, B.; Ren, Y.; Dong, X.; Wang, Q. The Association between vitamin D deficiency and sleep disorders: A systematic review and meta-analysis. Nutrients 2018, 10, 1395. doi:10.3390/nu10101395
61. Cao, Y.; Zhen, S.; Taylor, A.W.; Appleton, S.; Atlantis, E.; Shi, Z. Magnesium intake and sleep disorder symptoms: Findings from the Jiangsu Nutrition Study of Chinese adults at five-year follow-up. Nutrients 2018, 10, 1354. doi:10.3390/nu10101354
62. McHill, A.W.; Wright, K.P.Jr. Role of sleep and circadian disruption on energy expenditure and in metabolic predisposition to human obesity and metabolic disease. Obes. Rev. 2017, 18, 15–24. doi:10.1111/obr.12503
63. Yang, C.L.; Schnepp, J.; Tucker, R.M. Increased hunger, food cravings, food reward, and portion size selection after sleep curtailment in women without obesity. Nutrients 2019, 11, 663. doi:10.3390/nu11030663
64. St-Onge, M.P.; Wolfe, S.; Sy, M.; Shechter, A.; Hirsch, J. Sleep restriction increases the neuronal response to unhealthy food in normal-weight individuals. Int. J. Obes. 2014, 38, 411–416. doi:10.1038/ijo.2013.114
65. St-Onge, M.P.; McReynolds, A.; Trivedi, Z.B.; Roberts, A.L.; Sy, M.; Hirsch, J. Sleep restriction leads to increased activation of brain regions sensitive to food stimuli. Am. J. Clin. Nutr. 2012, 95, 818–824. doi:10.3945/ajcn.111.027383
66. Wallace, A.; Bucks, R.S. Memory and obstructive sleep apnea: A meta-analysis. Sleep 2013, 36, 203–220. doi:10.5665/sleep.2374
67. Martin, A.A.; Davidson, T.L.; McCrory, M.A. Deficits in episodic memory are related to uncontrolled eating in a sample of healthy adults. Appetite 2018, 124, 33–42. doi:10.1016/j.appet.2017.05.011
68. Aiello, K.D.; Caughey, W.G.; Nelluri, B2.; Sharma, A.; Mookadam, F.; Mookadam, M. Effect of exercise training on sleep apnea: A systematic review and meta-analysis. Respir. Med. 2016, 116, 85–92. doi:10.1016/j.rmed.2016.05.015
69. Iftikhar, I.H.; Kline, C.E.; Youngstedt, S.D. Effects of exercise training on sleep apnea: a meta-analysis. Lung 2014, 192, 175e84. doi:10.1007/s00408-013-9511-3
70. Shook, R.P.; Hand, G.A.; Drenowatz, C.; Hebert, J.R.; Paluch, A.E.; Blundell, J.E.; Hill, J.O.; Katzmarzyk, P.T.; Church, T.S.; Blair, S.N. Low levels of physical activity are associated with dysregulation of energy intake and fat mass gain over 1 year. Am. J. Clin. Nutr. 2015, 102, 1332–1338. doi:10.3945/ajcn.115.115360
71. Shechter, A. Obstructive sleep apnea and energy balance regulation: A systematic review. Sleep Med. Rev. 2017, 34, 59–69. doi:10.1016/j.smrv.2016.07.001
72. Ueno, L.M.; Drager, L.F.; Rodrigues, A.C.; Rondon, M.U.; Braga, A.M.; Mathias, W.Jr.; Krieger, E.M.; Barretto, A.C.; Middlekauff, H.R.; Lorenzi-Filho, G.; Negrão C.E.. Effects of exercise training in patients with chronic heart failure and sleep apnea. Sleep 2009, 32, 637–647. doi:10.1093/sleep/32.5.637
73. Redolfi, S.; Arnulf, I.; Pottier, M.; Bradley, T.D.; Similowski, T. Effects of venous compression of the legs on overnight rostral fluid shift and obstructive sleep apnea. Respir. Physiol. Neurobiol. 2011, 175, 390–393. doi:10.1016/j.resp.2011.01.001
74. Evans, R.A.; Dolmage, T.E.; Robles, P.G.; Brooks, D.; Goldstein, R.S. The effects of exercise modality and intensity on energy expenditure and cardiorespiratory

response in adults with obesity and treated obstructive sleep apnea. Chron. Respir. Dis. 2017, 14, 342–351. doi:10.1177/1479972316643699
75. Buela-Casal, G.; Sierra, J. C. Evaluación y tratamiento de los trastornos del sueño [Evaluation and treatment of sleep disorders]. In Manual de Evaluación y Tratamientos Psicológicos, 2nd ed.; Buela-Casal, G., Sierra, J.C., Eds.; Biblioteca Nueva: Madrid, España, 2004; pp. 393–438.
76. Kitamura, T.; Miyazaki, S.; Koizumi, H.; Takeuchi, S.; Tabata, T.; Suzuki, H. Sleep hygiene education for patients with obstructive sleep apnea. Sleep Biol. Rhythms 2016, 14, 101–106. doi:10.1007/s41105-015-0015-9
77. Chakravorty, I.; Cayton, R.M.; Szczepura, A. Health utilities in evaluating intervention in the sleep apnea/hypopnea syndrome. Eur. Respir. J. 2002, 20, 1233–1238.
78. Ackel-D'Elia, C.; da Silva, A.C.; Silva, R.S.; Truksinas, E.; Sousa, B.S.; Tufik, S.; de Mello, M.T.; Bittencourt, L.R. Effects of exercise training associated with continuous positive airway pressure treatment in patients with obstructive sleep apnea syndrome. Sleep Breath. 2012, 16, 723–735. doi:10.1007/s11325-011-0567-0
79. Schrand, J.R. Is sleep apnea a predisposing factor for tobacco use? Med. Hypotheses 1996, 47, 443 - 448.
80. Becoña, E. Programa para dejar de fumar [Program for smoking cessation]; Nova Galicia Edicións: Vigo, España, 2007.
81. Foxx, R. M., & Brown, R. A. (1979). Nicotine fading, self-monitoring for cigarette abstinence or controlled smoking. J. Applied Behav. Anal. 1979, 12, 115–125.
82. Becoña, E.; García, M.P. Nicotine fading and smokeholding methods to smoking cessation. Psychol. Rep. 1993, 73, 779–786.
83. Simou, E.; Britton, J.; Leonardi-Bee, J. Alcohol and the risk of sleep apnea: a systematic review and meta-analysis. Sleep Med. 2018, 42, 38–46. doi:10.1016/j.sleep.2017.12.005
84. Issa, F.G.; Sullivan, C.E. Alcohol, snoring and sleep apnea. J. Neurol. Neurosurg. Psychiatry 1982, 45, 353–359.
85. Borrelli, B. The assessment, monitoring, and enhancement of treatment fidelity in public health clinical trials. J. Public Health Dent. 2011, 71, 52–63. doi:10.1111/j.1752-7325.2011.00233.x
86. Amaro-Gahete, F.J.; De-la-O, A.; Jurado-Fasoli, L.; Espuch-Oliver, A.; Robles-González, L.; Navarro-Lomas, G.; de Haro, T.; Femia, P.; Castillo, M.J.; Gutierrez, A. Exercise training as S-Klotho protein stimulator in sedentary healthy adults: Rationale, design, and methodology. Contemp. Clin. Trials Commun. 2018, 11, 10–19. doi:10.1016/j.conctc.2018.05.013
87. Sánchez-Delgado, G.; Martinez-Tellez, B.; Olza, J.; Aguilera, C.M.; Labayen, I.; Ortega, F.B.; Chillon, P.; Fernandez-Reguera, C.; Alcantara, J.M.A.; Martinez-Avila, W.D.; Muñoz-Hernandez, V.; Acosta, F.M.; Prados-Ruiz, J.; Amaro-Gahete, F.J.; Hidalgo-Garcia, L.; Rodriguez, L..; Ruiz, Y.A..; Ramirez-Navarro, A.; Muros-de Fuentes, M.A.; García-Rivero, Y.; Sanchez-Sanchez, R.; de Dios Beas Jimenez, J.; de Teresa, C.; Navarrete, S.; Lozano, R.; Brea-Gomez, E.; Rubio-Lopez, J.; Ruiz, M.R.; Cano-Nieto, A.; Llamas-Elvira, J.M.; Jimenez Rios, J.A.; Gil, A.; Ruiz, J.R.

Activating brown adipose tissue through exercise (ACTIBATE) in young adults: Rationale, design and methodology. Contemp. Clin. Trials 2015, 45, 416–425. doi:10.1016/j.cct.2015.11.004
88. Bates, D.; Maechler, M.; Bolker, B.M.; Walker, S.C. Fitting linear mixed-effects models using lme4. J. Stat. Softw. 2015, 67, 1-48. doi:10.18637/jss.v067.i01
89. Berger, V.W. The reverse propensity score to detect selection bias and correct for baseline imbalances. Stat Med. 2005, 24, 2777–2787. doi:10.1002/sim.2141

Summary of Changes to Study Protocol

Date	Amendment
22/02/2019	Registration of the trial in ClinicalTrials.gov and attainment of the Institutional Ethics Committee approvals (sections 3.1. and 5.1.).
7/06/2019	Addition of a two-dimensional lumbar spine dual-energy X-ray absorptiometry (DXA) scanner in order to obtain the trabecular bone score (TBS) for a non-compulsory sub-study (section 3.5.).
6/12/2019	Removal of the Psychomotor Vigilance Test from all trial assessments (sections 3.4. and 3.5.).
23/10/2020	Revision and editing of the statistical analysis plan including the change of the R statistical software package to be used for estimation of intervention effects on primary and secondary outcomes based on linear mixed-effects models (section 3.10).

Original Statistical Analysis Plan (Sections 3.10 and 3.11 from Study Protocol Version 1.0)

Power and Sample Size. The sample size and power of the INTERAPNEA trial will be estimated based on previous studies synthesized in our recent systematic review and meta-analysis [1]. Assuming a standard deviation of 11.98 in our primary endpoint (AHI pooled standard deviation of previous research), we estimate that the enrollment of 35 participants per arm of our trial will provide a statistical power of 90% at an alpha level of 0.05 to detect a minimum effect size of -8.36 in AHI (pooled mean difference of previous trials) [1]. However, considering a maximum dropout rate of 17.25% (average dropout rate of previous studies [1]), we will recruit 42 participants for each trial group. Owing to practical and feasibility reasons, the trial will be conducted in three consecutive sets of a maximum of 30 participants.

Data Analyses. Descriptive and exploratory preliminary analyses of all the study variables will be performed to reveal violations of statistical assumptions, distributions, imbalances between the study groups, associations between study variables, covariates/confounders, amount of missing data, and drop-out patterns.

The intervention effects on primary and secondary study outcomes will be assessed through multi-level mixed analyses using the package *nlme* [2] from the R statistical program. This method will allow us to analyze differences including time and group allocation as within and between subject factors, respectively, adjusting for potential covariates or levels such as set of participants. Therefore, time and group/condition will be considered as fixed variables, and sets/waves of participants as a random factor. With these specifications, we will be able to control the intra-class correlation (per set of participants nested within conditions) between scores at baseline and post-test or follow-up. Attrition propensity will be calculated using a model predicting the actual attrition with baseline values [3]. Baseline measurements included in these attrition propensity prediction models will include set of participants, participant's allocation, age, OSA severity, BMI, and motivation to change. All analyses will be performed with an intention-to-treat (ITT) approach, including all participants as originally allocated after randomization. Per-protocol comparison of groups including only those participants who fully completed the originally allocated treatment will also be computed and compared with ITT analysis results.

References

1. Carneiro-Barrera, A.; Díaz-Román, A.; Guillén-Riquelme, A.; Buela-Casal, G. Weight loss and lifestyle interventions for obstructive sleep apnea in adults: Systematic review and meta-analysis. (Unpublished)
2. Pinheiro, J.; Bates, D.; DebRoy, S.; Sarkar, D.; R Core Team. nlme: Linear and nonlinear mixed effects models. R package version 3.1-137. Available on: https://CRAN.R-project.org/package=nlme
3. Berger, V.W. The reverse propensity score to detect selection bias and correct for baseline imbalances. Stat Med. 2005, 24, 2777–2787. doi:10.1002/sim.2141

Final Statistical Analysis Plan (Sections 3.10 and 3.11 from Study Protocol Version 2.0)

Power and Sample Size. The sample size and power of the INTERAPNEA trial will be estimated based on previous studies synthesized in our recent systematic review and meta-analysis [1]. Assuming a standard deviation of 11.98 in our primary endpoint (AHI pooled standard deviation of previous research), we estimate that the enrollment of 35 participants per arm of our trial will provide a statistical power of 90% at an alpha level of 0.05 to detect a minimum effect size of -8.36 in AHI (pooled mean difference of previous trials) [1]. However, considering a maximum dropout rate of 17.25% (average dropout rate of previous studies [1]), we will recruit 42 participants for each trial group. Owing to practical and feasibility reasons, the trial will be conducted in three consecutive sets of a maximum of 30 participants.

Data Analyses. We will perform descriptive and exploratory preliminary analyses of all the study variables to reveal violations of statistical assumptions, distributions, imbalances between the study groups, associations between study variables, covariates/confounders, amount of missing data, and drop-out patterns.

Intervention effects on primary and secondary outcomes will be assessed based on linear mixed-effects models using the package *lme4* [2] from the R statistical program, with individual measures of growth being modeled as the function of randomly assigned group, assessment time, and the interaction between group and time (fixed variables). If required, effects of set of participants will also be included in the model as a random cluster factor. Estimations will be performed using the restricted maximum-likelihood method, including an unstructured covariance matrix to adjust for within-participant clustering resulting from the repeated-measures design. The model will assume that missing values are missing-at-random. Nevertheless, attrition propensity will be calculated using a logistic model predicting attrition with baseline values of set of participants, allocation group, OSA severity, age and BMI [3].

All estimations and analyses will be performed with an intention-to-treat approach (including all participants as originally allocated after randomization) and an additional per-protocol approach restricted to participants with a CPAP usage greater or equal to 4 hours per night on 70% of nights and, regarding the intervention group, at least 80% of attendance rate at intervention sessions.

References

1. Carneiro-Barrera, A.; Díaz-Román, A.; Guillén-Riquelme, A.; Buela-Casal, G. Weight loss and lifestyle interventions for obstructive sleep apnea in adults: Systematic review and meta-analysis. Obes. Rev. 2019, 20, 750–762. doi:10.1111/obr.12824
2. Bates, D.; Maechler, M.; Bolker, B.M.; Walker, S.C. Fitting linear mixed-effects models using lme4. J. Stat. Softw. 2015, 67, 1-48. doi:10.18637/jss.v067.i01
3. Berger, V.W. The reverse propensity score to detect selection bias and correct for baseline imbalances. Stat Med. 2005, 24, 2777–2787. doi:10.1002/sim.2141

Summary of Changes to Statistical Analysis Plan

There have been no major changes to the statistical analysis plan. The plan outlined in the Study Protocol Version 1.0 was minimally revised and edited in the Study Protocol Version 2.0, including the adjustment of the R statistical software package to be used for the primary analyses. Estimations of the intervention effects on primary and secondary outcomes were based on linear mixed-effects models, with individual measures of growth being modeled as the function of randomly assigned group, assessment time, and the interaction between group and time. Owing to no significant variation across set of participants, the model was only adjusted for the within-participant clustering resulting from the repeated-measures design. All estimations and analyses were performed with an intention-to-treat approach and an additional per-protocol approach.
