# Supplementary material for: Effect of an Interdisciplinary Weight Loss and Lifestyle Intervention on Obstructive Sleep Apnea Severity: The INTERAPNEA Randomized Clinical Trial
Source: JAMA Netw Open. 2022 Apr 22;5(4):e228212. doi: 10.1001/jamanetworkopen.2022.8212 (PMC9034401; doi:10.1001/jamanetworkopen.2022.8212)
Supplement: Supplement 2. — eMethods 1. Study Organization and Eligibility Criteria eMethods 2. Study Assessments and End Points eMethods 3. Weight Loss and Lifestyle Intervention eMethods 4. Assessment of Adherence and Integrity of Intervention and Intervention Adherence eTable 1. Missing Values in Secondary Cardiometabolic Risk End Points eTable 2. Baseline Characteristics of Study Participants (Per-Protocol Approach) eTable 3. Primary and Secondary Sleep-Related End Points (Per-Protocol Approach) eTable 4. Primary and Secondary Sleep-Related End Points (Change From 8 Weeks to 6 Months) eTable 5. Secondary Body Composition and Cardiometabolic Risk End Points (Per-Protocol Approach) eTable 6. Secondary Body Composition and Cardiometabolic Risk End Points (Change From 8 Weeks to 6 Months) eTable 7. Health-Related Quality of Life End Points eTable 8. Health-Related Quality of Life End Points (Change From 8 Weeks to 6 Months) eTable 9. Physical Activity and Dietary Behavior End Points eTable 10. Physical Activity and Dietary Behavior End Points (Change From 8 Weeks to 6 Months) eFigure 1. Obstructive Sleep Apnea Severity at Baseline and 8 Weeks After Intervention in the Intervention Group eFigure 2. Apnea-Hypopnea Index End Point (Change From 8 Weeks to 6 Months) eFigure 3. Association Between Changes in Apnea-Hypopnea Index Over Time and Changes in Body Mass Index eReferences [file jamanetwopen-e228212-s002.pdf]

Supplementary Online Content

Carneiro-Barrera A, Amaro-Gahete FJ, Guillén-Riquelme A, et al. Effect of an interdisciplinary weight loss and lifestyle intervention on obstructive sleep apnea severity: the INTERAPNEA randomized clinical trial. *JAMA Netw Open*. 2022;5(4):e228212. doi:10.1001/jamanetworkopen.2022.8212

eMethods 1. Study Organization and Eligibility Criteria

eMethods 2. Study Assessments and End Points

eMethods 3. Weight Loss and Lifestyle Intervention

eMethods 4. Assessment of Adherence and Integrity of Intervention and Intervention Adherence

eTable 1. Missing Values in Secondary Cardiometabolic Risk End Points

eTable 2. Baseline Characteristics of Study Participants (Per-Protocol Approach)

eTable 3. Primary and Secondary Sleep-Related End Points (Per-Protocol Approach)

eTable 4. Primary and Secondary Sleep-Related End Points (Change From 8 Weeks to 6 Months)

eTable 5. Secondary Body Composition and Cardiometabolic Risk End Points (Per-Protocol Approach)

eTable 6. Secondary Body Composition and Cardiometabolic Risk End Points (Change From 8 Weeks to 6 Months)

eTable 7. Health-Related Quality of Life End Points

eTable 8. Health-Related Quality of Life End Points (Change From 8 Weeks to 6 Months)

eTable 9. Physical Activity and Dietary Behavior End Points

eTable 10. Physical Activity and Dietary Behavior End Points (Change From 8 Weeks to 6 Months)

eFigure 1. Obstructive Sleep Apnea Severity at Baseline and 8 Weeks After Intervention in the Intervention Group

eFigure 2. Apnea-Hypopnea Index End Point (Change From 8 Weeks to 6 Months)

eFigure 3. Association Between Changes in Apnea-Hypopnea Index Over Time and Changes in Body Mass Index

eReferences

This supplementary material has been provided by the authors to give readers additional information about their work.

© 2022 Carneiro-Barrera A et al. *JAMA Network Open*.

eMethods 1. Study Organization and Eligibility Criteria

Study Organization

The Sleep and Health Promotion Laboratory of the Mind, Brain and Behavior Research Center (CIMCYC) from the University of Granada (Granada, Spain) was responsible for the study design and organization, participant recruitment process, data collection and management, randomization and participant allocation, trial monitoring, and reporting of the study process and results. Participants previously diagnosed with moderate-to-severe OSA and potentially meeting the inclusion criteria were recruited from the collaborating sleep-disordered breathing unit of the Virgen de las Nieves University Hospital (Granada, Spain). Data collection at baseline and intervention endpoint, as well as implementation of the weight loss and lifestyle intervention, was performed in two different settings of the University of Granada (Granada, Spain): the Sleep and Health Promotion Laboratory (CIMCYC) and the Sport and Health University Research Institute (iMUDS). General and specific OSA standard care (i.e., continuous positive airway pressure [CPAP]) of all participants enrolled in the trial continued being provided by their primary care team from the Virgen de las Nieves University Hospital.

Eligibility Criteria

The INTERAPNEA eligibility criteria was based on a thorough consideration of the potential generalizability of results and its external validity. A common and substantial limitation of randomized controlled trials (RCTs; mostly efficacy/explanatory trials) is the use of stringent eligibility criteria to minimize adverse events and potential nonresponders; which results in a highly selected sample unrepresentative of the overall population.[1] To ensure external validity, trials should include a sample with those comorbidities and use of medication, as well as variable compliance rates, that are commonly found in the general population affected by the condition under consideration.[1-3]

Therefore, eligible participants of the INTERAPNEA trial were men aged between 18 and 65 years with CPAP-treated moderate-to-severe OSA (apnea-hypopnea index [AHI] equal or greater than 15 events/hour), and a body mass index (BMI) equal or greater than 25 kg/m^2. A CPAP use for at least 4 hours a day for 5 or more days per week reported by the participant was considered CPAP compliance. The sole inclusion of men in our sample was not only based on the higher incidence and prevalence of OSA in this population,[4] but also

© 2022 Carneiro-Barrera A et al. *JAMA Network Open*.

on the well-evidenced differences between men and women in OSA phenotypes[5] and the effectiveness of weight loss interventions.[6-9]

Regarding exclusion criteria, this only included current participation in a weight loss program, presence of any psychological/psychiatric disorder including alcohol or other substance dependence or abuse, coexistence of any other primary sleep disorder which was not secondary to OSA, and daily use/prescribed sedative-hypnotic medication. To ensure no hindrance/harm related to the assessment and intervention protocols, all participants were also medically examined and completed a health history revision prior to their final enrollment in the trial. Nonetheless, clinical trial liability insurance was also contracted for the provision of legal and financial protection of the sponsors-investigators and participants of the trial.

© 2022 Carneiro-Barrera A et al. *JAMA Network Open*.

eMethods 2. Study Assessments and End Points

Assessments of primary and secondary endpoints of the trial were organized and completed at baseline, 8 weeks (intervention endpoint) and 6 months after intervention over three different days during a one to two-week period:[10]

• Day 1: Potential participants attended a medical examination and blood test after a 12-hour overnight fast at the Sleep Unit of the Virgen de las Nieves University Hospital.

• Day 2: Eligible participants completed a full-night in-laboratory polysomnography (PSG; the gold-standard objective sleep testing recommended by the American Academy of Sleep Medicine (AASM),[11] at the Sleep and Health Promotion Laboratory (CIMCYC). In order to avoid potential CPAP influence on PSG endpoints, participants were required to withdraw from CPAP during the week prior to the PSG at baseline and follow-ups.[12] Prior to the PSG, participants also completed a set of questionnaires assessing subjective endpoints related to sleep, general physical and psychological health, and lifestyle habits including diet, physical exercise, and alcohol and tobacco consumption.

• Day 3: Participants were required to attend the Sport and Health University Research Institute (iMUDS) for the measurement of anthropometric parameters and body composition through a full-body dual energy X-ray absorptiometry (DXA) scanner.

Baseline physical activity and sleep habits were also be obtained through a seven-day self-reported daily step/km log and sleep diary.

Primary Endpoint

The primary endpoint of the INTERAPNEA trial was the change in OSA severity as measured by AHI, defined as the number of apnea (90% or greater drop in airflow for 10 s or longer) and hypopneas (30% or greater drop in airflow for 10 s or longer associated with ≥3% oxygen desaturation or an arousal) episodes per hour of sleep.[13] This primary endpoint and other neurophysical and cardiorespiratory secondary endpoints were measured through an in-laboratory PSG using SOMNOScreen™ PSG-Tele (SOMNOmedics, GmbH, Randersacker, Germany), or Somté PSG v2 system (Compumedics Limited, Abbotsford, Australia). The recordings included all recommended physiologic signals, namely electroencephalogram (three channels: F4-M1, C4-M1, O2-M1), electrooculogram (two channels: E1 and E2), electromyogram (two channels: submental and anterior tibialis muscles), and electrocardiogram (two channels). Cardiorespiratory measurements included

oral and nasal airflow (triple thermistor), oxyhaemoglobin saturation (SpO_2) and pulse-rate (pulse oximeter), respiratory effort (chest and abdomen bands), and body position (sensor). All electrodes were placed in accordance with the international 10–20 system,[14] and recordings were automatically and manually scored in 30 s epochs[15] by trained physicians using DOMINO (v2.7, SOMNOmedics, GmbH, Randersacker, Germany), or ProFusion PSG 3 (v3.3, Compumedics Limited, Abbotsford, Australia) associated computer software. All parameters, settings, filters, technical specifications, sleep stage scoring and event scoring were performed in accordance with the AASM Manual for the Scoring of Sleep and Associated Events.[13] Owing to the more severe cardiovascular consequences of apnea episodes in rapid eye movement (REM) sleep as compared with non-REM obstructions,[16-18] we also specifically analyzed AHI in REM sleep and non-REM sleep stages (N1, N2, and N3); which has generally been disregarded in previous similar RCTs.[6]

Secondary Sleep-Related Endpoints

Secondary polysomnographic endpoints related to OSA and measured by PSG were changes in oxygen desaturation index (number of oxygen desaturation ≥3% per hour of sleep), SpO_2 mean (%; average of oxygen saturation during sleep), SpO_2 nadir (%; minimum oxygen saturation during sleep), percentage of time with SpO_2 <90% during sleep, sleep efficiency (%; total sleep time/total time in bed), sleep latency (min; time from lights out to first epoch of any sleep stage), wake after sleep onset (min; time awake in bed minus sleep latency), N1+N2 sleep stages (%; N1+N2 sleep stages/total sleep time), N3 sleep stage (%; N3 sleep stage/total sleep time), and REM sleep stage (%; REM sleep stage/total sleep time). Other secondary sleep-related endpoints included changes in subjective sleep quality and daytime sleepiness as measured by the Pittsburgh Sleep Quality Index (PSQI)[19] and the Epworth Sleepiness Scale (ESS),[20] respectively. The PSQI includes a total of 19 self-rated items combined to form seven component scores (i.e., subjective sleep quality, sleep latency, sleep duration, habitual sleep efficiency, sleep disturbances, use of sleeping medications, and daytime dysfunction), each of which has a range of 0–3 points. The sum of these seven PSQI component scores is considered to obtain a global PSQI score (ranging from 0–21), with higher scores indicating poorer sleep quality.[19] The ESS is a widely used 8 item Likert-based scale that measures propensity for dozing during several daily activities, its total score ranging from 0 to 24 (the sum of the 8 item scores, 0–3). Hypersomnolence is considered when a total score greater than 10 is attained.[20]

Secondary Body Composition Endpoints

Body anthropometric endpoints included changes in weight, BMI, and neck, chest and waist circumferences. Body weight and height were measured using a calibrated scale and stadiometer (model 799, Electronic Column Scale, Hamburg, Germany) with participants wearing undergarments. Neck, chest and waist circumferences were measured following standard procedures recommended by the International Society for the Advancement of Kinanthropometry (ISAK).[21] Body composition endpoints including changes in fat mass (kg), visceral adipose tissue (g), and lean mass (kg) were obtained through a full-body DXA scanner (Discovery Wi, Hologic, Inc., Bedford, MA, USA). Quality controls, positioning of participants and analyses of results were performed following the manufacturer's recommendations. Automatic delineation of anatomic regions was conducted using APEX 4.0.2. software.

Secondary Cardiometabolic Risk Endpoints

Cardiometabolic risk endpoints included changes in glucose metabolism (glucose [mg/dl], insulin [IU/ml] and insulin resistance as indicated by the homeostasis model assessment of insulin resistance [HOMA-IR] index), lipid metabolism (total cholesterol [mg/dl], high-density lipoprotein cholesterol [HDL-C; mg/dl], low-density lipoprotein cholesterol [LDL-C; mg/dl], triglycerides [mg/dl], and apolipoproteins A1 and B [mg/dl]), and liver function (aspartate aminotransferase [AST; IU/l], alanine aminotransferase [ALT; IU/l], γ-glutamyltransferase [γ-GT], and fatty liver index [FLI]). These endpoints were measured through blood samples obtained from participants' antecubital vein in a supine position during the morning in a fasting state. Samples were collected into prechilled ethylene diamine tetra-acetic acid-containing tubes (Vacutainer SST, Becton Dickinson, Plymouth, UK) and immediately centrifuged (i.e. 15 min at 3,000 rpm), aliquoted and stored at −80°C for further plasma analysis. Glucose levels were measured by spectrophotometric techniques (AU5800, Beckman Coulter, Brea, California, USA). Insulin was assessed by chemiluminescence immunoassay with paramagnetic particles (UniCel DxI 800, Beckman Coulter, Brea, California, USA). HOMA-IR was calculated as fasting glucose (mmol/l) times the level of fasting insulin (UU/ml) divided by 22.5.[22] Total cholesterol, HDL-C, triglycerides, and apolipoproteins A1 and B were automatically evaluated by spectrophotometric techniques (AU5800, Beckman Coulter, Brea, California, USA). LDL-C was calculated as the level of total cholesterol minus the level of HDL-C minus 0.45 times the level of triglycerides. AST, ALT and γ-GT were calculated by absorption spectrophotometric techniques (Beckman Coulter, Brea, California, USA). FLI was calculated with the formula FLI = ((e 0.953·loge (Triglycerides) + 0.139·BMI +

© 2022 Carneiro-Barrera A et al. *JAMA Network Open*.

$0.718 \cdot \log_e (\gamma\text{-GT}) + 0.053 \cdot \text{Waist Circumference} - 15.745)) / ((1 + e^{0.953 \cdot \log_e (\text{Triglycerides}) + 0.139 \cdot \text{BMI} + 0.718 \cdot \log_e (\gamma\text{-GT}) + 0.053 \cdot \text{Waist Circumference} - 15.745})) \cdot 100$.[23]

Changes in systolic blood pressure (mm Hg), diastolic blood pressure (mm Hg) and mean blood pressure (mm Hg) were also considered as cardiometabolic risk endpoints. Blood pressure was measured with an ambulatory blood pressure monitor (Omron M3 Blood Pressure Monitor, OMRON Healthcare, Hoofddorp, Netherlands) in a sitting position after at least five min of rest. The mean of two measurements was recorded. Mean blood pressure was calculated at each reading as one third of systolic pressure plus two thirds of diastolic pressure.

Additional Health-Related Quality of Life and Lifestyle Habits Endpoints

Additional endpoints included health-related quality of life and lifestyle habits. Health-related quality of life scores were obtained through the Sleep Apnea Quality of Life (SAQLI)[24] and the Medical Outcomes Study 36-Item Short-Form Health Survey (SF-36).[25,26] SAQLI is a 35 item self-report instrument assessing OSA specific quality of life through four core domains: daily functioning, social interactions, emotional functioning, and symptoms. The total score (average of the four components, 1 to 7) ranges from 1 to 7, with higher scores indicating better health-related quality of life.[24] SF-36 is also a widely used self-report measure of quality of life composed by eight scaled scores (vitality, physical functioning, bodily pain, general health perceptions, physical role functioning, emotional role functioning, social role functioning, and mental health). Final physical and mental component summaries scores range from 0 to 100, with higher scores indicating better health-related quality of life with respect to either the physical or mental component.[25,26]

The Food Behavior Checklist (FBC)[27] was used to assess participants' food intake and habits. FBC comprises seven subscales including consumption of fruit and vegetables (9 items), diet quality (4 items), fast food (3 items), dairy/calcium (2 items), sweetened beverages (2 items), meat (1 item) and food security (1 item). A total FBC score was calculated as the sum of these subscales ranging from 22 to 81, with higher scores indicating healthier dietary pattern.[27] Physical activity was measured using daily step/km logs recorded by participants with a spring-levered pedometer. Participants were required to wear the pedometer all day and register the number of step/km achieved per day in a seven-day step log. The average step/km per day was then calculated at baseline and follow-ups. Regarding the remaining lifestyle habits, smoking and alcohol intake were measured at baseline and follow-ups using seven-day self-reported tobacco and alcohol

consumption logs. Recordings included number of cigarettes/alcoholic units consumed per day, cigarette brand/type of alcoholic drink, time, situation, and perceived pleasure (from 0 to 10).

© 2022 Carneiro-Barrera A et al. *JAMA Network Open*.

eMethods 3. Weight Loss and Lifestyle Intervention

The design and implementation of the INTERAPNEA weight loss and lifestyle intervention was based on results of previous research[6,28] and the most recent international evidenced-based clinical practice guidelines for the management of obesity and OSA.[29-31] The intervention lasted eight weeks and was composed of five different modules: nutritional behavior change, moderate aerobic exercise, smoking reduction and cessation, alcohol intake avoidance, and sleep hygiene. Each component included group-based weekly sessions of 60-90 min lead and supervised by a trained professional in the field (i.e. human nutrition and dietetics, physical activity and sport sciences, clinical psychology, and sleep medicine).

The cornerstone of this intervention was the use of the Transtheoretical Model of Health Behavior Change,[32] a well-recognized biopsychosocial model based on integrating key strategies, processes and principles of behavior change theories into a comprehensive interventional approach for the achievement of sustainable health-related behaviors.[32] The general behavioral change techniques used in each component of the intervention included motivation and preparation for action; goal-setting and action-planning; self-monitoring and functional behavioral analysis; review of behavioral goals, action plans, and adherence; problem solving and social skills; and self-efficacy, maintenance, and relapse prevention (a detailed description and timing of the INTERAPNEA intervention modules and components, as well as the rationale and specific session topics of each module, has previously been published).[10]

Nutritional Behavior Change[10]

The nutrition module consisted of eight 60-90-min sessions in a group format addressing dietary patterns using integrated techniques of nutrition education and behavioral change such as goal-setting, cognitive restructuring, stimulus control, progressive muscle relaxation, social skills and assertiveness, and problem-solving skills. The nutrition education was based on the World Health Organization (WHO) latest recommendations on food intake and healthy diet, and each session followed a three-part format: i) Brief review of previous session and participant's adherence to recommendations; ii) Development of the main nutrition education component of each session using an interactive group discussion layout; iii) Resolution of participant's questions and/or concerns, and setting of specific goals. No specific or individualized diet was indicated to participants.

© 2022 Carneiro-Barrera A et al. *JAMA Network Open*.

Physical Exercise[10]

The eight-week physical exercise program consisted of weekly 60-min sessions of supervised moderate intensity aerobic exercise (i.e. 55-65% of the heart rate reserve) and individualized goal-setting consisting of increasing daily step/km per week. In the weekly supervised training sessions, participants were required to walk at a moderate intensity for 60 min wearing a heart rate monitor in order to train themselves to walk at that intensity during the week. With respect to goal-setting, they were advised to increase their daily steps/km by 15% per week, based on their daily steps/km logs.

Sleep Hygiene[10]

The sleep hygiene module comprised four 60-min sessions supervised by a psychologist specialized in the evaluation and treatment of sleep disorders. Owing to most sleep hygiene topics being covered in simultaneous modules, there were four sessions distributed over the eight weeks of the intervention. Generally, this module consisted of sleep hygiene education on causes of sleep disturbances and mistaken sleep related knowledge. Sessions were also based on treating those frequent inadequate sleep habits found in patients with OSA, i.e. sleep restriction, irregular schedule and inappropriate sleep environment.

Smoking Reduction and Cessation[10]

Participants who were current smokers and willing to quit were required to attend a weekly 60-90-min session over eight weeks lead by two clinical psychologists. The intervention was based on the group behavior therapy for smoking cessation by Becoña et al.,[33] which seeks the progressive reduction of tobacco consumption through the use of nicotine and cigarette fading,[34] and behavior change techniques such as information on smoking, self-monitoring, stimulus control, avoidance of withdrawal symptoms, and relapse prevention. Nicotine and cigarette fading has been shown to be the most effective method to reduce and stop smoking with abstinence rates of 86% at the end of treatment and nearly 60% at a 12 month follow-up.[35]

Participants were mainly required to keep a daily record of number of cigarettes smoked and triggers for smoking (self-monitoring), change the type of cigarette smoked to a lesser nicotine content brand each week (30%, 60% and 90% nicotine reductions from baseline), reduce the number of cigarettes smoked by 30% weekly, and avoid smoking in three different situations per week (stimulus control). Through the sessions, other behavior change techniques such as discussion of health consequences of smoking and quitting

(motivation), muscle and cognitive relaxation techniques to address withdrawal symptoms, and identification of high-risk situations for smoking and problem-solving skills (relapse prevention) were used.

Alcohol Intake Avoidance[10]

The INTERAPNEA alcohol intake reduction and avoidance module lasted eight weeks comprising fortnightly sessions of 60 min supervised by two clinical psychologists. Similar to the smoking reduction and cessation module, progressive reduction of alcohol intake in those participants with no alcohol addiction but excessive consumption was pursued. Participants were indicated to reduce the number of units of alcohol consumed per day/week by 30% each week, keeping a log of alcohol-consumption per day including units of alcohol consumed and triggers of consumption. During the sessions, participants received detailed information of alcohol general and specific to OSA health-related consequences. Furthermore, behavior change techniques such as stimulus control, muscle and cognitive relaxation and problem-solving skills related to alcohol consumption were used.

eMethods 4. Assessment of Adherence and Integrity of Intervention and Intervention Adherence

Assessment of Adherence and Integrity of Intervention[10]

Integrity of the intervention and treatment fidelity was evaluated and ensured through the design and implementation of different strategies of process assessment, monitoring and enhancement in order to guarantee internal and external validity of the trial.[36] Regarding the study design and provider of intervention training, we developed a comprehensive hand-book for the qualified INTERAPNEA study intervention providers/professionals/training personnel of each module. Each intervention manual identified the theoretical model of the intervention and provided detailed descriptions of session objectives, treatment guidelines in accordance with each objective (i.e., contents, tasks and activities, recommendations, and timing), participant's homework, and material needed for each session. We also provided each participant with an adapted patient-handbook for each intervention component including descriptions of sessions, and work and logging sheets.

Furthermore, we ensured fidelity in the treatment delivery, receipt, and enactment through the use of these intervention protocols/manuals and monitoring of the implementation. Regarding the treatment delivery, the standardization of the intervention supported the protocol adherence of providers and the treatment differentiation (i.e., the delivery of the target treatment and no other). Similarly, we included a check-list for provider's self-report concerning the achievement of session objectives. With respect to the treatment receipt and enactment, fidelity was assessed and confirmed through different strategies such as the structuring of the intervention around achievement-based objectives, collecting and reviewing of participants self-monitored data (daily step/km log, sleep diaries, alcohol and tobacco consumption records), and information delivery in different formats (e.g., written in the handbooks, and verbal and visual in the sessions).

Apart from the above mention strategies, we also considered complementary approaches in order to reduce participant drop-out rates and increase adherence, such as prevention of commitments or vacation periods, use of well-equipped and conditioned facilities, and supervision by a qualified and certified pair of providers in each session, motivating and supporting participants. Participants' attendance to each intervention session was recorded by providers and causes of absence were recorded through phone-calls.

Intervention Adherence

Most participants in the intervention group attended all sessions of each intervention component (i.e. nutritional behavior change, physical exercise, sleep hygiene, smoking cessation and alcohol avoidance). Of 40 participants and 32 sessions (eight sessions of nutrition; eight of physical exercise; four of sleep hygiene;

© 2022 Carneiro-Barrera A et al. *JAMA Network Open*.

eight of smoking cessation, and four of alcohol avoidance), 24 participants (60%) attended all sessions (100%); 4 (10%) attended 30 sessions (93.8%); 10 (25%) attended 29 sessions (90.6%); and 2 (5%) attended 28 sessions (87.5%). Therefore, 30.8 sessions (96.4%) were attended on average.

eTable 1. Missing Values in Secondary Cardiometabolic Risk End Points

	No.					
	Control			Intervention		
	Baseline	8 wk	6 mo	Baseline	8 wk	6 mo
Glucose	2	15	22	0	0	6
Insulin	3	14	22	0	0	6
HOMA-IR	3	15	22	0	0	6
Total cholesterol	3	15	22	0	0	6
HDL-C	7	19	29	6	4	9
LDL-C	7	19	29	6	4	9
Triglycerides	3	15	22	0	0	6
Apolipoprotein A1	3	19	22	1	6	6
Apolipoprotein B	4	19	22	2	6	6
AST	6	22	23	9	6	7
ALT	2	15	22	0	0	6
γ-GT	2	16	22	0	0	6
Fatty liver index	3	17	22	0	0	6

Abbreviations: CI, confidence interval; BP, blood pressure; HOMA-IR, homeostasis model assessment of insulin resistance; HDL-C, high-density lipoprotein cholesterol; LDL-C, low-density lipoprotein cholesterol; AST, aspartate aminotransferase; ALT, alanine aminotransferase;γ-GT, γ-glutamyltransferase.

© 2022 Carneiro-Barrera A et al. *JAMA Network Open*.

eTable 2. Baseline Characteristics of Study Participants (Per Protocol Approach)

Characteristic[b]	No. (%)[a]	
	**Control (n = 35)**	**Intervention (n = 40)**
Age, mean (SD), y	55.4 (8.9)	52.6 (7.2)
Educational level		
Primary Education	8 (22.9)	10 (25.0)
Secondary Education	6 (17.1)	6 (15.0)
Vocational Education	11 (31.4)	17 (42.5)
Higher Education	10 (28.6)	7 (17.5)
Marital status		
Single	4 (11.4)	2 (5.0)
Married	25 (71.4)	34 (85.0)
Divorced	6 (17.1)	4 (10.0)
Occupational status		
Employed	19 (54.3)	21 (52.5)
Self-employed	6 (17.1)	12 (30.0)
Unemployed	3 (8.6)	5 (12.5)
Retired	7 (20.0)	2 (5.0)
Medical Conditions[c]		
Hypertension	22 (62.9)	27 (67.5)
Diabetes Mellitus II	12 (34.3)	10 (25.0)
Cardiovascular disease	8 (22.9)	7 (17.5)
Other medical conditions	18 (51.4)	26 (65.0)
Medication[c]		
Antihypertensive	21 (60.0)	24 (60.0)
Statins	9 (25.7)	7 (17.5)
Oral antidiabetic	4 (11.4)	2 (5.0)
Insulin	3 (8.6)	1 (2.5)
Beta-blockers	5 (14.3)	5 (12.5)
Polymedication[d]	9 (25.7)	6 (15.0)
Body height, mean (SD), cm	171 (8.6)	172 (6.3)
Body weight status		
Overweight	7 (20.0)	5 (12.5)
Class I obesity	17 (48.6)	19 (47.5)
Class II obesity	10 (28.6)	11 (27.5)
Class III obesity	1 (2.9)	5 (12.5)
Obstructive sleep apnea severity		
Moderate	15 (42.9)	15 (37.5)
Severe	20 (57.1)	25 (62.5)
Time since obstructive sleep apnea diagnosis, mean (SD), y	8.6 (6.0)	6.5 (6.5)
Physical activity, mean (SD), km/day	5.6 (4.2)	6.1 (3.8)
Dietary habits, mean (SD), Food Behavior Checklist score[e]	59.6 (9.4)	59.5 (8.5)
Alcohol consumption		
Never	8 (22.9)	13 (32.5)
Occasionally	11 (31.4)	8 (20.0)
Frequently	9 (25.7)	12 (30.0)
Daily	7 (20.0)	7 (17.5)
Tobacco consumption		
Non-smoker	13 (37.1)	15 (37.5)
Ex-smoker	14 (40.0)	15 (37.5)
Smoker	8 (22.9)	10 (25.0)

[a] No. (%) reported unless otherwise specified.
[b] No significant between-group differences were observed in any of the baseline characteristics.
[c] Participants could have more than one condition or medication.
[d] Defined as the use of five or more medications.
[e] Scores on the Food Behavior Checklist range from 23 to 85, with higher scores indicating healthier dietary pattern.

© 2022 Carneiro-Barrera A et al. *JAMA Network Open*.

eTable 3. Primary and Secondary Sleep-Related End Points (Per Protocol Approach)

	Control (n=35)	Intervention (n=40)	Mean difference between groups (95% CI)[a]
**Primary endpoint**			
Apnea-hypopnea index, events/hr (95% CI)			
At baseline	39.2 (32.4 to 45.9)	41.6 (35.2 to 47.9)	
Change at 8 wk	2.8 (-1.7 to 7.4)	-21.2 (-25.4 to -16.9)	-24.0 (-29.1 to -18.8)[d]
Change at 6 mo	-0.4 (-5.5 to 4.7)	-23.8 (-28.3 to -19.3)	-23.4 (-29.0 to -17.8)[d]
**Secondary endpoints**			
Oxygen desaturation index ≥3%, events/hr (95% CI)			
At baseline	43.0 (35.5 to 50.5)	45.4 (38.4 to 52.5)	
Change at 8 wk	3.4 (-2.3 to 9.2)	-16.0 (-21.4 to -10.6)	-19.5 (-26.0 to -13.0)[d]
Change at 6 mo	-0.4 (-6.8 to 6.1)	-23.5 (-29.2 to 17.7)	-23.1 (-30.2 to -16.0)[d]
Mean SpO_2, % (95% CI)			
At baseline	91.0 (90.0 to 92.0)	91.3 (90.3 to 92.3)	
Change at 8 wk	-0.9 (-2.1 to 0.3)	1.5 (0.4 to 2.6)	2.4 (1.1 to 3.7)[d]
Change at 6 mo	-1.0 (-2.4 to 0.3)	2.6 (1.4 to 3.8)	3.6 (2.2 to 5.1)[d]
SpO_2 Nadir, % (95% CI)			
At baseline	78.4 (75.8 to 81.0)	78.1 (75.6 to 80.5)	
Change at 8 wk	0.1 (-1.9 to 2.1)	2.8 (1.0 to 4.6)	2.7 (0.5 to 4.9)[b]
Change at 6 mo	-1.6 (-3.8 to 0.6)	4.4 (2.5 to 6.4)	6.0 (3.6 to 8.4)[d]
Sleep time with SpO2 <90%, % (95% CI)			
At baseline	11.3 (7.6 to 15.0)	9.1 (5.6 to 12.6)	
Change at 8 wk	1.6 (-2.0 to 5.2)	-4.4 (-7.8 to -1.0)	-6.0 (-10.1 to -1.9)[c]
Change at 6 mo	1.0 (-3.0 to 5.0)	-5.5 (-9.1 to -1.9)	-6.5 (-10.9 to -2.0)[c]
Sleep efficiency, % (95% CI)			
At baseline	86.0 (83.5 to 88.4)	86.0 (83.7 to 88.4)	
Change at 8 wk	-2.0 (-5.4 to 1.4)	5.7 (2.5 to 8.8)	7.7 (3.8 to 11.5)[d]
Change at 6 mo	-2.1 (-5.8 to 1.7)	7.6 (4.2 to 10.9)	9.7 (5.5 to 13.8) [d]
Sleep latency, min (95% CI)			
At baseline	21.2 (16.5 to 25.9)	23.0 (18.6 to 27.4)	
Change at 8 wk	3.2 (-4.4 to 10.8)	-7.1 (-14.2 to 0.04)	-10.2 (-18.8 to -1.7)[b]
Change at 6 mo	5.1 (-3.2 to 13.3)	-11.2 (-18.7 to -3.8)	-16.3 (-25.4 to -7.1)[d]
Wake after sleep onset, min (95% CI)			
At baseline	53.2 (41.2 to 65.2)	47.6 (36.4 to 58.9)	
Change at 8 wk	12.6 (-3.9 to 29.1)	-17.2 (-32.6 to -1.8)	-29.8 (-48.3 to -11.3)[c]
Change at 6 mo	10.7 (-7.5 to 28.9)	-25.8 (-42.0 to -9.5)	-36.5 (-56.4 to -16.3)[d]
N1+N2 sleep, % (95% CI)			
At baseline	64.3 (61.6 to 67.0)	63.4 (60.8 to 65.9)	
Change at 8 wk	3.8 (-0.1 to 7.7)	-6.2 (-9.8 to -2.5)	-10.0 (-14.3 to -5.6)[d]
Change at 6 mo	5.3 (1.0 to 9.6)	-8.9 (-12.8 to -5.1)	-14.2 (-18.9 to -9.5)[d]
N3 sleep, % (95% CI)			
At baseline	20.3 (18.0 to 22.6)	20.4 (18.2 to 22.5)	
Change at 8 wk	-4.0 (-7.4 to -0.7)	3.7 (0.5 to 6.8)	7.7 (3.9 to 11.5)[d]
Change at 6 mo	-7.3 (-11.0 to -3.6)	4.5 (1.2 to 7.8)	11.8 (7.7 to 15.9)[d]
REM sleep, % (95% CI)			
At baseline	15.4 (13.9 to 17.0)	16.2 (14.8 to 17.7)	
Change at 8 wk	0.2 (-2.1 to 2.6)	2.5 (0.3 to 4.7)	2.3 (-0.4 to 4.9)
Change at 6 mo	2.1 (-0.5 to 4.7)	4.5 (2.2 to 6.8)	2.4 (-0.5 to 5.2)
AHI in REM sleep, events/hr (95% CI)			
At baseline	39.8 (33.1 to 46.4)	45.1 (39.0 to 51.3)	
Change at 8 wk	6.1 (-2.0 to 14.2)	-22.6 (-30.2 to -15.1)	-28.7 (-37.9 to -19.6)[d]
Change at 6 mo	-2.8 (-11.8 to 6.2)	-26.6 (-34.6 to -18.6)	-23.8 (-33.6 to -13.9)[d]
AHI in NREM sleep, events/hr (95% CI)			
At baseline	38.5 (31.3 to 45.6)	41.0 (34.3 to 47.7)	
Change at 8 wk	2.6 (-2.4 to 7.6)	-21.0 (-25.7 to -16.3)	-23.6 (-29.2 to -17.9)[d]
Change at 6 mo	-0.7 (-6.3 to 5.0)	-23.6 (-28.5 to -18.6)	-22.9 (-29.1 to -16.7)[d]
Pittsburgh Sleep Quality Index score (95% CI)[e]			
At baseline	8.2 (7.0 to 9.4)	7.2 (6.1 to 8.4)	
Change at 8 wk	-0.3 (-1.4 to 0.8)	-2.8 (-3.8 to -1.8)	-2.5 (-3.7 to -1.2)[d]
Change at 6 mo	0.2 (-1.0 to 1.4)	-3.6 (-4.7 to -2.5)	-3.8 (-5.1 to -2.5)[d]
Epworth Sleepiness Scale score (95% CI)[f]			
At baseline	9.1 (7.6 to 10.7)	10.3 (8.8 to 11.7)	
Change at 8 wk	-0.3 (-2.1 to 1.5)	-4.6 (-6.3 to -2.9)	-4.3 (-6.3 to -2.2)[d]
Change at 6 mo	-1.0 (-3.0 to 1.0)	-6.8 (-8.6 to -5.0)	-5.8 (-8.0 to -3.6)[d]

© 2022 Carneiro-Barrera A et al. *JAMA Network Open*.

Abbreviations: CI, confidence interval; SpO_2, oxygen saturation; AHI, apnea-hypopnea index; REM, rapid eye movement; NREM, non-rapid eye movement.

[a] Using the group × visit interaction term from a linear mixed-effects model including study group, time (baseline, 8 weeks and 6 months), and study group × time as fixed effects and participant as random effects.

[b] $P < 0.05$ from the time × study group interactions.

[c] $P < 0.01$ from the time × study group interactions.

[d] $P < 0.001$ from the time × study group interactions.

[e] Pittsburgh Sleep Quality Index scores range from 0 to 21, with higher scores indicating worse sleep quality.

[f] Epworth Sleepiness Scale scores range from 0 to 24, with higher scores indicating more daytime sleepiness.

© 2022 Carneiro-Barrera A et al. *JAMA Network Open*.

eTable 4. Primary and Secondary Sleep-Related End Points (Change From 8 Weeks to 6 Months)

	Control (n=49)			Intervention (n=40)		
	8 wk Mean (95% CI)	6 mo Mean (95% CI)	Mean change (95% CI)[a]	8 wk Mean (95% CI)	6 mo Mean (95% CI)	Mean change (95% CI)[a]
**Primary endpoint**						
Apnea-hypopnea index, events/hr	43.6 (37.5 to 49.7)	40.3 (34.0 to 46.7)	-3.3 (-8.3 to 1.8)	20.4 (14.0 to 26.9)	17.8 (11.3 to 24.4)	-2.6 (-7.1 to 1.9)
**Secondary endpoints**						
Oxygen desaturation index ≥3%, events/hr	48.3 (41.6 to 55.1)	44.6 (7.5 to 51.7)	-3.7 (-10.1 to 2.6)	29.4 (22.4 to 36.4)	22.0 (14.8 to 29.2)	-7.4 (-13.1 to -1.7)[c]
Mean SpO_2, %	89.6 (88.6 to 90.7)	89.5 (88.4 to 90.6)	-0.2 (-1.5 to 1.1)	92.8 (91.7 to 93.8)	93.9 (92.8 to 94.9)	1.1 (-0.1 to 2.3)
SpO_2 Nadir, %	77.1 (74.3 to 79.8)	75.2 (72.4 to 78.0)	-1.8 (-4.1 to 0.3)	80.9 (78.0 to 83.7)	82.5 (79.5 to 85.4)	1.6 (-0.3 to 3.6)
Sleep time with SpO2 <90%, %	12.9 (9.5 to 16.3)	12.4 (8.8 to 16.0)	-0.5 (-4.5 to 3.4)	4.7 (1.3 to 8.1)	3.6 (0.1 to 7.1)	-1.1 (-4.6 to 2.5)
Sleep efficiency, %	83.9 (81.5 to 86.3)	84.0 (81.3 to 86.6)	0.1 (-3.6 to 3.8)	91.7 (89.4 to 94.0)	93.6 (91.2 to 96.1)	1.9 (-1.4 to 5.3)
Sleep latency, min	24.4 (19.6 to 29.2)	26.0 (20.6 to 31.5)	1.6 (-6.7 to 9.9)	15.9 (11.4 to 20.4)	11.8 (6.9 to 16.6)	-4.1 (-11.7 to 3.4)
Wake after sleep onset, min	66.1 (54.5 to 77.8)	63.8 (50.7 to 76.8)	-2.4 (-20.2 to 15.5)	30.4 (19.3 to 41.5)	21.8 (9.9 to 33.7)	-8.6 (-24.7 to 7.5)
N1+N2 sleep, %	68.3 (65.5 to 71.0)	69.8 (66.7 to 72.9)	1.5 (-2.8 to 5.8)	57.2 (54.6 to 59.8)	54.5 (51.7 to 57.3)	-2.7 (-6.6 to 1.1)
N3 sleep, %	16.3 (14.0 to 18.7)	13.2 (10.5 to 15.8)	-3.2 (-6.9 to 0.6)	24.1 (21.8 to 26.3)	24.8 (22.5 to 27.2)	0.8 (-2.6 to 4.2)
REM sleep, %	15.5 (13.9 to 17.0)	17.2 (15.5 to 19.0)	1.8 (-0.8 to 4.3)	18.7 (17.3 to 20.2)	20.7 (19.2 to 22.3)	2.0 (-0.3 to 4.3)
AHI in REM sleep, events/hr	46.8 (40.4 to 53.2)	38.0 (45.0 to 30.9)	-8.8 (-17.7 to 0.1)	22.5 (16.3 to 28.7)	18.6 (12.0 to 25.1)	-4.0 (-11.9 to 4.0)
AHI in NREM sleep, events/hr	42.8 (49.2 to 36.3)	39.5 (32.7 to 46.2)	-3.3 (-8.9 to 2.2)	20.0 (13.2 to 26.8)	17.4 (10.5 to 24.4)	-2.6 (-7.5 to 2.4)
Pittsburgh Sleep Quality Index score[d]	8.4 (7.2 to 9.5)	9.0 (7.7 to 10.2)	0.6 (-0.6 to 1.8)	4.5 (3.2 to 5.7)	3.7 (2.4 to 4.9)	-0.8 (-1.9 to -0.3)
Epworth Sleepiness Scale score[e]	8.8 (7.3 to 10.3)	8.1 (6.4 to 9.7)	-0.7 (-2.7 to 1.3)	5.7 (4.2 to 7.2)	3.5 (1.9 to 5.0)	-2.2 (-4.0 to -0.4)[b]

Abbreviations: CI, confidence interval; SpO_2, oxygen saturation; AHI, apnea-hypopnea index; REM, rapid eye movement; NREM, non rapid eye movement.

[a] Using post-hoc test (pairwise comparison) in a linear mixed-effects model including study group, time (baseline, 8 weeks and 6 months), and study group × time as fixed effects and participant as random effects.

[b] $P < 0.05$.

[c] $P < 0.01$.

[d] Pittsburgh Sleep Quality Index scores range from 0 to 21, with higher scores indicating worse sleep quality.

[e] Epworth Sleepiness Scale scores range from 0 to 24, with higher scores indicating more daytime sleepiness.

© 2022 Carneiro-Barrera A et al. *JAMA Network Open*.

eTable 5. Secondary Body Composition and Cardiometabolic Risk End Points (Per Protocol Approach)

	Control (n=35)	Intervention (n=40)	Mean difference between groups (95% CI)[a]
**Body composition**			
Body weight, kg (95% CI)			
At baseline	94.0 (87.8 to 100.1)	102.2 (96.8 to 107.6)	
Change at 8 wk	-0.2 (-1.9 to 1.5)	-7.1 (-8.6 to -5.5)	-6.9 (-8.7 to -5.0)[d]
Change at 6 mo	-1.1 (-3.0 to 0.8)	-6.9 (-8.5 to -5.2)	-5.8 (-7.8 to -3.7)[d]
Body mass index, kg/m^2 (95% CI)			
At baseline	33.7 (35.5 to 31.9)	35.0 (36.7 to 33.4)	
Change at 8 wk	-0.2 (-0.8 to 0.4)	-2.5 (-3.0 to -1.9)	-2.3 (-2.9 to -1.6)[d]
Change at 6 mo	-0.6 (-1.2 to 0.1)	-2.4 (-3.0 to -1.8)	-1.8 (-2.5 to -1.1)[d]
Neck circumference, cm (95% CI)			
At baseline	45.1 (43.9 to 46.2)	45.0 (43.9 to 46.2)	
Change at 8 wk	-0.3 (-0.9 to 0.3)	-2.3 (-2.8 to -1.7)	-2.0 (-2.6 to -1.3)[d]
Change at 6 mo	0.2 (-0.5 to 0.9)	-2.9 (-3.5 to -2.3)	-3.1 (-3.9 to -2.4)[d]
Chest circumference, cm (95% CI)			
At baseline	116.1 (112.9 to 119.4)	118.0 (115.0 to 121.1)	
Change at 8 wk	0.5 (-0.8 to 1.9)	-3.4 (-4.6 to -2.1)	-3.9 (-5.4 to -2.4)[d]
Change at 6 mo	0.7 (-0.8 to 2.2)	-4.1 (-5.5 to -2.8)	-4.8 (-6.4 to -3.1)[d]
Waist circumference, cm (95% CI)			
At baseline	116.6 (112.5 to 120.8)	119.0 (115.1 to 122.9)	
Change at 8 wk	-0.1 (-1.6 to 1.4)	-6.9 (-8.4 to -5.5)	-6.8 (-8.5 to -5.1)[d]
Change at 6 mo	0.3 (-1.4 to 2.2)	-8.8 (-10.3 to -7.2)	-9.1 (-11.0 to -7.2)[d]
Fat mass, kg (95% CI)			
At baseline	33.0 (29.8 to 36.3)	34.9 (31.8 to 37.9)	
Change at 8 wk	1.5 (-0.2 to 3.2)	-2.9 (-4.5 to -1.3)	-4.4 (-6.3 to -2.4)[d]
Change at 6 mo	0.2 (-1.7 to 2.2)	-6.5 (-8.2 to -4.8)	-6.7 (-8.8 to -4.6)[d]
Visceral adipose tissue, g (95% CI)			
At baseline	1021.8 (929.2 to 1114.4)	1017.3 (930.7 to 1104.0)	
Change at 8 wk	42.1 (-44.6 to 128.8)	-106.2 (-187.3 to -25.2)	-148.3 (-245.7 to -51.0)[c]
Change at 6 mo	-13.9 (-110.6 to 82.8)	-268.5 (-354.5 to -182.5)	-254.5 (-360.6 to -148.4)[d]
Lean mass, kg (95% CI)			
At baseline	60.5 (57.6 to 63.5)	63.0 (60.2 to 65.8)	
Change at 8 wk	-2.1 (-3.5 to -0.8)	-2.7 (-4.0 to -1.5)	-0.6 (-2.1 to 0.9)
Change at 6 mo	-1.2 (-2.7 to 0.3)	0.3 (-1.0 to 1.7)	1.5 (-0.2 to 3.1)
**Blood pressure**			
Systolic BP, mm Hg (95% CI)			
At baseline	142.6 (137.8 to 147.4)	143.7 (139.2 to 148.2)	
Change at 8 wk	-0.9 (-5.4 to 3.6)	-7.9 (-12.1 to -3.7)	-7.0 (-12.1 to -2.0)[c]
Change at 6 mo	2.2 (-2.8 to 7.3)	-13.9 (-18.4 to -9.4)	-16.1 (-21.6 to -10.6)[d]
Diastolic BP, mm Hg (95% CI)			
At baseline	81.7 (78.0 to 85.4)	84.0 (80.5 to 87.5)	
Change at 8 wk	0.5 (-4.4 to 5.4)	-5.7 (-10.3 to -1.2)	-6.2 (-11.7 to -0.7)[b]
Change at 6 mo	2.4 (-3.0 to 7.8)	-7.4 (-12.2 to -2.5)	-9.7 (-15.7 to -3.8)[c]
Mean BP, mm Hg (95% CI)			
At baseline	102.0 (98.4 to 105.6)	103.9 (100.5 to 107.3)	
Change at 8 wk	0.03 (-4.1 to 4.2)	-6.5 (-10.3 to -2.6)	-6.5 (-11.1 to -1.9)c
Change at 6 mo	2.3 (-2.3 to 6.9)	-9.6 (-13.6 to -5.5)	-11.8 (-16.9 to -6.8)[d]
**Glucose metabolism**			
Glucose, mg/dl (95% CI)			
At baseline	100.1 (93.3 to 106.9)	95.5 (89.1 to 101.9)	
Change at 8 wk	0.6 (-4.4 to 5.7)	-6.7 (-11.4 to -2.1)	-7.3 (-13.0 to -1.7)[b]
Change at 6 mo	4.5 (-1.1 to 10.0)	-6.6 (-11.6 to -1.7)	-11.1 (-17.2 to -4.9)[d]
Insulin, IU/ml (95% CI)			
At baseline	14.7 (12.2 to 17.3)	13.0 (10.6 to 15.4)	
Change at 8 wk	1.5 (-0.7 to 3.6)	-4.9 (-6.9 to -2.9)	-6.4 (-8.8 to -4.0)[d]
Change at 6 mo	0.2 (-2.2 to 2.5)	-5.2 (-7.3 to -3.1)	-5.3 (-7.9 to -2.7)[d]
HOMA-IR index (95% CI)			
At baseline	3.5 (2.6 to 4.4)	3.2 (2.3 to 4.0)	
Change at 8 wk	0.5 (-0.7 to 1.6)	-1.3 (-2.4 to -0.3)	-1.8 (-3.1 to -0.5)[c]
Change at 6 mo	0.4 (-0.9 to 1.7)	-1.4 (-2.5 to -0.3)	-1.8 (-3.2 to -0.4)[b]

(continued)

© 2022 Carneiro-Barrera A et al. *JAMA Network Open*.

(continued)			
	**Control (n=35)**	**Intervention (n=40)**	**Mean difference between groups (95% CI)[a]**
**Lipid metabolism**			
Total cholesterol, mg/dl (95% CI)			
At baseline	174.1 (162.5 to 185.6)	189.6 (178.9 to 200.2)	
Change at 8 wk	6.3 (-4.7 to 17.2)	-19.4 (-29.4 to -9.4)	-25.7 (-37.8 to -13.5)[d]
Change at 6 mo	3.9 (-8.1 to 15.8)	-16.6 (-27.2 to -6.0)	-20.5 (-33.6 to -7.4)[c]
HDL-C, mg/dl (95% CI)			
At baseline	42.4 (39.0 to 45.9)	47.1 (44.0 to 50.2)	
Change at 8 wk	2.4 (-0.5 to 5.3)	0.2 (-2.1 to 2.5)	-2.2 (-5.2 to 0.8)
Change at 6 mo	0.9 (-2.4 to 4.1)	3.0 (0.5 to 5.4)	2.1 (-1.2 to 5.4)
LDL-C, mg/dl (95% CI)			
At baseline	113.8 (103.1 to 124.4)	119.4 (110.1 to 128.8)	
Change at 8 wk	-1.6 (-10.3 to 7.1)	-15.0 (-21.9 to -8.0)	-13.3 (-22.4 to -4.3)[c]
Change at 6 mo	2.9 (-6.8 to 12.7)	-14.3 (-21.5 to -7.0)	-17.2 (-27.1 to -7.3)[c]
Triglycerides, mg/dl (95% CI)			
At baseline	153.6 (131.7 to 175.5)	129.5 (109.1 to 149.8)	
Change at 8 wk	4.4 (-14.1 to 22.9)	-24.5 (-41.3 to -7.6)	-28.9 (-49.4 to -8.4)[c]
Change at 6 mo	16.0 (-4.3 to 36.2)	-23.8 (-41.7 to -5.8)	-39.7 (-61.8 to -17.5)[d]
Apolipoprotein A1, mg/dl (95% CI)			
At baseline	125.9 (119.4 to 132.4)	131.0 (125.0 to 137.1)	
Change at 8 wk	6.2 (-0.2 to 12.5)	-0.6 (-6.5 to 5.3)	-6.8 (-13.9 to 0.3)
Change at 6 mo	0.9 (-5.8 to 7.5)	9.5 (3.4 to 15.4)	8.6 (1.4 to 15.9)[b]
Apolipoprotein B, mg/dl (95% CI)			
At baseline	94.4 (87.4 to 101.3)	102.5 (95.9 to 109.0)	
Change at 8 wk	2.5 (-4.2 to 9.2)	-11.9 (-18.2 to -5.6)	-14.4 (-22.0 to -6.9)[d]
Change at 6 mo	-1.3 (-8.3 to 5.7)	-15.1 (-21.5 to -8.8)	-13.9 (-21.6 to -6.1)[d]
**Liver function**			
AST, IU/l (95% CI)			
At baseline	25.3 (21.9 to 28.7)	25.3 (22.4 to 28.3)	
Change at 8 wk	0.8 (-3.7 to 5.3)	-2.4 (-6.2 to 1.4)	-3.2 (-8.0 to 1.6)
Change at 6 mo	-0.6 (-5.2 to 4.0)	-4.8 (-8.6 to -0.9)	-4.1 (-9.0 to 0.8)
ALT, IU/l (95% CI)			
At baseline	28.6 (24.0 to 33.2)	29.6 (25.3 to 33.9)	
Change at 8 wk	0.8 (-4.5 to 6.0)	-4.0 (-8.9 to 0.8)	-4.8 (-10.7 to 1.1)
Change at 6 mo	0.3 (-5.5 to 6.1)	-7.1 (-12.3 to -2.0)	-7.4 (-13.8 to -1.0)[b]
γ-GT, IU/l (95% CI)			
At baseline	40.9 (30.1 to 51.6)	38.2 (28.2 to 48.2)	
Change at 8 wk	4.5 (-3.9 to 12.9)	-11.2 (-18.9 to -3.5)	-15.7 (-25.0 to -6.3)[c]
Change at 6 mo	1.6 (-7.6 to 10.8)	-14.0 (-22.2 to -5.9)	-15.6 (-25.6 to -5.5)[c]
Fatty liver index (95% CI)			
At baseline	84.4 (78.4 to 90.5)	86.2 (80.5 to 91.8)	
Change at 8 wk	-1.2 (-6.1 to 3.6)	-13.7 (-9.3 to -18.0)	-12.4 (-17.8 to -7.1)[d]
Change at 6 mo	0.2 (-5.1 to 5.4)	-17.5 (-12.9 to -22.1)	-17.7 (-23.4 to -12.0)[d]

Abbreviations: CI, confidence interval; BP, blood pressure; HOMA-IR, homeostasis model assessment of insulin resistance; HDL-C, high-density lipoprotein cholesterol; LDL-C, low-density lipoprotein cholesterol; AST, aspartate aminotransferase; ALT, alanine aminotransferase;γ-GT, γ-glutamyltransferase.

SI conversion factors: To convert glucose to millimoles per liter, multiply by 0.05551. To convert insulin to picomoles per liter, multiply by 6.945. To convert total, high-density lipoprotein, and low-density lipoprotein cholesterol to millimoles per liter, multiply by 0.02586. To convert triglycerides to millimoles per liter, multiply by 0.01129. To convert apolipoprotein A1 and B to gram per liter, multiply by 0.01. To convert aspartate aminotransferase to micro-katal per liter, multiply by 0.017. To convert aspartate aminotransferase and γ-glutamyltransferase to micro-katal per liter, multiply by 0.017.

[a] Using the group × visit interaction term from a linear mixed-effects model including study group, time (baseline, 8 weeks and 6 months), and study group × time as fixed effects and participant as random effects.

[b] $P < 0.05$ from the time × study group interactions.

[c] $P < 0.01$ from the time × study group interactions.

[d] $P < 0.001$ from the time × study group interactions.

eTable 6. Secondary Body Composition and Cardiometabolic Risk End Points (Change From 8 Weeks to 6 Months)

	Control (n=49)			Intervention (n=40)		
	8 wk Mean (95% CI)	6 mo Mean (95% CI)	Mean change (95% CI)[a]	8 wk Mean (95% CI)	6 mo Mean (95% CI)	Mean change (95% CI)[a]
**Body composition**						
Body weight, kg	99.3 (94.1 to 104.4)	98.4 (93.2 to 103.5)	-0.9 (-2.8 to 0.9)	96.2 (90.5 to 101.8)	96.4 (90.7 to 102.1)	0.2 (-1.4 to 1.9)
Body mass index, kg/m^2	33.7 (32.2 to 35.3)	33.3 (31.8 to 34.9)	-0.4 (-1.0 to 0.2)	32.6 (30.9 to 34.3)	32.6 (30.9 to 34.3)	0.1 (-0.5 to 0.6)
Neck circumference, cm	45.1 (44.0 to 46.2)	45.6 (44.5 to 46.8)	0.5 (-0.1 to 1.2)	42.8 (41.6 to 43.9)	42.1 (40.9 to 43.3)	-0.7 (-1.2 to -0.1)[b]
Chest circumference, cm	117.8 (115.0 to 120.7)	118.0 (115.1 to 121.0)	0.2 (-1.3 to 1.7)	114.7 (111.5 to 117.8)	113.9 (110.8 to 117.1)	-0.8 (-2.1 to 0.6)
Waist circumference, cm	117.7 (114.1 to 121.3)	118.2 (114.5 to 121.9)	0.5 (-1.2 to 2.2)	117.7 (114.1 to 121.3)	118.2 (114.5 to 121.9)	-1.8 (-3.3 to -0.3)[b]
Fat mass, kg	35.2 (32.3 to 38.2)	34.0 (31.0 to 37.0)	-1.3 (-3.2 to 0.7)	32.0 (28.9 to 35.1)	28.4 (25.2 to 31.6)	-3.6 (-5.3 to -1.9)[d]
Visceral adipose tissue, g	1081.7 (995.0 to 1168.5)	1022.9 (930.1 to 1115.6)	-58.9 (-154.7 to 37.0)	911.1 (823.3 to 998 to9)	748.9 (658.0 to 839.8)	-162.2 (-248.1 to -76.3)[d]
Lean mass, kg	58.7 (56.0 to 61.3)	59.6 (62.2 to 56.9)	0.9 (-0.5 to 2.4)	60.3 (57.5 to 63.1)	63.3 (60.5 to 66.2)	3.0 (1.7 to 4.4)[d]
**Blood pressure**						
Systolic BP, mm Hg	141.6 (137.2 to 146.0)	144.9 (140.2 to 149.6)	3.3 (-1.6 to 8.3)	135.7 (131.3 to 140.2)	129.8 (125.2 to 134.4)	-6.0 (-10.4 to -1.5)[c]
Diastolic BP, mm Hg	82.3 (78.7 to 85.9)	84. 1 (80.1 to 88.1)	1.8 (-3.5 to 7.1)	78.3 (74.9 to 81.7)	76.7 (73.0 to 80.3)	-1.6 (-6.4 to 3.2)
Mean BP, mm Hg	102.1 (98.8 to 105.5)	104.4 (100.8 to 108.1)	2.3 (-2.2 to 6.8)	97.4 (94.1 to 100.7)	94.3 (90.9 to 97.8)	-3.1 (-7.1 to 1.0)
**Glucose metabolism**						
Glucose, mg/dl	102.1 (95.7 to 108.4)	105.6 (99.0 to 112.1)	3.5 (-2.1 to 9.1)	88.8 (82.3 to 95.3)	88.9 (82.3 to 95.5)	-0.1 (-4.8 to 5.1)
Insulin, IU/ml	15.7 (13.4 to 18.0)	14.3 (11.9 to 16.8)	-1.4 (-3.7 to 1.0)	8.1 (5.8 to 10.4)	7.8 (5.4 to 10.2)	-0.3 (-2.4 to 1.8)
HOMA-IR index	4.0 (3.1 to 4.8)	3.8 (2.9 to 4.8)	-0.1 (-1.4 to 1.1)	1.8 (1.0 to 2.6)	1.8 (0.9 to 2.6)	-0.1 (-1.2 to 1.0)
**Lipid metabolism**						
Total cholesterol, mg/dl	182.8 (171.4 to 194.2)	182.1 (170.0 to 194.2)	-0.7 (-12.8 to 11.4)	170.2 (158.7 to 181.6)	172.9 (161.1 to 184.8)	2.8 (-7.9 to 13.5)
HDL-C, mg/dl	46.6 (43.4 to 49.8)	45.3 (41.9 to 48.8)	-1.3 (-4.2 to 1.7)	47.3 (44.1 to 50.5)	50.1 (46.8 to 53.3)	2.8 (0.3 to 5.2)[b]
LDL-C, mg/dl	113.5 (104.0 to 123.0)	121.2 (110.8 to 131.5)	7.7 (-2.3 to 17.6)	104.5 (95.2 to 113.9)	105.3 (95.7 to 114.9)	0.7 (-7.3 to 8.8)
Triglycerides, mg/dl	158.1 (136.3 to 179.8)	164.8 (141.8 to 187.8)	6.8 (-16.3 to 29.8)	105.0 (83.2 to 126.8)	105.7 (83.2 to 128.2)	0.7 (-19.7 to 21.1)
Apolipoprotein A1, mg/dl	133.8 (127.5 to 140.1)	129.0 (122.5 to 135.5)	-4.8 (-11.7 to 2.1)	130.4 (124.1 to 136.7)	140.5 (134.2 to 146.9)	10.1 (4.0 to 16.3)[d]
Apolipoprotein B, mg/dl	98.5 (91.5 to 105.5)	95.2 (88.0 to 102.3)	-3.3 (-10.6 to 4.0)	90.6 (97.6 to 83.6)	87.3 (80.3 to 94.3)	-3.3 (-9.8 to 3.2)
**Liver function**						
AST, IU/l	26.0 (22.8 to 29.3)	24.5 (21.2 to 27.8)	-1.5 (-5.8 to 2.8)	22.9 (20.0 to 25.9)	20.6 (17.5 to 23.6)	-2.4 (-6.2 to 1.4)
ALT, IU/l	29.5 (25.0 to 33.9)	28.7 (23.8 to 33.5)	-0.8 (-6.5 to 5.0)	25.6 (21.3 to 29.9)	22.5 (17.9 to 27.0)	-3.1 (-8.3 to 2.0)
γ-GT, IU/l	47.8 (37.6 to 57.9)	44.7 (34.1 to 55.2)	-3.1 (-12.4 to 6.2)	27.0 (16.7 to 37.3)	24.2 (13.6 to 34.7)	-2.8 (-11.0 to 5.3)
Fatty liver index	84.2 (78.7 to 89.7)	85.6 (79.9 to 91.2)	1.4 (-3.9 to 6.7)	72.5 (66.9 to 78.0)	68.6 (63.0 to 74.3)	-3.8 (-8.4 to 0.8)

Abbreviations: CI, confidence interval; BP, blood pressure; HOMA-IR, homeostasis model assessment of insulin resistance; HDL-C, high-density lipoprotein cholesterol; LDL-C, low-density lipoprotein cholesterol; AST, aspartate aminotransferase; ALT, alanine aminotransferase;γ-GT, γ-glutamyltransferase.

SI conversion factors: To convert glucose to millimoles per liter, multiply by 0.05551. To convert insulin to picomoles per liter, multiply by 6.945. To convert total, high-density lipoprotein, and low-density lipoprotein cholesterol to millimoles per liter, multiply by 0.02586. To convert triglycerides to millimoles per liter, multiply by 0.01129. To convert apolipoprotein A1 and B to gram per liter, multiply by 0.01. To convert aspartate aminotransferase to micro-katal per liter, multiply by 0.017. To convert aspartate aminotransferase and γ-glutamyltransferase to micro-katal per liter, multiply by 0.017.

[a] Using post-hoc test (pairwise comparison) in a linear mixed-effects model including study group, time (baseline, 8 weeks and 6 months), and study group × time as fixed effects and participant as random effects.

[b] $P < 0.05$.

[c] $P < 0.01$.

[d] $P < 0.001$.

© 2022 Carneiro-Barrera A et al. *JAMA Network Open*.

eTable 7. Health-Related Quality of Life End Points

	**Control**		**Intervention**		**Mean difference between groups (95% CI)[a]**
	**N**	**Mean (95% CI)**	**N**	**Mean (95% CI)**	
**Intention-to-treat approach**					
Sleep Apnea Quality of Life Index score[e]					
At baseline	49	4.7 (4.4 to 5.0)	40	4.8 (4.5 to 5.1)	
Change at 8 wk	49	0.1 (-0.3 to 0.4)	40	0.8 (0.5 to 1.1)	0.8 (0.4 to 1.2)[d]
Change at 6 mo	49	0.1 (-0.3 to 0.5)	40	1.1 (0.7 to 1.5)	1.0 (0.6 to 1.5)[d]
SF-36 Physical-component summary score[f]					
At baseline	49	45.3 (42.4 to 48.1)	40	46.5 (43.3 to 49.6)	
Change at 8 wk	49	2.5 (-1.3 to 6.2)	40	6.0 (2.4 to 9.7)	3.5 (-0.8 to 7.8)
Change at 6 mo	49	-0.02 (-4.2 to 4.1)	40	6.5 (2.6 to 10.3)	6.5 (1.9 to 11.1)[c]
SF-36 Mental-component summary score[f]					
At baseline	49	46.7 (43.5 to 49.9)	40	48.3 (44.8 to 51.9)	
Change at 8 wk	49	1.6 (-3.0 to 6.1)	40	3.6 (-0.8 to 8.1)	2.1 (-3.2 to 7.3)
Change at 6 mo	49	-0.1 (-5.2 to 4.9)	40	6.1 (1.4 to 10.8)	6.2 (0.6 to 11.9)[b]
**Per-protocol approach**					
Sleep Apnea Quality of Life Index score[e]					
At baseline	35	4.7 (4.4 to 5.1)	40	4.8 (4.5 to 5.1)	
Change at 8 wk	35	0.04 (-0.3 to 0.4)	40	0.8 (0.5 to 1.1)	0.8 (0.4 to 1.2)[d]
Change at 6 mo	35	0.1 (-0.4 to 0.5)	40	1.1 (0.7 to 1.5)	1.0 (0.6 to 1.5)[d]
SF-36 Physical-component summary score[f]					
At baseline	35	45.9 (42.7 to 49.0)	40	46.5 (43.5 to 49.4)	
Change at 8 wk	35	2.2 (-1.6 to 6.0)	40	6.0 (2.5 to 9.6)	3.9 (-0.4 to 8.1)
Change at 6 mo	35	-0.4 (-4.6 to 3.9)	40	6.5 (2.7 to 10.3)	6.9 (2.2 to 11.5)[c]
SF-36 Mental-component summary score[f]					
At baseline	35	47.9 (44.2 to 51.7)	40	48.3 (44.8 to 51.9)	
Change at 8 wk	35	0.9 (-3.9 to 5.6)	40	3.6 (-0.8 to 8.1)	2.8 (-2.6 to 8.1)
Change at 6 mo	35	-0.9 (-6.2 to 4.3)	40	6.1 (1.4 to 10.8)	7.0 (1.2 to 12.8)[b]

Abbreviations: CI, confidence interval.

[a] Using the group × visit interaction term from a linear mixed-effects model including study group, time (baseline, 8 weeks and 6 months), and study group × time as fixed effects and participant as random effects.

[b] $P < 0.05$ from the time × study group interactions.

[c] $P < 0.01$ from the time × study group interactions.

[d] $P < 0.001$ from the time × study group interactions.

[e] Sleep Apnea Quality of Life Index scores range from 1 to 7, with higher scores indicating better health-related quality of life.

[f] Medical Outcomes Study 36-Item Short-Form Health Survey (SF-36) scores range from 0 to 100, with higher scores indicating better health-related quality of life with respect to either the physical or mental component.

© 2022 Carneiro-Barrera A et al. *JAMA Network Open*.

eTable 8. Health-Related Quality of Life End Points (Change From 8 Weeks to 6 Months)

	Control (n=49)			Intervention (n=40)		
	**8 wk Mean (95% CI)**	**6 mo Mean (95% CI)**	**Mean change (95% CI)[a]**	**8 wk Mean (95% CI)**	**6 mo Mean (95% CI)**	**Mean change (95% CI)[a]**
Sleep Apnea Quality of Life Index score[b]	4.8 (4.5 to 5.1)	4.8 (4.4 to 5.1)	0.02 (-0.4 to 0.4)	5.6 (5.3 to 5.9)	5.9 (5.6 to 6.2)	0.3 (-0.1 to 0.6)
SF-36 Physical-component summary score[c]	47.7 (44.5 to 50.9)	45.2 (41.7 to 48.7)	-2.5 (-6.8 to 1.8)	52.5 (49.4 to 55.6)	52.9 (49.6 to 56.2)	0.4 (-3.4 to 4.3)
SF-36 Mental-component summary score[c]	48.3 (44.6 to 52.0)	46.6 (42.5 to 50.7)	-1.7 (-6.9 to 3.5)	51.9 (48.4 to 55.5)	54.4 (50.6 to 58.2)	2.4 (-2.2 to 7.1)

Abbreviations: CI, confidence interval.

[a] Using post-hoc test (pairwise comparison) in a linear mixed-effects model including study group, time (baseline, 8 weeks and 6 months), and study group × time as fixed effects and participant as random effects.

[b] Sleep Apnea Quality of Life Index scores range from 1 to 7, with higher scores indicating better health-related quality of life.

[c] Medical Outcomes Study 36-Item Short-Form Health Survey (SF-36) scores range from 0 to 100, with higher scores indicating better health-related quality of life with respect to either the physical or mental component.

© 2022 Carneiro-Barrera A et al. *JAMA Network Open*.

eTable 9. Physical Activity and Dietary Behavior End Points

	Control		Intervention		Mean difference between groups (95% CI)[a]
	N	Mean (95% CI)	N	Mean (95% CI)	
**Intention-to-treat approach**					
Physical activity, km/day					
At baseline	49	5.2 (3.9 to 6.4)	40	6.1 (4.8 to 7.5)	
Change at 8 wk	49	0.4 (-1.2 to 2.0)	40	8.8 (7.3 to 10.4)	8.4 (6.5 to 10.2)[b]
Change at 6 mo	49	-0.9 (-2.7 to 0.9)	40	6.0 (4.3 to 7.6)	6.9 (4.9 to 8.9)[b]
Dietary habits, Food Behavior Checklist score[c]					
At baseline	49	59.1 (56.8 to 61.4)	40	59.5 (56.9 to 62.0)	
Change at 8 wk	49	3.3 (0.6 to 5.9)	40	12.0 (9.5 to 14.5)	8.7 (5.7 to 11.7)[b]
Change at 6 mo	49	1.5 (-1.4 to 4.4)	40	9.2 (6.5 to 11.9)	7.7 (4.4 to 10.9)[b]
**Per-protocol approach**					
Physical activity, km/day					
At baseline	35	5.6 (4.1 to 7.1)	40	6.1 (4.8 to 7.5)	
Change at 8 wk	35	0.2 (-1.4 to 1.9)	40	8.8 (7.2 to 10.4)	8.6 (6.7 to 10.5)[b]
Change at 6 mo	35	-1.1 (-2.9 to 0.8)	40	6.0 (4.3 to 7.7)	7.1 (5.0 to 9.1)[b]
Dietary habits, Food Behavior Checklist score[c]					
At baseline	35	59.6 (57.0 to 62.3)	40	59.5 (57.0 to 62.0)	
Change at 8 wk	35	3.1 (0.4 to 5.9)	40	12.0 (9.5 to 14.5)	8.9 (5.8 to 11.9)[b]
Change at 6 mo	35	1.5 (-1.5 to 4.5)	40	9.2 (6.5 to 11.9)	7.7 (4.4 to 11.0)[b]

Abbreviations: CI confidence interval.
[a] Using the group × visit interaction term from a linear mixed-effects model including study group, time (baseline, 8 weeks and 6 months), and study group × time as fixed effects and participant as random effects.
[b] $P < 0.001$ from the time × study group interactions.
[c] Scores on the Food Behavior Checklist range from 23 to 85, with higher scores indicating healthier dietary pattern.

© 2022 Carneiro-Barrera A et al. *JAMA Network Open*.

eTable 10. Physical Activity and Dietary Behavior End Points (Change From 8 Weeks to 6 Months)

	Control (n=49)			Intervention (n=40)		
	**8 wk Mean (95% CI)**	**6 mo Mean (95% CI)**	**Mean change (95% CI)[a]**	**8 wk Mean (95% CI)**	**6 mo Mean (95% CI)**	**Mean change (95% CI)[a]**
Physical activity, km/day	5.6 (4.2 to 7.0)	4.3 (2.7 to 5.8)	-1.3 (-3.2 to 0.5)	15.0 (13.6 to 16.3)	12.1 (10.7 to 13.6)	-2.8 (-4.5 to -1.2)[c]
Dietary habits, Food Behavior Checklist score[d]	62.4 (59.9 to 64.9)	60.6 (57.9 to 63.4)	-1.8 (-4.8 to 1.2)	71.5 (68.9 to 74.0)	68.6 (66.0 to 71.3)	-2.8 (-5.5 to -0.1)[b]

Abbreviations: CI, confidence interval.

[a] Using post-hoc test (pairwise comparison) in a linear mixed-effects model including study group, time (baseline, 8 weeks and 6 months), and study group × time as fixed effects and participant as random effects.

[b] $P < 0.01$

[c] $P < 0.001$

[d] Scores on the Food Behavior Checklist range from 23 to 85, with higher scores indicating healthier dietary pattern.

© 2022 Carneiro-Barrera A et al. *JAMA Network Open*.

eFigure 1. Obstructive Sleep Apnea Severity at Baseline and 8 Weeks After Intervention in the Intervention Group

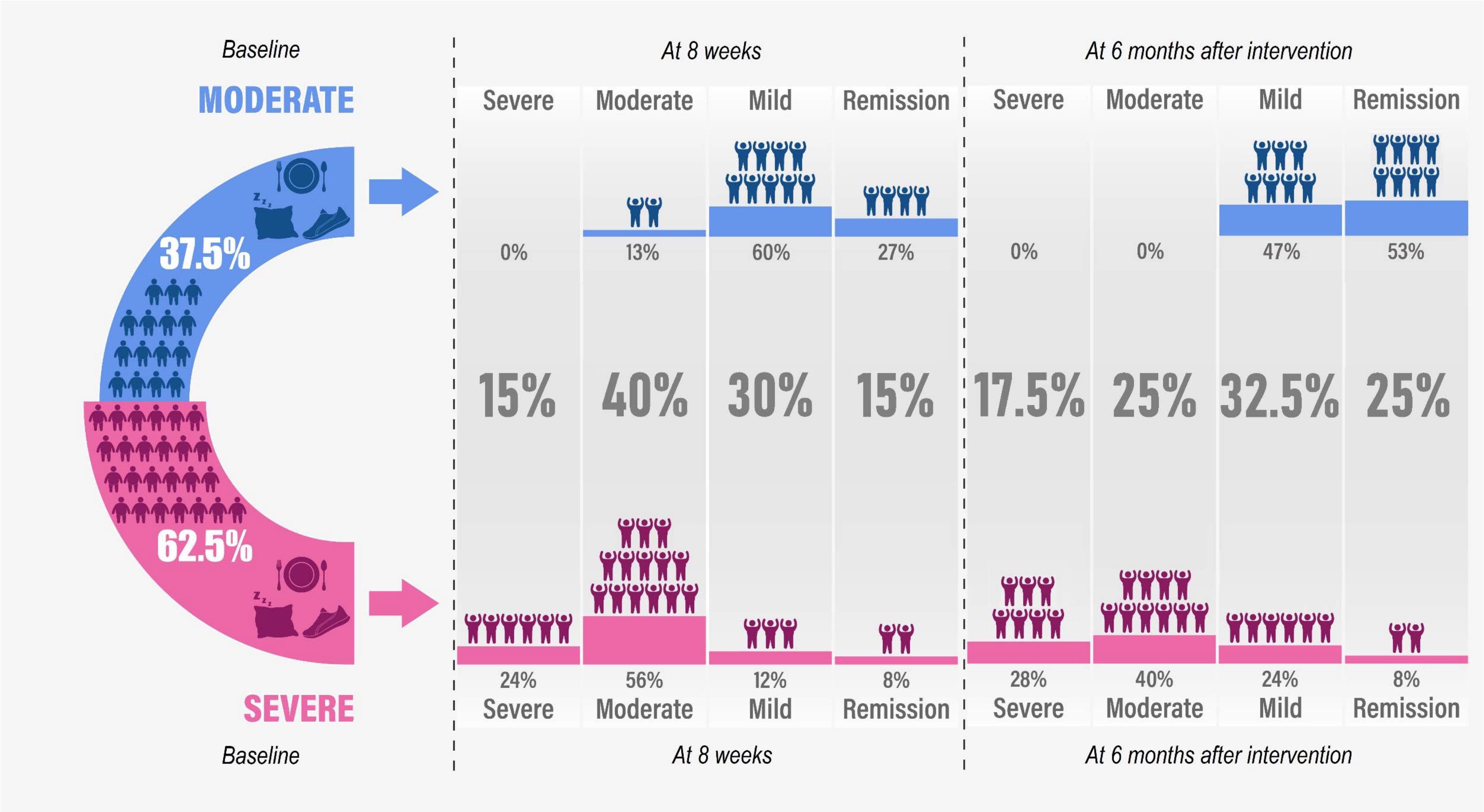

© 2022 Carneiro-Barrera A et al. *JAMA Network Open*.

eFigure 2. Apnea-Hypopnea Index End Point (Change From 8 Weeks to 6 Months)

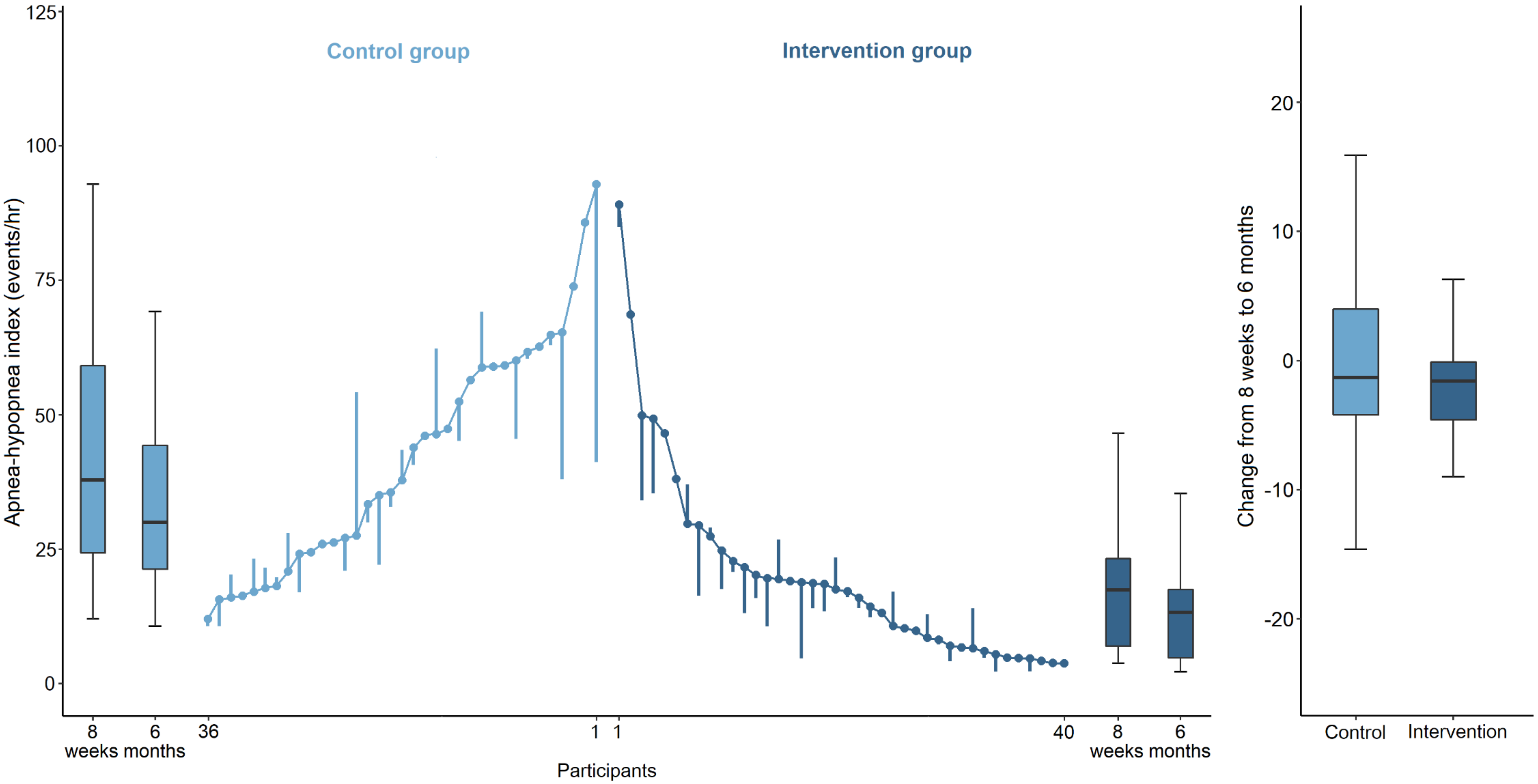

The ends of the boxes in the boxplots are located at the first and third quartiles, with the black line in the middle illustrating the median. Whiskers extend to the upper and lower adjacent values, the location of the furthest point within a distance of 1.5 interquartile ranges from the first and third quartiles. The parallel line plot contains 1 vertical line for each patient which extends from their 8-week value to their 6-month value. Descending lines indicate an improvement in the outcome. Eight-week values are placed in ascending order for the control group and descending order for the intervention group. The apnea-hypopnea index indicates the number of apnea and hypopnea events per hour of sleep (0-5 is classified as normal; 5-14, mild OSA; 15-30, moderate OSA; >30, severe OSA; a change of at least 15 is considered clinically meaningful and would move a patient 2 levels from severe to mild with established benefit for health).

© 2022 Carneiro-Barrera A et al. *JAMA Network Open*.

eFigure 3. Association Between Changes in Apnea-Hypopnea Index Over Time and Changes in Body Mass Index

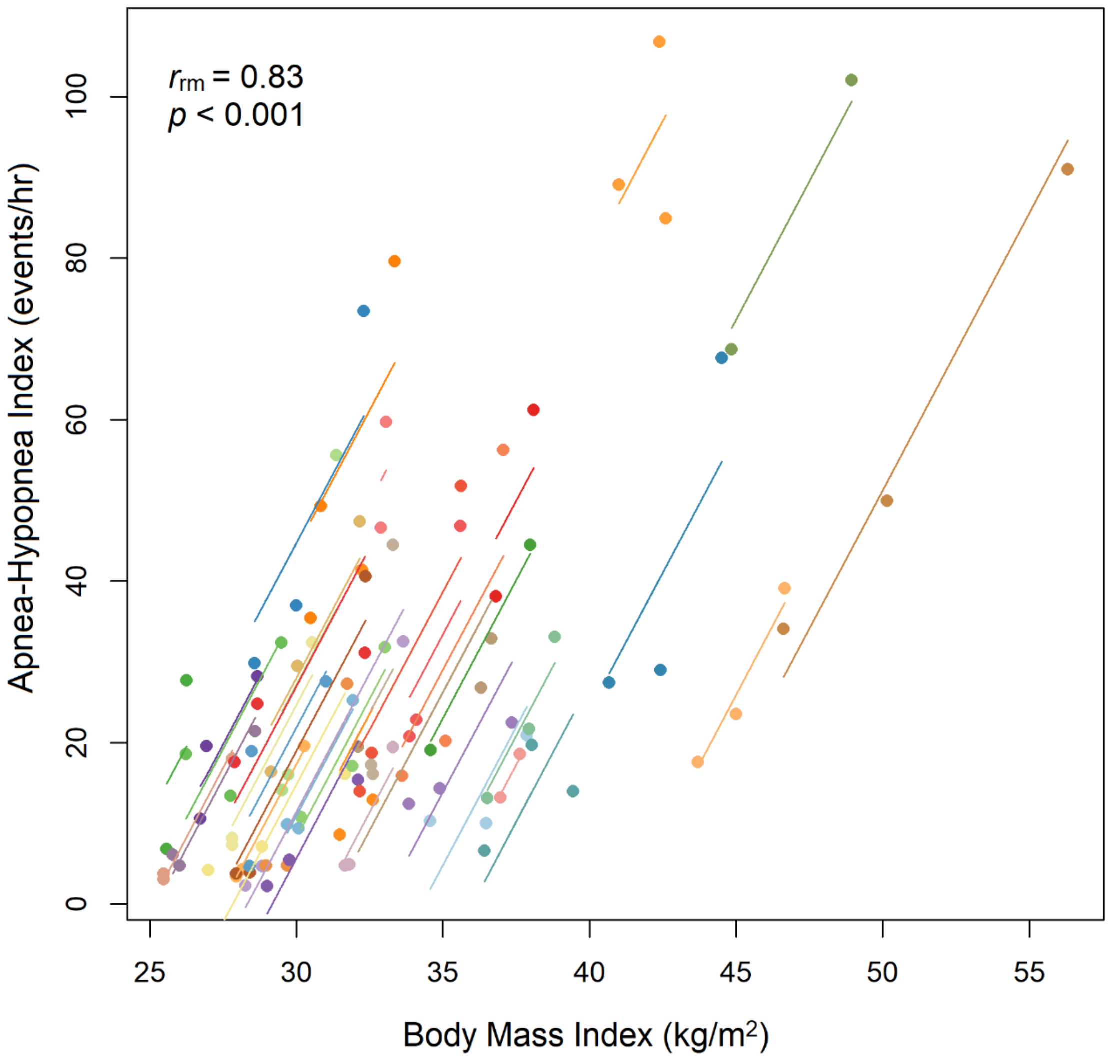

Each dot represents one of three separate observations (baseline, 8 weeks and 6 months after intervention) of apnea-hypopnea index and body mass index for a participant. Observations from the same participant are given the same color, with corresponding lines to show the repeated measures correlation fit for each participant.

© 2022 Carneiro-Barrera A et al. *JAMA Network Open*.

eReferences

1. Gartlehner G, Hansen RA, Nissman D, Lohr KN, Carey TSJ. A simple and valid tool distinguished efficacy from effectiveness studies. Clin Epidemiol 2006;59:1040-8.
2. Thorpe KE, Zwarenstein M, Oxman AD, et al. A pragmatic-explanatory continuum indicator summary (PRECIS): a tool to help trial designers. J Clin Epidemiol 2009;62:464-75.
3. Singal AG, Higgins PDR, Waljee AK. A Primer on effectiveness and efficacy trials. Clin Transl Gastroenterol 2014;5:e45.
4. Senaratna CV, Perret JL, Lodge CJ, et al. Prevalence of obstructive sleep apnea in the general population: a systematic review. Sleep Med Rev 2017;34:70-81.
5. Basoglu OK, Tasbakan MS. Gender differences in clinical and polysomnographic features of obstructive sleep apnea: a clinical study of 2827 patients. Sleep Breath 2018;22:241-9.
6. Carneiro-Barrera A, Díaz-Román A, Guillén-Riquelme A, Buela-Casal G. Weight loss and lifestyle interventions for obstructive sleep apnoea in adults: systematic review and meta-analysis. Obes Rev 2019;20:750-62.
7. Robertson C, Avenell A, Boachie C, et al. Should weight loss and maintenance programmes be designed differently for men? A systematic review of long-term randomised controlled trials presenting data for men and women: the ROMEO project. Obes Res Clin Pract 2016;10:70-84.
8. Williams RL, Wood LG, Collins CE, Callister R. Effectiveness of weight loss interventions — is there a difference between men and women: a systematic review. Obes Rev 2015;16:171-86.
9. Harreiter J, Kautzky-Willer A. Sex and gender differences in prevention of type 2 diabetes. Front Endocrinol 2018;9:220.
10. Carneiro-Barrera A, Amaro-Gahete FJ, Díaz-Román, A, et al. Interdisciplinary weight loss and lifestyle intervention for obstructive sleep apnoea in adults: rationale, design and methodology of the INTERAPNEA study. Nutrients 2019;11:2227.
11. Kapur VK, Auckley DH, Chowdhuri S, et al. Clinical practice guideline for diagnostic testing for adult obstructive sleep apnea: an American Academy of Sleep Medicine clinical practice guideline. J Clin Sleep Med 2017;13:479-504.
12. Boudewyns A, Sforza E, Zamagni M, Krieger J. Respiratory effort during sleep apneas after interruption of long-term CPAP treatment in patients with obstructive sleep apnea. Chest 1996;110:120-7.

© 2022 Carneiro-Barrera A et al. *JAMA Network Open*.

13. Berry RB, Budhiraja R, Gottlieb DJ, et al. Rules for scoring respiratory events in sleep: update of the 2007 AASM manual for the scoring of sleep and associated events. Deliberations of the sleep apnea definitions task force of the American Academy of Sleep Medicine. J Clin Sleep Med 2012;8:597-619.
14. Jasper HH. Report of the committee on methods of clinical examination in electroencephalography: 1957. Electroencephalogr Clin Neurophysiol 1958;10:370-5.
15. Rechtschaffen A, Kales A. A manual of standardized terminology, techniques and scoring system of sleep stages in human subjects. Los Angeles, CA, USA: Brain Information Service, Brain Research Institute, University of California, 1968.
16. Mokhlesi B, Varga AW. Obstructive sleep apnea and cardiovascular disease: REM sleep matters! Am J Respir Crit Care Med 2018;197:554-6.
17. Mokhlesi B, Finn LA, Hagen EW, et al. Obstructive sleep apnea during REM sleep and hypertension: results of the Wisconsin Sleep Cohort. Am J Respir Crit Care Med 2014;190:1158-67.
18. Appleton SL, Vakulin A, Martin SA, et al. Hypertension is associated with undiagnosed obstructive sleep apnea during rapid eye movement (REM) sleep. Chest 2016;150:495-505.
19. Buysee DJ, Reynolds 3rd CF, Monk TH, Berman SR, Kupfer DJ. The Pittsburgh Sleep Quality Index: a new instrument for psychiatric practice and research. Psychiatry Res 1989;28:193-213
20. Johns MW. A new method for measuring daytime sleepiness: the Epworth sleepiness scale. Sleep 1991;14:540-5.
21. Marfell-Jones MJ, Stewart AD, de Ridder JH. International standards for anthropometric assessment. Wellington, New Zealand: International Society for the Advancement of Kinanthropometry, 2012.
22. Matthews DR, Hosker JP, Rudenski AS, Nayor BA, Treacher DF, Turner RL. Homeostatic model assessment: insulin resistance and beta-cell function from fasting plasma glucose and insulin concentrations in man. Diabetologia 1985;28:412-9.
23. Bedogni G, Bellentani S, Miglioli L, et al. The Fatty Liver Index: a simple and accurate predictor of hepatic steatosis in the general population. BMC Gastroenterol 2006;6:33.
24. Flemons WW, Reimer MA. Development of a disease-specific health-related quality of life questionnaire for sleep apnea. Am J Respir Crit Care Med 1998;158:494-503.
25. Ware JE Jr, Sherboume CD. The MOS 36-item short-form health survey (SF-36). I. Conceptual framework and item selection. Med Care 1992;30:473-83

© 2022 Carneiro-Barrera A et al. *JAMA Network Open*.

26. Ware JE Jr. SF-36 Health survey: manual and interpretation guide. Boston: Nimrod Press, 1993.
27. Banna JC, Townsend MS. Assessing factorial and convergent validity and reliability of a food behaviour checklist for Spanish-speaking participants in US Department of Agriculture nutrition education programmes. Public Health Nutr 2011;14:1165-76.
28. Senaratna CV, English DR, Currier D, et al. Sleep apnoea in Australian men: disease burden, co-morbidities, and correlates from the Australian longitudinal study on male health. BMC Public Health 2016;16:51-61.
29. Veasey SC, Rosen IM. Obstructive sleep apnea in adults. N Engl J Med 2019;380:1442-9.
30. Epstein LJ, Kristo D, Strollo PJ, et al. Clinical guideline for the evaluation, management and long-term care of obstructive sleep apnea in adults. J Clin Sleep Med 2009;5:263-76.
31. Hudgel DW, Patel SR, Ahasic AM, et al. The role of weight management in the treatment of adult obstructive sleep apnea: an official American Thoracic Society clinical practice guideline. Am J Respir Crit Care Med 2018;198:e70-e87.
32. Prochaska JO, Velicer WF. The transtheoretical model of health behavior change. Am J Health Promot 1997;12:38-48.
33. Becoña E. Programa para dejar de fumar [Program for smoking cessation]. Vigo, España: Nova Galicia Edicións, 2007.
34. Foxx RM, Brown RA. Nicotine fading, self-monitoring for cigarette abstinence or controlled smoking. J Applied Behav Anal 1979;12:115-25.
35. Becoña E, García MP. Nicotine fading and smokeholding methods to smoking cessation. Psychol Rep 1993;73:779-86.
36. Borrelli B. The assessment, monitoring, and enhancement of treatment fidelity in public health clinical trials. J Public Health Dent 2011;71:52-63.

© 2022 Carneiro-Barrera A et al. *JAMA Network Open*.
