# Supplementary material for: Effect of an Interdisciplinary Weight Loss and Lifestyle Intervention on Obstructive Sleep Apnea Severity: The INTERAPNEA Randomized Clinical Trial
Source: JAMA Netw Open. 2022 Apr 22;5(4):e228212. doi: 10.1001/jamanetworkopen.2022.8212 (PMC9034401; doi:10.1001/jamanetworkopen.2022.8212)
Supplement: Supplement 3. — Data Sharing Statement [file jamanetwopen-e228212-s003.pdf]

Data Sharing Statement

Carneiro-Barrera. Effect of an Interdisciplinary Weight Loss and Lifestyle Intervention on Obstructive Sleep Apnea Severity. *JAMA Netw Open*. Published April 22, 2022. doi:10.1001/jamanetworkopen.2022.8212

Data
Data available: Yes
Data types: Deidentified participant data
How to access data: Data will be made available upon request to the Principal Investigator: acarneiro@ugr.es
When available: beginning date: 06-01-2022

Supporting Documents
Document types: None

Additional Information
Who can access the data: Researchers whose proposed use of the data has been approved by the INTERAPNEA Publications Committee.
Types of analyses: For scientific publication
Mechanisms of data availability: With a signed data use agreement with the INTERAPNEA Research Team and the University of Granada
